# Design, synthesis and biological evaluation of marine naphthoquinone-naphthol derivatives as potential anticancer agents

**DOI:** 10.1080/14756366.2024.2412865

**Published:** 2024-10-15

**Authors:** Yujuan Li, Luyou Yelv, Xiaoqiu Wu, Ning Liu, Yamin Zhu

**Affiliations:** a Department of Chemistry, College of Food Science and Technology, Shanghai Ocean University, Shanghai, China; b Marine Biomedical Science and Technology Innovation Platform of Lin-gang Special Area, Shanghai, China; c Department of Marine Bio-Pharmacology, College of Food Science and Technology, Shanghai Ocean University, Shanghai, China; d International Research Center for Food & Health, Shanghai Ocean University, Shanghai, China; e Collaborative Innovation Center of Seafood Deep Processing, Ministry of Education, Shanghai Ocean University, Shanghai, China

**Keywords:** Naphthoquinone-naphthol derivatives, marine, anticancer activities, EGFR

## Abstract

1’-Hydroxy-4’,8,8’-trimethoxy-[2,2’-binaphthalene]-1,4-dione (compound **5**), a secondary metabolite recently discovered in marine fungi, demonstrates promising cytotoxic and anticancer potential. However, knowledge regarding the anticancer activities and biological mechanisms of its derivatives remains limited. Herein, a series of novel naphthoquinone-naphthol derivatives were designed, synthesised, and evaluated for their anticancer activity against cancer cells of different origins. Among these, Compound **13**, featuring an oxopropyl group at the *ortho*-position of quinone group, exhibited the most potent inhibitory effects on HCT116, PC9, and A549 cells, with IC_50_ values decreasing from 5.27 to 1.18 μM (4.5-fold increase), 6.98 to 0.57 μM (12-fold increase), and 5.88 to 2.25 μM (2.6-fold increase), respectively, compared to compound **5**. Further mechanistic studies revealed that compound **13** significantly induced cell apoptosis by increasing the expression levels of cleaved caspase-3 and reducing Bcl-2 proteins through downregulating the EGFR/PI3K/Akt signalling pathway, leading to the inhibition of proliferation in HCT116 and PC9 cells. The present findings suggest this novel naphthoquinone-naphthol derivative may hold potential as an anticancer therapeutic lead.

## GRAPHICAL ABSTRACT

**Figure:**
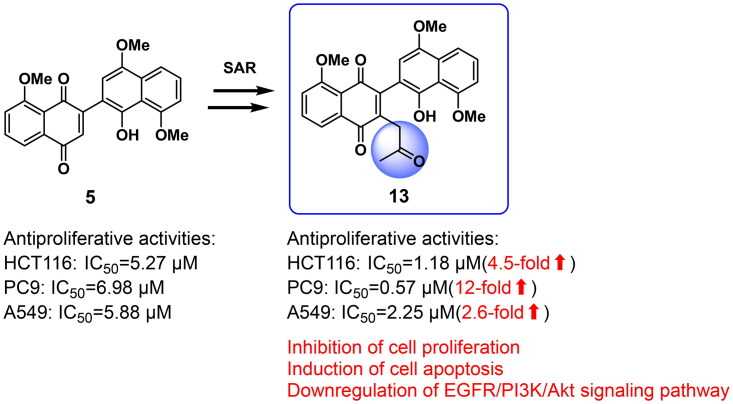

## Introduction

Cancer poses a persistent threat to human health, with rising morbidity and high mortality rates^1^^,^^2^. Over the past decades, numerous anticancer drugs have been developed through combinatorial synthesis and molecular docking studies for chemotherapy^3^. However, the substantial side effects induced by anticancer drugs, along with the potential for tumour resistance and recurrence, continue to pose a formidable challenge^4^^,^^5^. Consequently, there is an urgent imperative to discover innovative anticancer drugs.

To date, over 60% of approved and pre-new drug application (NDA) chemotherapeutic candidates are derived from or closely related to natural products^6^. Marine natural products (MNPs) stand out for their diverse chemical structures and remarkable pharmacological activities, shaped by the unique environmental conditions of marine ecosystems, including high pressure, salt, low oxygen, and limited light^7^. Among these, secondary metabolites from marine fungi have garnered attention, not only leading to new cytotoxic chemicals but also providing insights for refining structures to develop improved chemotherapeutic drugs^8^. For example, plinabulin, a ketopyrazine compound derived from marine *Aspergillus*, is currently undergoing Phase III clinical trials for non-small cell lung cancer (NSCLC) and shows promise in preventing chemotherapy-induced neutropenia^9^. Additionally, varioloid A and B^10^, scopararane I^11^, physcion^12^, and others^13–15^ are isolated from various marine fungi, demonstrating potent cytotoxic activity against a wide range of cancer cell lines.

In our ongoing exploration for novel small-molecule anticancer agents with distinct structures from MNPs, our focus shifted to 1′-hydroxy-4′,8,8′-trimethoxy-[2,2′-binaphthalene]-1,4-dione (**5**), isolated from the marine fungus *Hypoxylon rubiginosum* FS521 in 2020^16^. This compound features a unique naphthoquinone-naphthol skeleton, while also demonstrating promising cytotoxic activity. Despite reports on the structure and cytotoxic activity of compound **5**, there is still limited understanding of its derivatives, including their anticancer activity and biological mechanisms. Thus, in this study, a structure-activity relationship (SAR)-based synthetic strategy was employed to synthesise structurally refined analogues towards potential MNP-derived anticancer agents. In fact, the naphthoquinone moiety, widely found in naturally occurring and biologically active compounds, has been associated with the pharmacological properties of some known anticancer agents^17^. Naphthoquinone derivatives, such as napabucasin (BBI-608), sepantronium bromide (YM-155), and menadione (vitamin K3), have demonstrated significant anticancer effects against metastatic colorectal carcinoma, lymphoma, and liver cancer, respectively, both as monotherapies and in combination with other anticancer agents^18^. Notably, napabucasin has advanced to Phase III clinical trials, while sepantronium bromide has reached Phase II clinical trials^19^. Dinaphthoquinone (**6**), bearing two naphthoquinone rings, was initially designed but showed a significant decrease in effect. Thereby, the naphthoquinone-naphthol scaffold was identified as responsible for the anti-proliferative activity, which led to further structure modifications. Notably, compound **13**, featuring an oxopropyl group at the *ortho*-position of the quinone group, was found to exhibit the most potent inhibitory effects (HCT116, IC_50_ = 1.18 μM; PC9, IC_50_ = 0.57 μM; A549, IC_50_ = 2.25 μM), with activity increases of 4.5-fold, 12-fold, and 2.6-fold, respectively, compared to compound **5**. The intriguing activity of compound **13** stimulated us to conduct further studies on its biological mechanisms.

## Results and discussion

### Chemistry

**Scheme 1.**
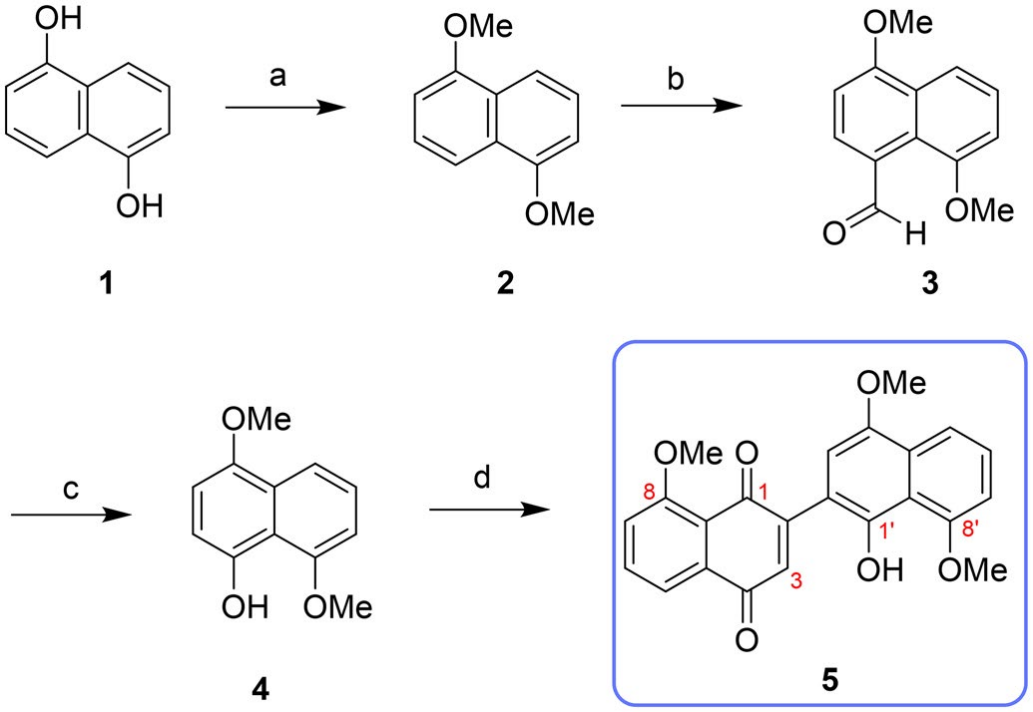
Synthetic of compound **5_._** Reagent and conditions: (a) dimethyl sulphate, K_2_CO_3_, acetone, reflux, 12 h, 87%; (b) POCl_3_, CHCl_3_, DMF, r.t., 10 h, 97%; (c) step 1. *m*-CPBA, CH_2_Cl_2_, r.t., 4 h; step 2. K_2_CO_3_, THF, MeOH, 0 °C, 5 h, 61%; (d) *p*-chloranil, CH_2_Cl_2_, r.t., 48 h, 84%.

Compound **5** was synthesised via a streamlined four-step procedure for the initial evaluation of its anti-proliferative activity (Scheme 1). Commercially available 1,5-dihydroxynaphthalene (**1**) underwent methylation with dimethyl sulphate and K_2_CO_3_ in acetone, yielding 1,5-dimethoxynaphthalene (**2**). Then, a Vilsmeier-Haack reaction was conducted by adding compound **2** to a mixture of phosphorus oxychloride (POCl_3_) and *N*,*N*-dimethylformamide (DMF) in CHCl_3_, producing 4,8-dimethoxy-1-naphthaldehyde (**3**). This compound underwent Baeyer-Villiger oxidative rearrangement with 3-chloroperoxybenzoic acid (*m*-CPBA), followed by hydrolysis with K_2_CO_3_ to obtain 4,8-dimethoxynaphthalene-1-ol (**4**). A one-step oxidative coupling of compound **4** with *p*-chloranil in CH_2_Cl_2_ produced naphthoquinone-naphthol (**5**) with a good yield, differing from the reported two-step sequence involving oxidative dimerisation and monodemethylation.

**Scheme 2.**
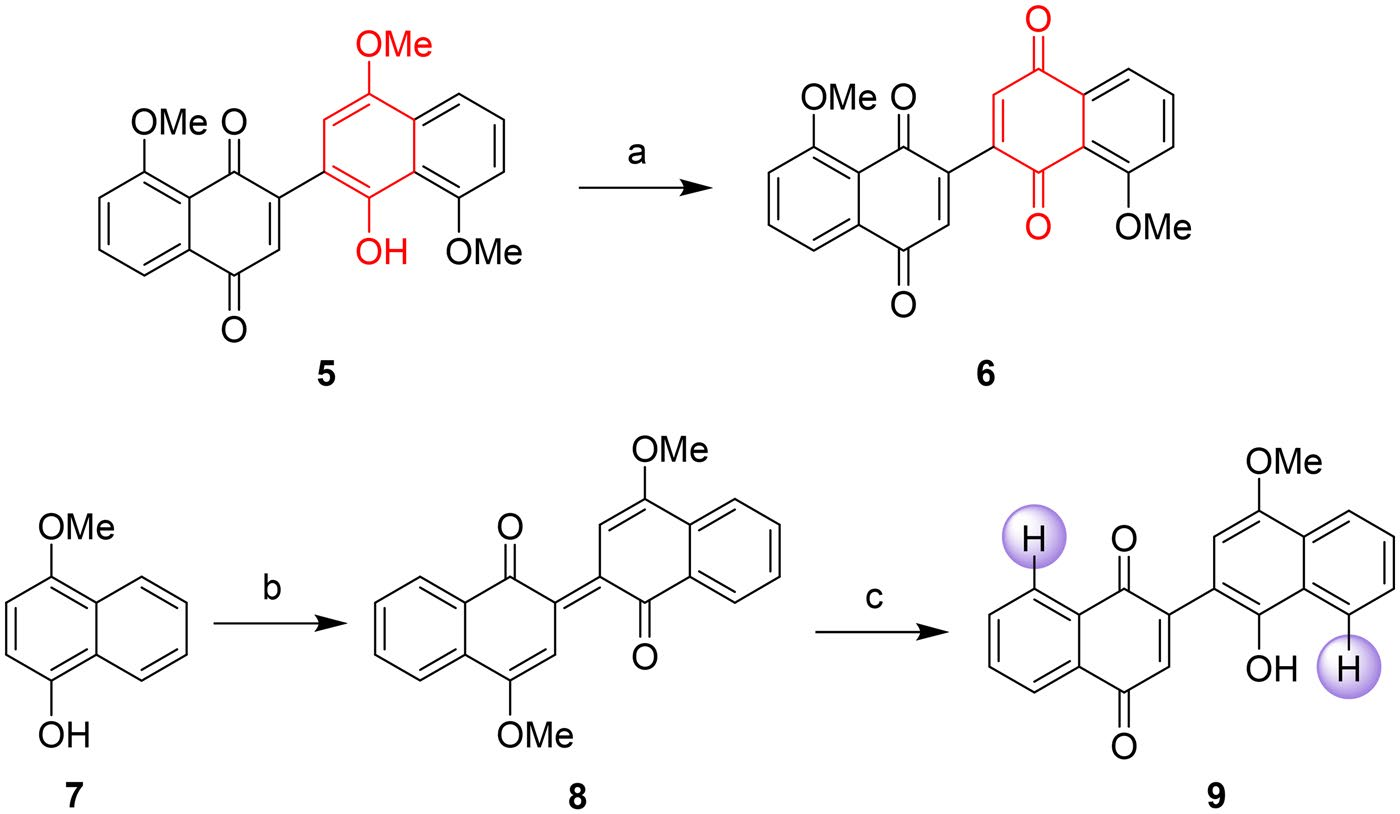
Synthesis of dinaphthoquinone **6** and naphthoquinone-naphthol derivative **9**. Reagent and conditions: (a) CAN, CH_3_CN, H_2_O, 0 °C, 45 min, 85%; (b) *p*-chloranil, CH_2_Cl_2_, r.t., 24 h, 90%; (c) SnO_2_, CH_2_Cl_2_, r.t., 24 h, 69%.

To examine the importance of the naphthol moiety^20^ and two methoxy groups^21^ for the anticancer efficacy of the entire structure, we initially designed dinaphthoquinone (**6**) and demethoxylated naphthoquinone-naphthol (**9**). Compound **6** was prepared by oxidising compound **5** with cerium ammonium nitrate (CAN) in CH_3_CN. Commercially available compound **7** underwent oxidative dimerisation with *p*-chloranil in CH_2_Cl_2_ to produce compound **8**, followed by monodemethylation using SnO_2_ in CH_2_Cl_2_ to obtain compound **9** (Scheme 2).

**Scheme 3.**
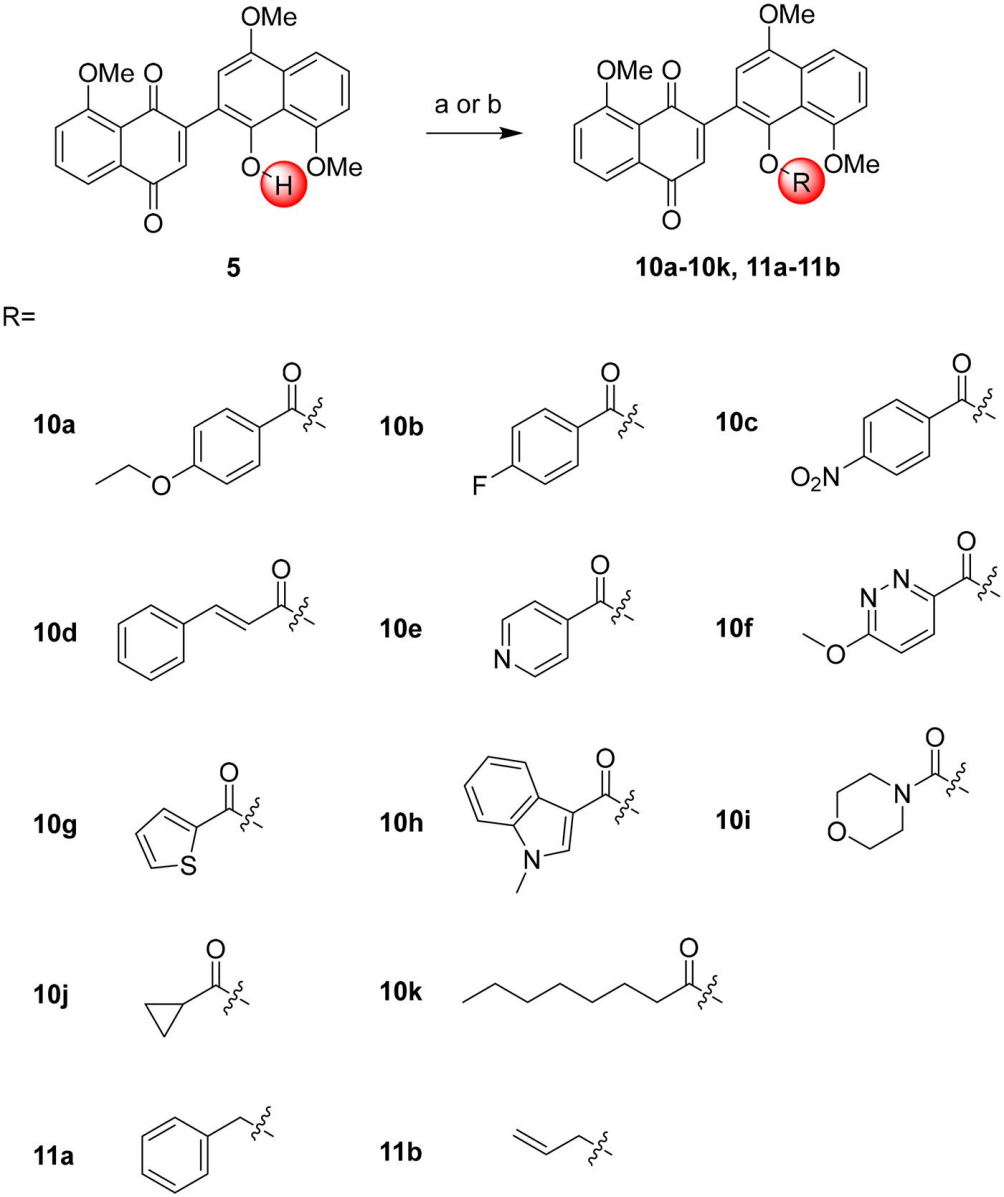
Synthesis of naphthoquinone-naphthol derivatives **10a–10k** and **11a–11b**. Reagent and conditions: (a) EDCI, 4-DMAP, HOAt, CH_2_Cl_2_, r.t. or reflux, 7–12 h, 40%–98%; (b) K_2_CO_3_, acetone, reflux, 10–14 h, 51%–75%.

Subsequently, compound **5** underwent esterification with various carboxylic acids using 4-dimethylaminopyridine (4-DMAP), 3-(((ethylimino)methylene)amino)-*N*,*N*-dimethylpropan-1-amine hydrochloride (EDCI), and 3*H*-[1,2,3]triazolo[4,5-*b*]pyridin-3-ol (HOAt) in CH_2_Cl_2_, yielding **10a**-**10k**. Additionally, nucleophilic substitution with alkyl bromides led to the synthesis of ether derivatives **11a**-**11b** (Scheme 3).

**Scheme 4.**
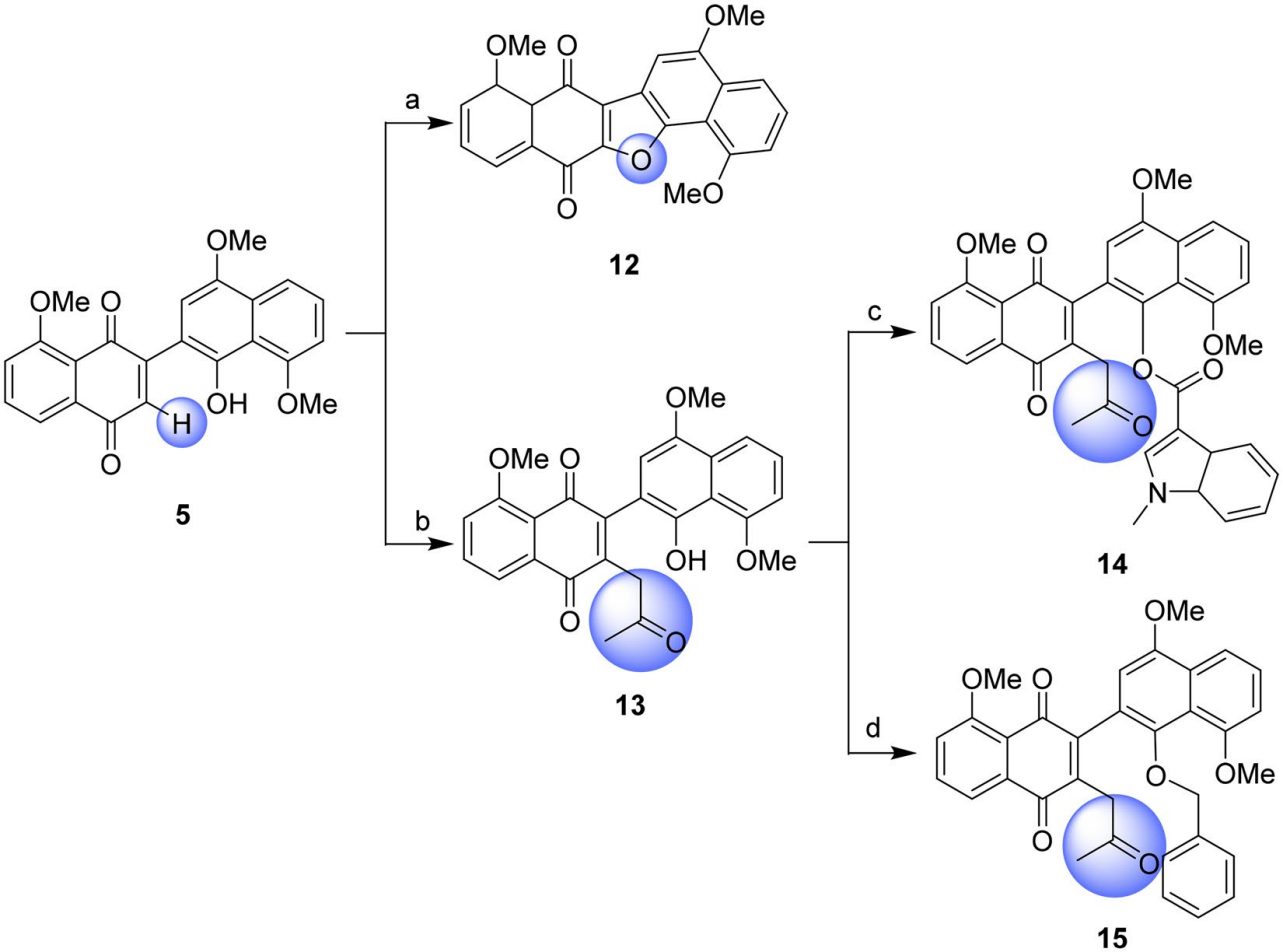
Synthesis of naphthoquinone-naphthol derivatives **12**–**15**. Reagent and conditions: (a) *p*-chloranil, toluene, reflux, 48 h, 88%; (b) bromoacetic acid, Cs_2_CO_3_, KI, acetone, r.t., 5 h, 67%; (c) 1-methyl-1*H*-indole-3-carboxylic acid, EDCI, 4-DMAP, HOAt, CH_2_Cl_2_, reflux, 72 h, 40%; (d) benzyl bromide, K_2_CO_3_, acetone, reflux, 14 h, 75%.

Finally, the *ortho*-position of the quinone group (3-position) was explored by using two strategies (Scheme 4). Compound **5** underwent oxidative cyclisation with *p*-chloranil, resulting in the formation of the five-fused-ring structure (**12**). Alternatively, *ortho*-substitution with an oxopropyl group was achieved in the presence of bromoacetic acid, Cs_2_CO_3_ and KI, yielding compound **13**. Further esterification or etherification of compound **13** generated compound **14** or **15**, respectively. Remarkably, both **14** and **15** featured two substituent groups: an oxopropyl group at the *ortho*-position in the naphthoquinone moiety, along with either an indole ester group or a phenyl ether group replacing the free phenolic hydroxyl group in the naphthol moiety.

### In vitro antiproliferative activity and SAR

Building upon the initial cytotoxic activity observed in compound **5**, we assessed the antiproliferative effects of naphthoquinone-naphthol derivatives against colon cancer (HCT116) and NSCLC (PC9, A549) cell lines using the CCK-8 assay. As illustrated in Table 1, compound **5** demonstrated remarkable inhibitory effect across all three cancer cell lines, with 72 h IC_50_ values ranging from 5.27 to 6.98 μM. It was included as a positive reference compound for further investigation.

**Table 1.** Antiproliferative activities of compounds **5**–**15**.

Compd.	Antiproliferative Activity (IC_50_ ± SD, μM)^a^		
	HCT116	PC9	A549
**5**	5.27 ± 0.56	6.98 ± 0.50	5.88 ± 0.31
**6**	>20	>20	>20
**9**	>20	19.00 ± 0.85	>20
**10a**	>20	>20	>20
**10b**	4.34 ± 0.32	3.64 ± 0.19	17.90 ± 1.63
**10c**	5.66 ± 0.46	>20	>20
**10d**	>20	>20	>20
**10e**	8.57 ± 7.37	4.56 ± 0.53	16.87 ± 1.27
**10f**	12.75 ± 1.59	6.22 ± 0.24	3.11 ± 0.40
**10g**	5.37 ± 0.37	7.47 ± 0.41	>20
**10h**	3.07 ± 0.22	4.24 ± 0.39	>20
**10i**	10.99 ± 0.97	5.26 ± 0.67	3.01 ± 0.45
**10j**	8.19 ± 0.40	7.50 ± 0.36	8.22 ± 0.16
**10k**	5.99 ± 0.33	9.60 ± 0.22	3.85 ± 0.73
**11a**	9.61 ± 0.24	8.57 ± 0.33	12.54 ± 1.09
**11b**	>20	>20	>20
**12**	>5^b^	>5^b^	>5^b^
**13**	**1.18 ± 0.09**	**0.57 ± 0.16**	**2.25 ± 0.27**
**14**	>20	>20	>20
**15**	>20	>20	>20

^a^ Cells were treated with different concentrations of compounds for 72 h to obtain the IC_50_ values. Data are shown as the mean ± SD (*n* = 3).

^b^ Due to its poor solubility, the maximum measured was 5 μM.

As discussed in the chemistry section, modifying compound **5** to compound **6**, characterised by two linked naphthoquinone rings, resulted in a complete loss of inhibitory activity against all three cancer cell lines at a concentration of 20 μM (Table 1). This is consistent with previous studies, where binaphthoquinone (BQ) exhibited cytotoxicity against A549 with IC_50_ values of >10 μM^22^. Compound **9**, lacking two methoxy groups at 8 and 8′ positions, also exhibited a significant decrease in effect, with all IC_50_ values > 19 μM across the three cancer cell lines. These findings highlight the critical role of the naphthoquinone-naphthol skeleton with two methoxy groups in anticancer activity, prompting further synthesis efforts to modify the phenolic hydroxyl group through esterification or etherification.

In the esterification study, we synthesised various compounds, including benzoate esters (**10a–10c)**, cinnamate ester (**10d)**, heteroaromatic esters (**10e–10h)**, and non-aromatic esters (**10i–10k)**. Compound **10a**, featuring an electron-donating 4-OEt substituent, showed no effect on three cancer cell lines at a concentration of 20 μM. Compound **10b**, designed with a 4-F substituent, displayed comparable antiproliferative activity to compound **5** against HCT116 and PC9 but exhibited a 3-fold activity loss against A549 cells, despite the known pharmacological benefits of fluorine atoms in drug design^23^. Compound **10c**, with an electron-withdrawing 4-NO_2_ group, only exhibited similar antiproliferative activity to compound **5** against HCT116 (IC_50_ = 5.66 μM), but had no effect on PC9 and A549 cells at 20 μM. Compound **10d**, derived from a naturally-occurring cinnamic acid and containing *α*, *β*-unsaturated carbonyl groups conducive to binding to hydrophobic pockets in drug modification scenarios^24^, strikingly showed no inhibitory activity on three cancer cells. Substituting the phenyl ring with heteroaromatic rings, such as pyridine (**10e**), pyridazine (**10f**), thiophene (**10g**) and indole (**10h**) rings, generally maintained potent inhibitory activity against HCT116 and PC9, with IC_50_ ranging from 3.07 to 12.75 μM. However, against A549, compound **10f** showed a smaller improvement with an IC_50_ value of 3.11 μM, while compounds **10e**, **10g**, and **10h** showed varying activity loss. Non-aromatic esters were then introduced with a morpholine ring (**10i**), a cyclopropyl group (**10j**), and an *n*-heptyl group (**10k**), but no obvious enhancement in effect was observed. Further exploration of phenolic hydroxyl group modification involved synthesising ether derivatives **11a–b**. Benzyl ether (**11a)** slightly decreased the inhibitory activity against the three cancer cells compared to the parent compound **5**, while propenyl ether (**11b)** showed no activity against three cancer cells at 20 μM.

Compound **12**, with its five-fused-ring structure, exhibited compromised activity due to poor solubility (maximum measured: 5 μM). However, compound **13**, featuring an oxopropyl group at the *ortho*-position, surprisingly displayed superior antiproliferative activity against all three cancer cell lines. It demonstrated a 4.5-fold increase in effect on HCT116 (IC_50_ = 1.18 μM), a 12-fold increase on PC9 (IC_50_ = 0.57 μM), and a 2.6-fold increase on A549 cell lines (IC_50_ = 2.25 μM) compared to the positive control, compound **5**. Additionally, its activity is more potent than several reported natural products derived from marine fungi. For instance, purpuride G from *Talaromyces* sp. ZZ1616 showed an IC_50_ of 2.1 μM against PC9 cells^25^, chlorotrinoreremophilane sesquiterpene from *Penicillium* sp. (PR19N-1) had an IC_50_ of 12.2 μM against A549 cells, and apochalasin V from *Aspergillus* sp. exhibited an IC_50_ of 39.2 μM against HCT116 cells^26^. Since *ortho*-substitution was preferred, compounds **14** and **15**, with two substituents at both the *ortho*-position and the free phenolic hydroxyl group site, respectively, were further crafted. Regrettably, both exhibited lower activity than compound **5**, showing no inhibitory activity at 20 μM against all three cancer cell lines. Together, compound **13** demonstrated the best potent and well-balanced antiproliferative activity against three cancer cells, prompting further investigation into its biological mechanisms.

**Figure 1.**
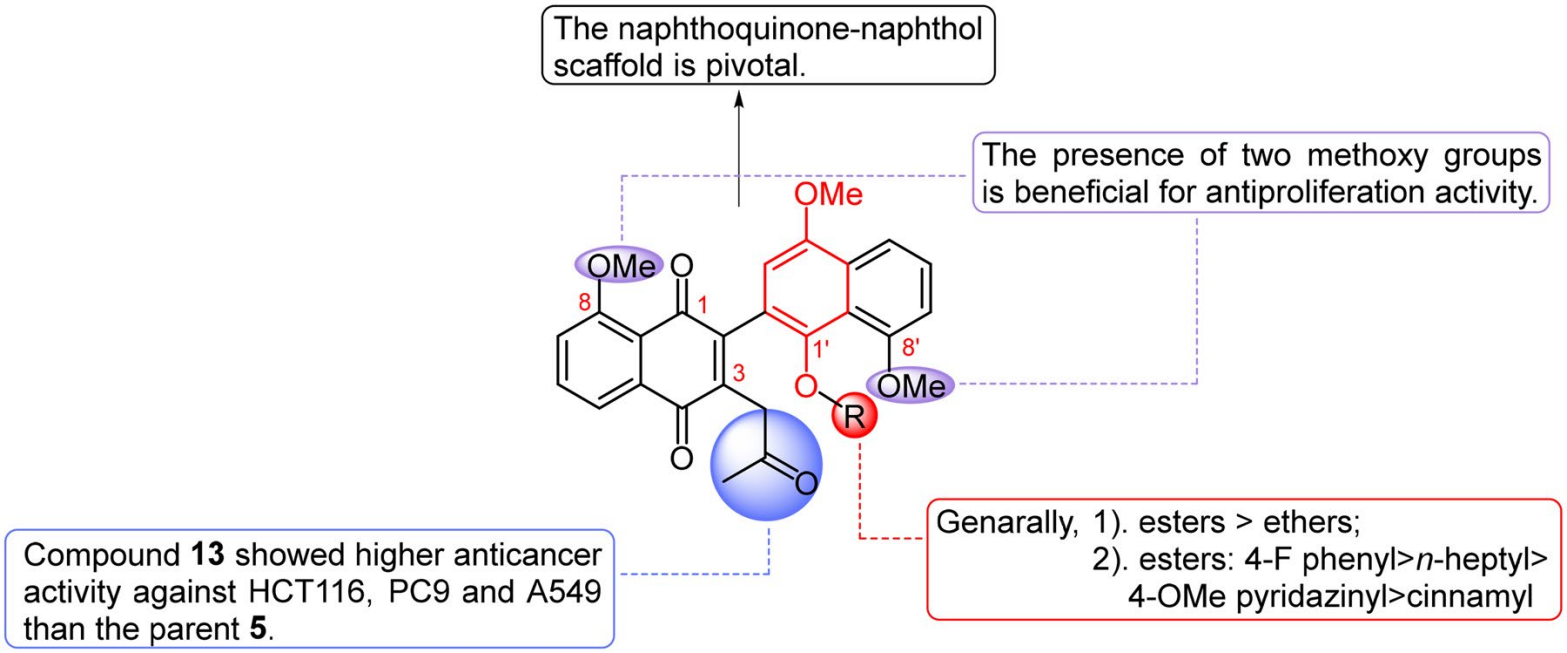
The summarised SAR of naphthoquinone-naphthol derivatives.

The structure-activity relationship (SAR) derived from the above discussion is depicted in Figure 1. The entire naphthoquinone-naphthol scaffold emerged as pivotal for antiproliferative activity, with the presence of two methoxy groups proving beneficial. Regarding derivations of the phenolic hydroxyl group, compounds such as 4-F benzoate ester, heterocyclic esters, aliphatic esters, and benzyl ether were found to be optimal, though only exhibiting activity comparable to the parent **5**. Notably, the *ortho*-substitution of the quinone group with an oxopropyl group (compound **13**) significantly enhanced the effect against all three cancer cell lines. However, combining *ortho*-substitution with phenolic hydroxyl group esterification or etherification weakened the effect considerably. In summary, compound **13**, featuring an oxopropyl group at the *ortho*-position of the quinone group, demonstrated the most potent antiproliferative activity against the three cancer cell lines (IC_50_ = 1.18 μM in HCT116 cells, 0.57 μM in PC9 cells, and 2.25 μM in A549 cells).

### Compound 13 suppressed the proliferation of both HCT116 and PC9 cells

**Figure 2.**
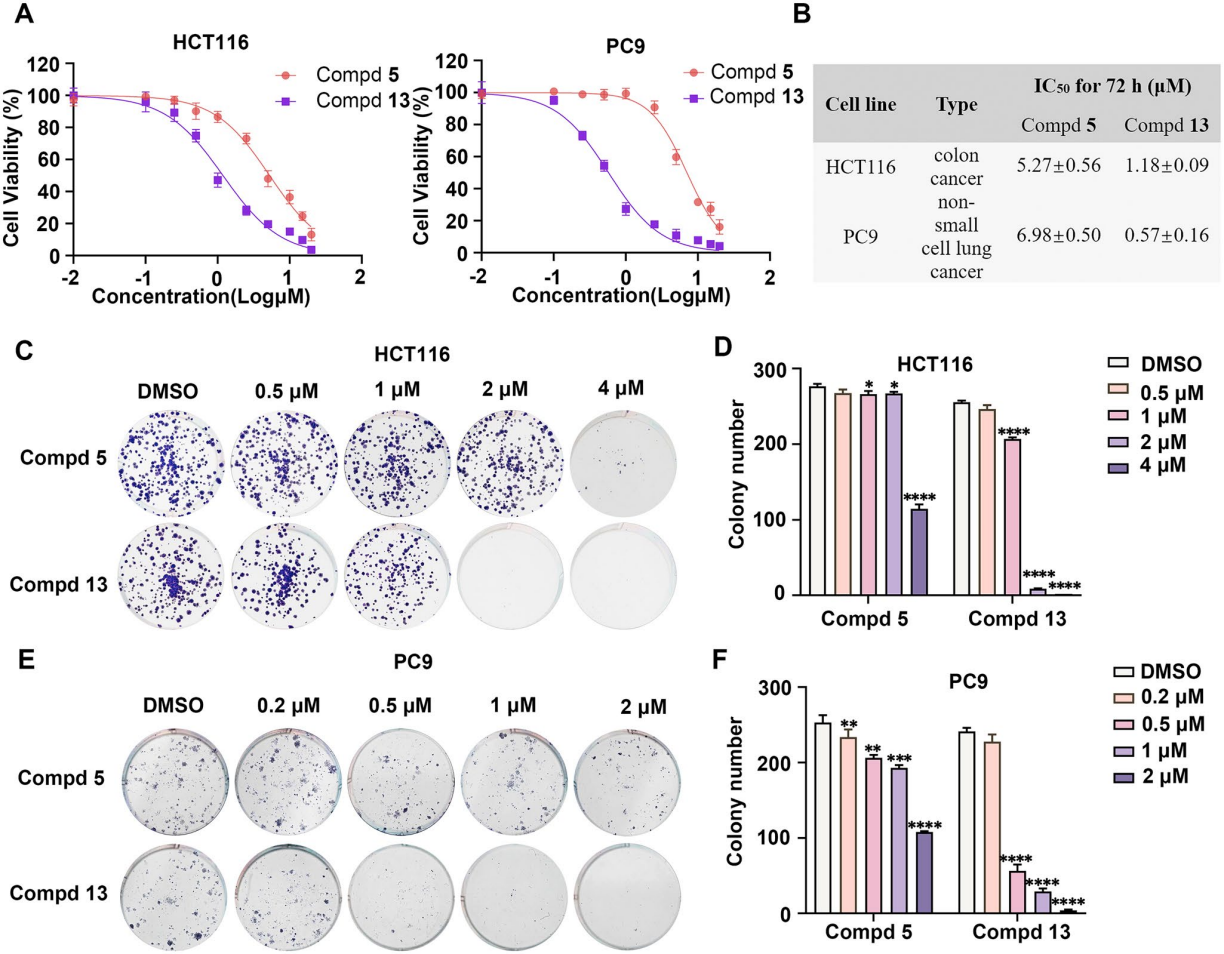
The antiproliferation effects of compound **5** and **13**. (A) HCT116 and PC9 were treated with the indicated concentrations of compound **5** and **13** (0.01, 0.1, 0.25, 0.5, 1, 2.5, 5, 10 and 20 μM) or DMSO for 72 h. Cell viability was evaluated using CCK-8 assay and shown as relative viability compared to the untreated control. Each test was performed in triplicate. (B) The IC_50_ values of compound **5** and **13** in HCT116 and PC9 cells were assessed after 72 h of incubation. (C) Compound **5** and **13** dose-dependently inhibited colony formation in HCT116 cells. Colony formation was assessed after treatment at concentrations of 0.5, 1, 2, and 4 μM for 14 days, and images of crystal violet-stained colonies were depicted. (D) The statistical result of (C). (E) Compound **5** and **13** dose-dependently inhibited colony formation in PC9 cells. Colony formation was assessed after treatment at concentrations of 0.2, 0.5, 1 and 2 μM for 14 d, and images of crystal violet-stained colonies were depicted. (F) The statistical result of (E). All data are shown as mean ± SD, *n* = 3, t test, *p < .05, **p < .01, ***p < .001, ****p < .0001.

*In vitro* antiproliferation experiments have demonstrated the efficacy of compound **13** in inhibiting the proliferation of both colon cancer and NSCLC lines (Figure 2A,B). To further elucidate its mechanism, colony formation assays were performed to evaluate the impact of compound **13** on HCT116 and PC9 cells. Compared to compound **5**, treatment with compound **13** at indicated concentrations significantly reduced colony formation rates in both HCT116 and PC9 cells after 14 days. Compound **13** exhibited a dose-dependent decrease in the colony-forming ability of these two cancer cell lines. Particularly noteworthy, treatment with 2 μM of compound **13** nearly completely suppressed colony formation in HCT116 cells, reducing both the number and size of the colonies (Figure 2C,D). Similarly, in PC9 cells, obvious inhibition of colony formation was observed at a concentration of 0.5 μM, with almost no colonies forming at 1 μM (Figure 2E,F).

### Compound13 inhibited the EGFR/PI3K/Akt signalling pathway in both HCT116 and PC9 cells

**Figure 3.**
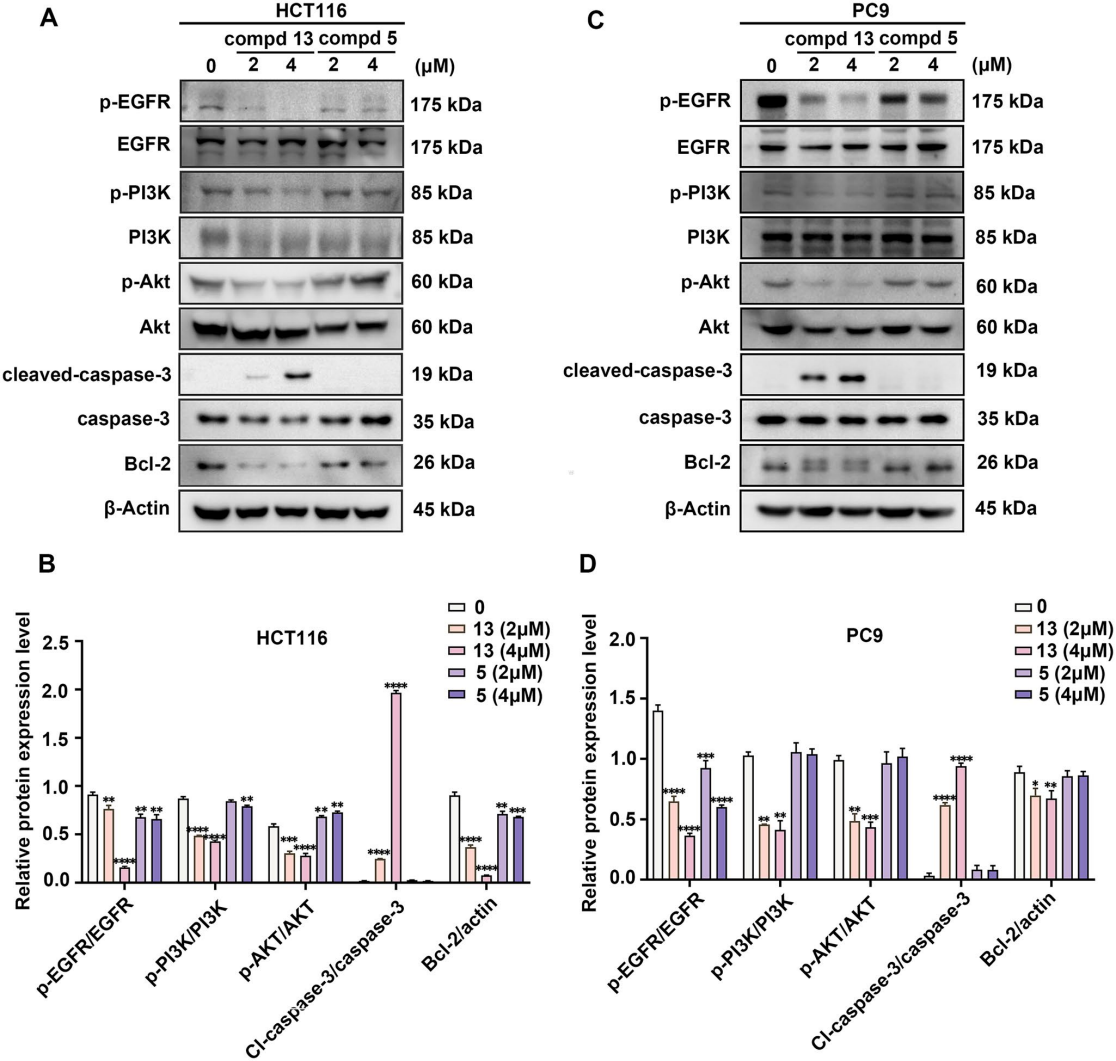
Western blot assay of compound **5** and **13**. (A) Western blot assay of EGFR phosphorylation (p-EGFR), PI3K phosphorylation (p-PI3K), Akt phosphorylation (p-Akt), caspase-3, cleaved-caspase-3, and Bcl-2 after treatment of HCT116 cells with 2 μM and 4 μM concentrations of compound **5** and **13** for 24 h. (B) The statistical result of (A). (C) Western blot assay of EGFR phosphorylation (p-EGFR), PI3K phosphorylation (p-PI3K), Akt phosphorylation (p-Akt), caspase-3, cleaved-caspase-3, and Bcl-2 after treatment of PC9 cells with 2 μM and 4 μM concentrations of compound **5** and **13** for 24 h. (D) The statistical result of (C). All data are shown as mean ± SD, *n* = 3, t test, *p < .05, **p < .01, ***p < .001, ****p < .0001.

The physiological function of the Epidermal Growth Factor Receptor (EGFR) is essential in various cellular processes and is often aberrantly activated in cancers, contributing to tumour occurrence and progression^27^. Its downstream PI3K/Akt signalling pathway plays a pivotal role in many cancers, controlling essential aspects such as cell survival, metastasis, and metabolism^28^. Inspired by the remarkable effect of compound **13** against HCT116 and PC9 cell lines, we investigated its effect on EGFR phosphorylation and its downstream signalling transduction in both cell lines using Western blotting analysis. As illustrated in Figure 3, both compounds **5** and **13** dose-dependently inhibited the phosphorylation of EGFR, PI3K, and Akt proteins in both HCT116 and PC9 cells. Remarkably, compound **13** exhibited a stronger inhibitory effect, surpassing that of compound **5** at comparable concentrations. Furthermore, we explored the apoptosis-inducing effects of compound **13** on HCT116 and PC9 cells. Compared to compound **5**, compound **13** increased the expression of cleaved-caspase-3 by activating caspase-3 and reduced the expression of the anti-apoptotic factor Bcl-2 in a dose-dependent manner, which demonstrated that compound **13** inhibited the survival of HCT116 and PC9 cells by inducing apoptosis. These results suggest that compound **13** emerged as a potent EGFR inhibitor, suppressing cell proliferation by inducing cell apoptosis through the EGFR/PI3K/Akt pathway.

## Conclusion

In summary, a series of novel naphthoquinone-naphthol derivatives, inspired by compound **5** of marine origin, were designed and synthesised with the aim of developing potential anticancer agents. Among them, the preferred compound **13** emerged as the most promising candidate, demonstrating potent and well-balanced antiproliferative activity against three human cancer cell lines (HCT116: IC_50_ = 1.18 μM; PC9: IC_50_ = 0.57 μM; A549: IC_50_ = 2.25 μM). Notably, compound **13** showed significant improvements of 4.5-fold, 12-fold, and 2.6-fold compared to the parent **5** across these cell lines, respectively. The SAR analysis highlighted the importance of *ortho*-position substitution of the quinone group for enhancing activity. Biological mechanistic studies revealed that compound **13** effectively inhibited colony formation of HCT116 and PC9 cancer cells at concentrations of 0.5 μM and 1 μM, respectively. Additionally, compound **13** induced the apoptosis of HCT116 and PC9 cells by significantly increasing the expression of cleaved-caspase-3 and decreasing the expression of Bcl-2. Mechanistically, compound **13** also downregulated the phosphorylation of EGFR, PI3K, and Akt proteins in HCT116 and PC9 cells in a dose-dependent manner. Further studies are needed to explore its pharmacokinetic properties, evaluate its *in vivo* anti-tumour activity, and assess its toxicology to develop it into an anti-tumour drug. Overall, the marine-derived compound **13** with its novel scaffold holds promise for the development of anticancer agents.

## Experimental

### General chemistry

Compounds **1** and **7** were purchased from Shanghai Macklin Biochemical Technology Co., Ltd. All other commercially available reagents and solvents were sourced from Energy Chemical (Shanghai, China) and Shanghai Macklin Biochemical Technology Co., Ltd., and were used without further purification. Reaction progress was monitored via thin layer chromatography (TLC), employing silica gel (300–400 mesh) for column chromatography. Spot visualisation was achieved using ultraviolet light or alkaline potassium permanganate. For spectroscopic analysis,^1^H NMR and ^13^C NMR spectra were acquired using a Bruker 400 MHz spectrometer, with CDCl_3_ and DMSO-*d*_6_ as solvents (chemical shifts reported in ppm). Tetramethylsilane (TMS) served as the internal standard, with CDCl_3_ and DMSO-*d*_6_ as references. High-resolution mass spectrometry (HRMS) was performed using an Orbitrap Exploris mass spectrometer (Thermo Scientific, USA) in the ESI mode. Known compounds underwent analysis using a Waters tandem quadrupole liquid chromatography-mass spectrometer. The melting points of all compounds were determined using an SGW X-4 micro melting point instrument without correction.

#### Synthesis of 1’-hydroxy-4’,8,8’-trimethoxy-[2,2’-binaphthalene]-1,4-dione (5)

To a solution of compound **4** (0.25 mmol, 1 equiv.) in CH_2_Cl_2_ (6 ml) was added *p*-chloranil (0.54 mmol, 2.16 equiv.) at room temperature. The mixture was vigorously stirred at room temperature for 3 d. After completion, the reaction solution was concentrated under vacuum, and the resulting residue was purified via silica gel column chromatography to give compound **5**. Black solid; yield: 84%.^1^H NMR (400 MHz, CDCl_3_)δ 9.42 (s, 1H, OH), 7.86 (d, *J =* 8.5 Hz, 1H, naphthol-1H), 7.76 (d, *J* = 7.6 Hz, 1H, naphthoquinyl-1H), 7.67 (t, *J =* 8.0 Hz, 1H, naphthoquinyl-1H), 7.38 (t, *J =* 8.1 Hz, 1H, naphthol-1H), 7.31 (d*, J =* 8.5 Hz, 1H, naphthoquinyl-1H), 7.04 (s, 1H, naphthoquinyl-1H), 6.86 (d, *J =* 7.8 Hz, 1H, naphthol-1H), 6.72 (s, 1H, naphthol-1H), 4.01 (d, *J =* 6.8 Hz, 6H, OCH_3_), 3.95 (s, 3H, OCH_3_). ^13^C NMR (100 MHz, CDCl_3_) δ 185.54, 183.45, 159.75, 156.56, 151.10, 147.92, 146.44, 134.59, 134.56, 134.49, 129.03, 126.50, 121.23, 118.76, 117.89, 116.08, 115.45, 114.99, 107.17, 105.71, 56.63, 56.26, 56.06. MS (ESI): m/z 391.1 [M + H]^+^.

#### Synthesis of 8,8’-dimethoxy-[2,2’-binaphthalene]-1,1’,4,4’-tetraone (6)^29^

Compound **5** (0.13 mmol, 1 equiv.) was dissolved in 10 ml of acetonitrile in an ice bath. A solution of ammonium cerium nitrate (2.5 equiv.) was prepared by dissolving it in 0.6 ml of water, then slowly added to the reaction mixture. The reaction stirred for 45 min at 0 °C. After completion, 10 ml of water was added to the reaction mixture, followed by extraction with CH_2_Cl_2_ (10 ml × 3). The organic phase underwent washing with brine, drying with Na_2_SO_4_, and concentration under vacuum. The resulting residue was purified via silica gel column chromatography to give compound **6**. Yellow solid; yield: 85%. ^1^H NMR (400 MHz, CDCl_3_) δ 7.72 (m, 4H, naphthoquinyl-4H), 7.31 (d, *J =* 8.2 Hz, 2H, naphthoquinyl-2H), 6.95 (s, 2H, naphthoquinyl-2H), 3.99 (s, 6H, OCH_3_). MS (ESI): m/z 397.0 [M + Na]^+^.

#### Synthesis of 1’-hydroxy-4’-methoxy-[2,2’-binaphthalene]-1,4-dione (9)

To a solution of compound **8** (0.15 mmol, 1 equiv.) in CH_2_Cl_2_ (15 ml) was added SnO_2_ (5 g). The mixture was vigorously stirred at room temperature for 24 h. After filtration, the residue was washed with CH_2_Cl_2_. The filtrate was then concentrated under vacuum, and the resulting residue was purified via silica gel column chromatography to give compound **9**. Black solid; yield: 69%. ^1^H NMR (400 MHz, CDCl_3_) δ 8.49 (s, 1H, OH), 8.45**–**8.36 (m, 1H, naphthol-1H), 8.29**–**8.10 (m, 3H, naphthol-1H, naphthoquinyl-2H), 7.89**–**7.76 (m, 2H, naphthoquinyl-2H), 7.57**–**7.59 (m, 2H, naphthol-2H), 7.14 (s, 1H, naphthoquinyl-1H), 6.56 (s, 1H, naphthol-1H), 3.99 (s, 3H, OCH_3_). MS (ESI): m/z 329.1[M-H]^–^.

#### Synthesis of ester derivatives 10a–10k

To a solution of different carboxylic acids or acyl chlorides (0.26 mmol, 2 equiv.) in dry CH_2_Cl_2_ (3 ml) at 0 °C was added HOAt (0.13 mmol, 1 equiv.), EDCI (0.325 mmol, 2.5 equiv.), 4-DMAP (0.25 mmol, 1 equiv.), and the mixture was stirred at room temperature for 15 min. Subsequently, compound **5** (0.13 mmol, 1 equiv.) was added to the reaction mixture, which was vigorously stirred at room temperature or reflux for 7–12 h. The reaction mixture was quenched by H_2_O (5 ml), and extracted with CH_2_Cl_2_ (5 ml × 3). The combined organic phase was washed with brine, dried with Na_2_SO_4_, and concentrated under vacuum. The resulting residue was then purified by silica gel column chromatography to obtain the pure target compounds **10a–10k**.

##### 4,8,8’-Trimethoxy-1’,4’-dioxo-1’,4’-dihydro-[2,2’-binaphthalen]-1-yl 4-ethoxybenzoate (10a)

Black solid; yield: 73%; m.p. 267.1 °C–267.5 °C. ^1^H NMR (400 MHz, CDCl_3_) δ 8.09**–**8.04 (m, 2H, phenyl-2H), 7.90 (d, *J =* 8.5 Hz, 1H, naphthol-1H), 7.69**–**7.60 (m, 2H, naphthoquinyl-2H), 7.41 (t, *J =* 8.1 Hz, 1H, naphthol-1H), 7.24 (d, *J =* 8.1 Hz, 1H, naphthoquinyl-1H), 7.04 (s, 1H, naphthoquinyl-1H), 6.86 (m, 3H, naphthol-1H, phenyl-2H), 6.76 (s, 1H, naphthol-1H), 4.07 (q, *J =* 7.0 Hz, 2H, CH_2_), 4.02 (s, 3H, OCH_3_), 3.96 (s, 3H, OCH_3_), 3.52 (s, 3H, OCH_3_), 1.43 (t, *J =* 7.0 Hz, 3H, CH_3_). ^13^C NMR (100 MHz, CDCl_3_) δ 184.98, 183.06, 165.40, 162.85, 159.55, 155.77, 152.75, 149.42, 137.93, 134.93, 134.68, 134.24, 132.35, 128.99, 126.81, 124.46, 122.16, 120.60, 119.65, 118.71, 117.79, 114.82, 113.92, 107.32, 105.31, 63.70, 56.42, 55.88, 55.67, 14.70. HRMS (ESI) calc’d for C_32_H_26_O_8_ [M + Na]^+^ 561.1525, found 561.1531.

##### 4,8,8’-Trimethoxy-1’,4’-dioxo-1’,4’-dihydro-[2,2’-binaphthalen]-1-yl 4-fluorobenzoate (10b)

Red solid; yield: 84%; m.p. 243.6 °C–243.9 °C. ^1^H NMR (400 MHz, CDCl_3_) δ 8.15 (dd, *J =* 8.6, 5.6 Hz, 2H, phenyl-2H), 7.91 (d, *J =* 9.4 Hz, 1H, naphthol-1H), 7.65 (m, 2H, naphthoquinyl-2H), 7.43 (t, *J =* 8.0 Hz, 1H, naphthol-1H), 7.25 (d, *J =* 8.0 Hz, 1H, naphthoquinyl-1H), 7.09 (t, *J =* 8.6 Hz, 2H, phenyl-2H), 7.03 (s, 1H, naphthoquinyl-1H), 6.86 (d, *J =* 7.8 Hz, 1H, naphthol-1H), 6.76 (s, 1H, naphthol-1H), 4.02 (s, 3H, OCH_3_), 3.95 (s, 3H, OCH_3_), 3.51 (s, 3H, OCH_3_). ^13^C NMR (100 MHz, CDCl_3_) δ 184.92, 182.95, 167.08, 164.61, 159.55, 155.59, 152.97, 149.34, 137.57, 134.98, 134.79, 134.22, 132.89, 132.80, 129.01, 126.90, 126.24, 124.42, 120.53, 119.41, 118.76, 117.83, 115.55, 115.34, 114.95, 107.42, 105.24, 56.40, 55.90, 55.66. HRMS (ESI) calc’d for C_30_H_21_FO_7_ [M + Na]^+^ 535.1169, found 535.1177.

##### 4,8,8’-Trimethoxy-1’,4’-dioxo-1’,4’-dihydro-[2,2’-binaphthalen]-1-yl 4-nitrobenzoate (10c)

Yellow solid; yield: 89%; m.p. 252.2 °C–252.7 °C. ^1^H NMR (400 MHz, CDCl_3_) δ 8.29 (q, *J =* 8.5 Hz, 4H, phenyl-4H), 7.93 (d, *J =* 8.5 Hz, 1H, naphthol-1H), 7.71–7.61 (m, 2H, naphthoquinyl-2H), 7.45 (t, *J =* 8.1 Hz, 1H, naphthol-1H), 7.27 (d, *J =* 8.1 Hz, 1H, naphthoquinyl-1H), 7.02 (s, 1H, naphthoquinyl-1H), 6.88 (d, *J =* 7.8 Hz, 1H, naphthol-1H), 6.76 (s, 1H, naphthol-1H), 4.00 (d, *J =* 24.2 Hz, 6H, OCH_3_), 3.51 (s, 3H, OCH_3_). ^13^C NMR (100 MHz, CDCl_3_) δ 184.79, 182.76, 163.70, 159.58, 155.30, 153.27, 150.61, 149.12, 137.10, 135.37, 135.11, 134.97, 134.15, 131.34, 128.99, 127.03, 124.38, 123.48, 120.36, 119.05, 118.84, 117.95, 115.14, 107.60, 105.18, 56.45, 55.95, 55.72. HRMS (ESI) calc’d for C_30_H_21_NO_9_ [M + Na]^+^ 562.1114, found 562.1118.

##### 4,8,8’-Trimethoxy-1’,4’-dioxo-1’,4’-dihydro-[2,2’-binaphthalen]-1-yl cinnamate (10d)

Red solid; yield: 84%; m.p. 242.3 °C–242.9 °C. ^1^H NMR (400 MHz, CDCl_3_) δ 7.91 (dd, *J =* 8.5, 1.0 Hz, 1H, naphthol-1H), 7.79 (d, *J =* 16.0 Hz, 1H, CH), 7.72 (dd, *J =* 7.6, 1.2 Hz, 1H, naphthoquinyl-1H), 7.64 (t, *J =* 8.0 Hz, 1H, naphthoquinyl-1H), 7.52 (m, 2H, phenyl-2H), 7.43 (t, *J =* 8.0 Hz, 1H, naphthol-1H), 7.40–7.36 (m, 3H, phenyl-3H), 7.24 (d, *J =* 8.0 Hz, 1H, naphthoquinyl-1H), 7.02 (s, 1H, naphthoquinyl-1H), 6.89 (dd, *J =* 7.9, 1.0 Hz, 1H, naphthol-1H), 6.74 (s, 1H, naphthol-1H), 6.58 (d, *J =* 16.0 Hz, 1H, CH), 4.01 (s, 3H, OCH_3_), 3.93 (s, 3H, OCH_3_), 3.78 (s, 3H, OCH_3_). ^13^C NMR (100 MHz, CDCl_3_) δ 185.00, 183.03, 165.78, 159.69, 155.73, 152.84, 149.29, 145.96, 137.48, 135.07, 134.77, 134.42, 134.32, 130.46, 128.95, 128.92, 128.18, 126.85, 124.43, 120.53, 119.55, 118.82, 117.90, 117.59, 114.90, 107.39, 105.28, 56.47, 55.96, 55.88. HRMS (ESI) calc’d for C_32_H_24_O_7_ [M + Na]^+^ 543.1420, found 543.1427.

##### 4,8,8’-Trimethoxy-1’,4’-dioxo-1’,4’-dihydro-[2,2’-binaphthalen]-1-yl isonicotinate (10e)

Brown solid; yield: 36%; m.p. 251.9 °C–252.3 °C. ^1^H NMR (400 MHz, CDCl_3_) δ 8.79 (s, 2H, phenyl-2H), 7.91–7.97 (m, 3H, naphthol-1H, phenyl-2H), 7.71–7.60 (m, 2H, naphthoquinyl-2H), 7.44 (t, *J =* 8.1 Hz, 1H, naphthol-1H), 7.25 (d, *J =* 8.1 Hz, 1H, naphthoquinyl-1H), 7.01 (s, 1H, naphthoquinyl-1H), 6.87 (d, *J =* 7.8 Hz, 1H, naphthol-1H), 6.76 (s, 1H, naphthol-1H), 4.03 (s, 3H, OCH_3_), 3.94 (s, 3H, OCH_3_), 3.52 (s, 3H, OCH_3_). ^13^C NMR (100 MHz, CDCl_3_) δ 184.81, 182.80, 164.06, 159.52, 155.33, 153.28, 150.54, 149.24, 137.21, 137.10, 135.05, 134.92, 134.16, 129.01, 127.04, 124.33, 123.42, 120.44, 119.06, 118.81, 117.87, 115.10, 107.59, 105.14, 56.35, 55.94, 55.70. HRMS (ESI) calc’d for C_29_H_21_NO_7_ [M + Na]^+^ 518.1216, found 518.1224.

##### 4,8,8’-Trimethoxy-1’,4’-dioxo-1’,4’-dihydro-[2,2’-binaphthalen]-1-yl 6-methoxypyridazine-3-carboxylate (10f)

Brown solid; yield: 89%; m.p. 152.9 °C–153.4 °C. ^1^H NMR (400 MHz, CDCl_3_) δ 8.17 (d, *J =* 9.1 Hz, 1H, pyrazinyl-1H), 7.93 (dd, *J =* 8.5, 1.0 Hz, 1H, naphthol-1H), 7.71–7.61 (m, 2H, naphthoquinyl-2H), 7.44 (t, *J =* 8.1 Hz, 1H, naphthol-1H), 7.25 (d, *J =* 8.1 Hz, 1H, naphthoquinyl-1H), 7.06–7.00 (m, 2H, naphthoquinyl-1H, pyrazinyl-1H), 6.88 (d, *J =* 8.1 Hz, 1H, naphthol-1H), 6.77 (s, 1H, naphthol-1H), 4.21 (s, 3H, OCH_3_), 4.02 (s, 3H, OCH_3_), 3.93 (s, 3H, OCH_3_), 3.56 (s, 3H, OCH_3_). ^13^C NMR (100 MHz, CDCl_3_) δ 184.88, 182.86, 165.92, 162.55, 159.66, 155.44, 153.03, 148.66, 147.66, 137.23, 135.26, 134.81, 134.22, 130.77, 128.95, 126.98, 124.34, 120.49, 119.17, 118.80, 117.96, 116.68, 115.09, 107.73, 105.48, 56.44, 55.97, 55.93, 55.53. HRMS (ESI) calc’d for C_29_H_22_N_2_O_8_ [M + Na]^+^ 549.1274, found 549.1281.

##### 4,8,8’-Trimethoxy-1’,4’-dioxo-1’,4’-dihydro-[2,2’-binaphthalen]-1-yl thiophene-2-carboxylate (10g)

Orange solid; yield: 71%; m.p. 268.1 °C–268.4 °C. ^1^H NMR (400 MHz, CDCl_3_) δ 7.93–7.87 (m, 2H, naphthol-1H, thienyl-1H), 7.70 (d, *J =* 7.6 Hz, 1H, naphthoquinyl-1H), 7.64 (t, *J =* 7.9 Hz, 1H, naphthoquinyl-1H), 7.56 (dd, *J =* 5.0, 1.3 Hz, 1H, thienyl-1H), 7.42 (t, *J =* 8.2 Hz, 1H, naphthol-1H), 7.27 (d, *J =* 8.2 Hz, 1H, naphthoquinyl-1H), 7.09 (dd, *J =* 5.0, 3.7 Hz, 1H, thienyl-1H), 7.05 (s, 1H, naphthoquinyl-1H), 6.87 (d, *J =* 7.8 Hz, 1H, naphthol-1H), 6.75 (s, 1H, naphthol-1H), 4.01 (s, 3H, OCH_3_), 3.95 (s, 3H, OCH_3_), 3.58 (s, 3H, OCH_3_). ^13^C NMR (100 MHz, CDCl_3_) δ 184.98, 182.91, 160.97, 159.67, 155.67, 152.89, 148.98, 137.29, 135.13, 134.73, 134.27, 133.40, 132.90, 128.92, 127.64, 126.90, 124.52, 120.56, 119.54, 118.75, 117.87, 114.84, 107.42, 105.35, 56.39, 55.90, 55.76. HRMS (ESI) calc’d for C_28_H_20_O_7_S [M + Na]^+^ 523.0827, found 523.0833.

##### 4,8,8’-Trimethoxy-1’,4’-dioxo-1’,4’-dihydro-[2,2’-binaphthalen]-1-yl 1-methyl-1H-indole-3-carboxylate (10h)

Black solid; yield: 67%; m.p. 297.8 °C–298.0 °C. ^1^H NMR (400 MHz, CDCl_3_) δ 8.18 (d, *J =* 7.7 Hz, 1H, indolyl-1H), 7.93–7.86 (m, 2H, naphthol-1H, indolyl-1H), 7.64 (d, *J =* 7.5 Hz, 1H, naphthoquinyl-1H), 7.58 (t, *J =* 8.0 Hz, 1H, naphthoquinyl-1H), 7.41 (t, *J =* 8.1 Hz, 1H, naphthol-1H), 7.34–7.27 (m, 2H, naphthoquinyl-1H, indolyl-1H), 7.20–7.23 (m, 2H, indolyl-2H), 7.09 (s, 1H, naphthoquinyl-1H), 6.85 (d, *J =* 7.7 Hz, 1H, naphthol-1H), 6.77 (s, 1H, naphthol-1H), 4.02 (s, 3H, OCH_3_), 3.89 (s, 3H, OCH_3_), 3.81 (s, 3H, OCH_3_), 3.51 (s, 3H, NCH_3_). ^13^C NMR (100 MHz, CDCl_3_) δ 185.03, 183.05, 163.56, 159.59, 156.10, 152.60, 149.52, 137.86, 137.14, 135.98, 135.07, 134.57, 134.30, 128.98, 126.84, 126.74, 124.67, 122.71, 122.16, 121.91, 120.67, 120.18, 118.80, 117.91, 114.77, 109.54, 107.45, 106.71, 105.45, 56.44, 55.91, 55.89. HRMS (ESI) calc’d for C_33_H_25_NO_7_ [M + Na]^+^ 570.1529, found 570.1536.

##### 4,8,8’-Trimethoxy-1’,4’-dioxo-1’,4’-dihydro-[2,2’-binaphthalen]-1-yl morpholine-4-carboxylate (10i)

Orange solid; yield: 98%; m.p. 253.8 °C–254.3 °C. ^1^H NMR (400 MHz, CDCl_3_) δ 7.88 (d, *J =* 8.4 Hz, 1H, naphthol-1H), 7.79 (d, *J =* 8.0 Hz, 1H, naphthoquinyl-1H), 7.72 (t, *J =* 8.0 Hz, 1H, naphthoquinyl-1H), 7.41 (t, *J =* 8.2 Hz, 1H, naphthol-1H), 7.34 (d, *J =* 8.2 Hz, 1H, naphthoquinyl-1H), 7.05 (s, 1H, naphthoquinyl-1H), 6.90 (d, *J =* 7.8 Hz, 1H, naphthol-1H), 6.72 (s, 1H, naphthol-1H), 3.99 (s, 6H, OCH_3_), 3.89 (s, 3H, OCH_3_), 3.60 (m, 8H, CH_2_). ^13^C NMR (100 MHz, CDCl_3_) δ 185.03, 182.95, 159.53, 155.91, 153.84, 152.71, 149.55, 137.94, 134.95, 134.93, 134.33, 129.01, 126.77, 124.47, 120.54, 120.03, 118.90, 117.90, 114.92, 107.46, 105.21, 66.80, 66.64, 56.42, 56.00, 55.87, 44.74, 44.05. HRMS (ESI) calc’d for C_28_H_25_NO_8_ [M + Na]^+^ 526.1478, found 526.1484.

##### 4,8,8’-Trimethoxy-1’,4’-dioxo-1’,4’-dihydro-[2,2’-binaphthalen]-1-yl cyclopropanecarboxylate (10j)

Red solid; yield: 89%; m.p. 80.1 °C–80.7 °C. ^1^H NMR (400 MHz, CDCl_3_) δ 7.87 (d, *J =* 8.5 Hz, 1H, naphthol-1H), 7.79 (d, *J =* 7.6 Hz, 1H, naphthoquinyl-1H), 7.72 (t, *J =* 8.0 Hz, 1H, naphthoquinyl-1H), 7.41 (t, *J =* 8.1 Hz, 1H, naphthol-1H), 7.34 (d, *J =* 8.3 Hz, 1H, naphthoquinyl-1H), 6.96 (s, 1H, naphthoquinyl-1H), 6.89 (d, *J =* 7.8 Hz, 1H, naphthol-1H), 6.70 (s, 1H, naphthol-1H), 3.99 (d, *J =* 9.7 Hz, 6H, OCH_3_), 3.90 (s, 3H, OCH_3_), 1.76 (m, 1H, CH), 1.07 (m, 2H, CH_2_), 0.89 (m, 2H, CH_2_). ^13^C NMR (100 MHz, CDCl_3_) δ 184.94, 182.79, 172.58, 159.79, 155.63, 152.77, 149.41, 137.40, 135.06, 134.88, 134.26, 128.89, 126.74, 124.26, 120.41, 119.56, 118.82, 117.98, 114.95, 107.39, 105.23, 56.44, 55.99, 55.86, 34.27, 31.40, 28.96, 24.73, 22.33, 14.04. HRMS (ESI) calc’d for C_31_H_32_O_7_ [M + Na]^+^ 481.1263, found 481.1269.

##### 4,8,8’-Trimethoxy-1’,4’-dioxo-1’,4’-dihydro-[2,2’-binaphthalen]-1-yl octanoate (10k)

Brown solid; yield: 84%; m.p. 205.2 °C–205.6 °C. ^1^H NMR (400 MHz, CDCl_3_) δ 7.89 (d, *J =* 8.5 Hz, 1H, naphthol-1H), 7.77 (d, *J =* 7.6 Hz, 1H, naphthoquinyl-1H), 7.71 (t, *J =* 8.0 Hz, 1H, naphthoquinyl-1H), 7.42 (t, *J =* 8.1 Hz, 1H, naphthol-1H), 7.33 (d, *J =* 8.3 Hz, 1H, naphthoquinyl-1H), 6.98 (s, 1H, naphthoquinyl-1H), 6.89 (d, *J =* 7.8 Hz, 1H, naphthol-1H), 6.70 (s, 1H, naphthol-1H), 3.99 (d, *J =* 7.6 Hz, 6H, OCH_3_), 3.86 (s, 3H, OCH_3_), 2.44 (t, *J =* 7.6 Hz, 2H, CH_2_), 1.54 (q, *J =* 7.6 Hz, 2H, CH_2_), 1.34–1.03 (m, 8H, CH_2_), 0.78 (t, *J =* 6.9 Hz, 3H, CH_3_). ^13^C NMR (100 MHz, CDCl_3_) δ 185.04, 182.96, 173.62, 159.81, 155.72, 152.62, 149.00, 137.44, 135.08, 134.88, 134.31, 128.86, 126.77, 124.25, 120.45, 119.62, 118.82, 118.00, 114.83, 107.28, 105.36, 56.47, 55.85, 55.71, 29.72, 13.01, 8.31. HRMS (ESI) calc’d for C_27_H_22_O_7_ [M + Na]^+^ 539.2046, found 539.2042.

#### Synthesis of ether derivatives 11a–11b

A mixture of compound **5** (0.13 mmol, 1 equiv.), K_2_CO_3_ (1.3 mmol, 10 equiv.) and acetone (3 ml) were stirred at room temperature for 15 min. 3-bromopropylene or benzyl bromide (0.65 mmol, 5 equiv.) was introduced, and the mixture vigorously stirred at 45 °C for 10–14 h. After evaporating the acetone under reduced pressure, 5 ml of water was added, and the solution was extracted with CH_2_Cl_2_ (5 ml × 3). The organic phase was washed with brine, dried with Na_2_SO_4_, and concentrated under a vacuum. The residue was purified via silica gel column chromatography to obtain the pure target compounds **11a–11b**.

##### 1’-(Benzyloxy)-4’,8,8’-trimethoxy-[2,2’-binaphthalene]-1,4-dione (11a)

Brown solid; yield: 75%; m.p. 223.2–223.9 °C. ^1^H NMR (400 MHz, CDCl_3_) δ 7.92 (d, *J =* 8.4 Hz, 1H, naphthol-1H), 7.71 (d, *J =* 7.7 Hz, 1H, naphthoquinyl-1H), 7.66 (t, *J =* 7.9 Hz, 1H, naphthoquinyl-1H), 7.45 (t, *J =* 8.1 Hz, 1H, naphthol-1H), 7.25 (d, *J =* 8.1 Hz, 1H, naphthoquinyl-1H), 7.11–7.14 (m, 2H, phenyl-2H), 7.07–7.03 (m, 3H, phenyl-3H), 6.95 (d, *J =* 7.8 Hz, 1H, naphthol-1H), 6.90 (s, 1H, naphthoquinyl-1H), 6.68 (s, 1H, naphthol-1H), 4.80 (s, 2H, CH_2_), 3.99 (s, 3H, OCH_3_), 3.92 (d, *J =* 4.7 Hz, 6H, OCH_3_). ^13^C NMR (100 MHz, CDCl_3_) δ 185.16, 183.62, 159.43, 156.33, 151.31, 150.62, 146.69, 137.63, 134.73, 134.41, 134.25, 129.65, 128.18, 128.00, 127.52, 126.69, 125.18, 121.12, 120.54, 118.50, 117.67, 114.91, 107.13, 105.75, 56.36, 55.98, 55.84. HRMS (ESI) calc’d for C_30_H_24_O_6_ [M + Na]^+^ 503.1471, found 503.1477.

##### 1’-(Allyloxy)-4’,8,8’-trimethoxy-[2,2’-binaphthalene]-1,4-dione (11b)

Black solid; yield: 51%; m.p. 98.2 °C–98.5 °C. ^1^H NMR (400 MHz, CDCl_3_) δ 7.91 (dd, *J =* 17.9, 8.5 Hz, 1H, naphthol-1H), 7.78 (d, *J =* 7.6 Hz, 1H, naphthoquinyl-1H), 7.70 (t, *J =* 7.9 Hz, 1H, naphthoquinyl-1H), 7.43 (t, *J =* 8.1 Hz, 1H, naphthol-1H), 7.32 (d, *J =* 8.4 Hz, 1H, naphthoquinyl-1H), 7.07 (d, *J =* 4.2 Hz, 1H, naphthoquinyl-1H), 6.93 (d, *J =* 7.7 Hz, 1H, naphthol-1H), 6.68 (d, *J =* 4.5 Hz, 1H, naphthol-1H), 5.95–5.83 (m, 1H, CH), 5.16 (d, *J =* 15.9 Hz, 1H, CH), 5.00 (d, *J =* 10.4 Hz, 1H, CH), 4.26 (d, *J =* 5.6 Hz, 2H, CH_2_), 3.99 (d, *J =* 8.6 Hz, 6H, OCH_3_), 3.95 (s, 3H, OCH_3_). ^13^C NMR (100 MHz, CDCl_3_) δ 185.22, 183.98, 159.62, 156.27, 151.18, 150.34, 146.72, 135.21, 134.63, 134.36, 134.32, 129.44, 126.63, 124.89, 120.97, 120.62, 118.69, 117.84, 116.69, 114.86, 107.10, 105.83, 76.20, 56.45, 56.01, 55.84. HRMS (ESI) calc’d for C_26_H_22_O_6_ [M + Na]^+^ 453.1314, found 453.1320.

#### Synthesis of 1,5,8-trimethoxydinaphtho[1,2-b:2’,3’-d]furan-7,12-dione (12)

To a solution of compound **5** (0.13 mmol, 1 equiv.) in toluene (7 ml) was added *p*-chloranil (1.14 mmol, 1.1 equiv.), and the mixture was stirred at 120 °C for 2 d. Then, the mixture was concentrated under vacuum, and the residue was purified by silica gel column chromatography to yield compound **12**. Black solid; yield: 88%. ^1^H NMR (400 MHz, CDCl_3_) δ 7.97 (dd, *J =* 7.1, 2.0 Hz, 2H, naphthoquinyl-2H), 7.74–7.68 (m, 2H, phenyl-1H, naphthoquinyl-1H), 7.57 (t, *J =* 8.2 Hz, 1H, phenyl-1H), 7.35 (d, *J =* 8.2 Hz, 1H, phenyl-1H), 7.11 (d, *J =* 7.9 Hz, 1H, phenyl-1H), 4.17 (s, 3H, OCH_3_), 4.09 (d, *J =* 1.8 Hz, 6H, OCH_3_). MS (ESI): m/z 389.1[M + H]^+^.

#### Synthesis of 1’-hydroxy-4’,8,8’-trimethoxy-3–(2-oxopropyl)-[2,2’-binaphthalene]-1,4-dione (13)

To a solution of compound **5** (0.13 mmol, 1 equiv.) in acetone (9 ml) was added Cs_2_CO_3_ (0.65 mmol, 5 equiv.), KI (0.13 mmol, 1 equiv.), bromoacetic acid (0.65 mmol, 5 equiv.), and the mixture was stirred at room temperature for 17 h. After evaporating the acetone, 10 ml of water was added, and the solution was extracted with CH_2_Cl_2_ (10 ml × 3). The organic phase was washed with brine, dried with Na_2_SO_4_, and concentrated under a vacuum. The residue was purified via silica gel column chromatography to obtain compound **13**. Black solid; yield: 67%; m.p. 224.5 °C–225.2 °C. ^1^H NMR (400 MHz, DMSO-*d*_6_) δ 9.18 (s, 1H, OH), 7.83 (t, *J =* 8.0, 8.4 Hz,1H, naphthoquinyl-1H), 7.73 (d, *J =* 8.4 Hz, 1H, naphthol-1H), 7.66 (d, *J =* 7.6 Hz, 1H, naphthoquinyl-1H), 7.58 (d, *J =* 8.4 Hz, 1H, naphthoquinyl-1H), 7.46 (t, *J =* 8.0 Hz, 1H, naphthol-1H), 7.08 (d, *J =* 8.0 Hz, 1H, naphthol-1H), 6.63 (s, 1H, naphthol-1H), 4.01 (s, 3H, OCH_3_), 3.90 (s, 3H, OCH_3_), 3.81 (s, 3H, OCH_3_), 3.68 (d, *J =* 17.0 Hz, 1H, CH), 3.40 (d, *J =* 17.0 Hz, 1H, CH), 2.12 (s, 3H, CH_3_). ^13^C NMR (100 MHz, CDCl_3_) δ 204.85, 159.95, 156.42, 147.74, 144.94, 134.67, 126.09, 119.25, 118.01, 116.12, 107.11, 105.60, 77.16, 56.59, 56.26, 56.00, 43.39, 30.51. HRMS (ESI) calc’d for C_22_H_26_O_7_ [M + Na]^+^ 469.1263, found 469.1256.

#### Synthesis of 4,8,8’-trimethoxy-1’,4’-dioxo-3’-(2-oxopropyl)-1’,4’-dihydro-[2,2’-binaphthalen]-1-yl 1-methyl-1H-indole-3-carboxylate (14)

To a solution of 1-methyl-1H-indole-3-carboxylic acid (0.13 mmol, 1 equiv.) in dry CH_2_Cl_2_ (3 ml) at 0 °C was added HOAt (0.13 mmol, 1 equiv.), EDCI (0.275 mmol, 2.5 equiv.), 4-DMAP (0.11 mmol, 1 equiv.), and the mixture was stirred at room temperature for 15 min. Subsequently, compound **13** (0.11 mmol, 1 equiv.) was added to the reaction mixture, which was vigorously stirred at reflux for 72 h. The reaction mixture was quenched by H_2_O (5 ml), and extracted with CH_2_Cl_2_ (5 ml × 3). The combined organic phase was washed with brine, dried with Na_2_SO_4_, and concentrated under vacuum. The resulting residue was then purified via silica gel column chromatography to obtain compound **14**. Orange solid; yield: 40%; m.p. 290.2 °C–290.5 °C. ^1^H NMR (400 MHz, CDCl_3_) δ 8.12 (d, *J =* 7.8 Hz, 1H, indolyl-1H), 7.90 (d, *J =* 8.5 Hz, 1H, naphthol-1H), 7.82 (s, 1H, indolyl-1H), 7.64 (d, *J =* 7.6 Hz, 1H, naphthoquinyl-1H), 7.56 (t, *J =* 8.0 Hz, 1H, naphthoquinyl-1H), 7.40 (t, *J =* 8.2 Hz, 1H, naphthol-1H), 7.27 (d, *J =* 10.6 Hz, 2H, indolyl-2H), 7.18 − 7.21 (m, 2H, naphthoquinyl-1H, indolyl-1H), 6.85 (d, *J =* 8.0 Hz, 1H, naphthol-1H), 6.67 (s, 1H, naphthol-1H), 3.92 (s, 3H, OCH_3_), 3.87 (d, *J =* 17.7 Hz, 1H, CH), 3.77 (s, 3H, OCH_3_), 3.69 − 3.59 (m, 3H, OCH_3_), 3.51 (s, 3H, CH_3_), 3.46 (d, *J =* 9.4 Hz, 1H, CH), 2.19 (s, 3H, CH_3_). ^13^C NMR (100 MHz, CDCl_3_) δ 205.07, 184.75, 182.21, 159.68, 156.06, 152.81, 137.11, 135.97, 134.45, 134.06, 129.92, 129.60, 128.93, 128.68, 126.85, 126.42, 126.04, 125.80, 124.41, 122.68, 122.01, 121.85, 120.37, 120.23, 119.19, 117.94, 114.75, 109.59, 107.51, 106.55, 56.52, 55.99, 55.86, 43.23, 33.38, 30.42. HRMS (ESI) calc’d for C_36_H_29_NO_8_ [M + Na]^+^ 626.1791, found 626.1796.

#### Synthesis of 1’-(benzyloxy)-4’,8,8’-trimethoxy-3–(2-oxopropyl)-[2,2’-binaphthalene]-1,4-dione (15)

A mixture of compound **13** (0.11 mmol, 1 equiv.), K_2_CO_3_ (1.1 mmol, 10 equiv.) and acetone (4 ml) were stirred at room temperature for 15 min. Following this, benzyl bromide (0.55 mmol, 5 equiv.) was introduced, and the mixture vigorously stirred at 45 °C for 14 h. After evaporating the acetone, 5 ml of water was added, and the solution was extracted with CH_2_Cl_2_ (5 ml × 3). The organic phase was washed with brine, dried with Na_2_SO_4_, and concentrated under a vacuum. The residue was purified via silica gel column chromatography to obtain compound **15**. Orange solid; yield: 75%; m.p. 178.2 °C–178.7 °C. ^1^H NMR (400 MHz, CDCl_3_) δ 7.91 (d, *J =* 8.4 Hz, 1H, naphthol-1H), 7.74 (d, J = 7.6 Hz, 1H, naphthoquinyl-1H), 7.66 (t, *J =* 8.0 Hz, 1H, naphthoquinyl-1H), 7.44 (t, *J =* 8.0 Hz, 1H, naphthol-1H), 7.27 (d, *J =* 8.0 Hz, 1H, naphthoquinyl-1H), 7.15–7.19 (m, 2H, phenyl-2H), 7.11–7.06 (m, 2H, phenyl-2H), 6.95 (d, *J =* 8.0 Hz, 1H, naphthol-1H), 6.60 (s, 1H, naphthol-1H), 4.88 (d, *J =* 11.2 Hz, 1H, CH), 4.70 (d, *J =* 11.2 Hz, 1H, CH), 3.92 (d, *J =* 3.9 Hz, 6H, OCH_3_), 3.89 (s, 3H, OCH_3_), 3.83 (d, *J =* 17.0 Hz, 1H, CH), 3.31 (d, *J =* 17.0 Hz, 1H, CH), 2.13 (s, 3H, CH_3_). ^13^C NMR (100 MHz, CDCl_3_) δ 204.57, 184.90, 183.37, 159.57, 156.20, 151.20, 148.42, 145.70, 140.70, 138.08, 134.51, 134.05, 129.24, 128.02, 127.68, 127.30, 126.38, 124.35, 120.64, 120.54, 119.05, 117.80, 114.89, 107.05, 106.00, 56.39, 56.00, 55.83, 43.24, 30.48. HRMS (ESI) calc’d for C_33_H_28_O_7_ [M + Na]^+^ 559.1733, found 559.1740.

### Cell culture

The HCT116 and A549 cell lines were acquired from the American Type Culture Collection (ATCC, CCL-247, CCL-185), and the PC9 cell line was purchased from the European Collection of Authenticated Cell Cultures (ECACC, 90071810). All cell lines were regularly tested to confirm that they were free of *Mycoplasma*. HCT116 and PC9 cell lines were cultured in the RPMI-1640 medium (Gibco, USA), while A549 cell lines were cultured in DMEM medium (Gibco, USA). All culture media were supplemented with 10% FBS, 100 U/mL penicillin, and 100 μg/mL streptomycin, and maintained at 37 °C in a 5% CO_2_ atmosphere. Cells were used at low passages (3–5 passages) upon receipt from the suppliers^30^.

### In vitro anti-proliferative activity assay

*In vitro* anti-proliferative activity of the derivatives was evaluated using the CCK-8 assay against lung cancer cells (PC9 and A549) and colon cancer cells (HCT116). Cells were seeded in 96-well plates at a density of 5 × 10^3^ cells per well and allowed to adhere overnight. Compounds at various concentrations, with DMSO as the vehicle control, were then added. After 72 h of incubation, CCK-8 solution was introduced, followed by further incubation at 37 °C for 1 h. Absorbance at 450 nm was measured using a microplate reader (Spectramax Plus 384, Molecular Devices, Sunnyvale, CA, USA). IC_50_ values were determined using GraphPad Prism 9.0 software.

### Colony formation assay

To evaluate the long-term effects of compounds on HCT116 and PC9 cell growth, a colony formation assay was conducted. Cells were seeded at a density of 1000 cells per well in 6-well plates and allowed to adhere for 24 h. Following this, cells were treated with varying concentrations of compound **5** and **13**. The culture media were refreshed every other day, and cells were continuously incubated in fresh media for 14 d. After removing the media, cells were washed with cold PBS, fixed with 4% paraformaldehyde (PFA) at room temperature, stained with crystal violet for 15 min, and photographed using a camera. Macroscopic colonies in each well were then counted.

### Western blotting

Western blot analysis was performed as in our previous reports^31^. Cells were exposed to varying doses of compound **5** and **13** (0, 2, and 4 μM) for 24 h, followed by protein sample preparation using RIPA lysis buffer containing protease and phosphatase inhibitors. Equal amounts of total protein were then suspended in a sample buffer, boiled at 100 °C for 10 min, and separated by 10%-15% sodium dodecyl sulphate-polyacrylamide gel electrophoresis (SDS-PAGE). The proteins were transferred onto PVDF membranes (Merck Millipore, #IPFL00010, Germany). The membranes were blocked with 5% skim milk at room temperature for 1 h, followed by overnight incubation at 4 °C with specific primary antibodies. Subsequently, the membranes were washed three times for 5 min each with 1× TBST to remove unbound primary antibodies. They were then incubated at room temperature for 1 h with secondary antibodies conjugated to horseradish peroxidase (HRP) (1:10,000). After three washes with 1× TBST for 5 min each, the immunoblots were visualised using the Bio-Rad ChemiDoc XRS system and quantified using ImageJ software.

## Supplementary Material

Supplementary_data CLEAN.docx

Original Image for Figure 3A and 3C.tif

Original Image for Figure 2C and 2E.tif

## Data Availability

The data presented in the current study are available from the corresponding author upon reasonable request.
