# Supplementary material for: Design, synthesis and biological evaluation of marine naphthoquinone-naphthol derivatives as potential anticancer agents
Source: J Enzyme Inhib Med Chem. 2024 Oct 15;39(1):2412865. doi: 10.1080/14756366.2024.2412865 (PMC11486183; doi:10.1080/14756366.2024.2412865)

**Supplementary data**

**Design, synthesis and biological evaluation of marine naphthoquinone-naphthol derivatives as potential anticancer agents**

Yujuan Li ^a^, Luyou Yelv ^a^, Xiaoqiu Wu ^a^, Ning Liu ^a, b, c, d e^* and Yamin Zhu ^a, b, c, d e^*

^a^ Department of Chemistry, College of Food Science and Technology, Shanghai Ocean University, Shanghai, China; ^b^ Marine Biomedical Science and Technology Innovation Platform of Lingang Special Area, Shanghai, China; ^c^ Department of Marine Bio-Pharmacology, College of Food Science and Technology, Shanghai Ocean University, Shanghai, China; ^d^ International Research Center for Food & Health, Shanghai Ocean University, Shanghai, China; ^e^ Collaborative Innovation Center of Seafood Deep Processing, Ministry of Education, Shanghai Ocean University, Shanghai, China.

* E-mail address: zhuym@shou.edu.cn (Y. Zhu); nliu@shou.edu.cn (N. Liu).

**Table of Contents**

General procedure for the synthesis of **5**3-5

General procedure for the synthesis of **9**6-7

NMR spectra of Compound **5**8

NMR and HRMS spectra of Compound **10a**9-10

NMR and HRMS spectra of Compound **10b**11-12

NMR and HRMS spectra of Compound **10c**13-14

NMR and HRMS spectra of Compound **10d**15-16

NMR and HRMS spectra of Compound **10e**17-18

NMR and HRMS spectra of Compound **10f**19-20

NMR and HRMS spectra of Compound **10g**21-22

NMR and HRMS spectra of Compound **10h**23-24

NMR and HRMS spectra of Compound **10i**25-26

NMR and HRMS spectra of Compound **10j**27-28

NMR and HRMS spectra of Compound **10k**29-30

NMR and HRMS spectra of Compound **11a**31-32

NMR and HRMS spectra of Compound **11b**33-34

NMR and HRMS spectra of Compound **13**35-36

NMR and HRMS spectra of Compound **14**37-38

NMR and HRMS spectra of Compound **15**39-40

**1.General procedure for the synthesis of 5**

- 1. ***1,5-dimethoxynaphthalene (2)***

To a solution of naphthalene-1,5-diol (**1**) (0.62 mmol, 1 equiv.) in acetone (1 mL) was added dimethyl sulfate (6.2 mmol, 10 equiv.) and K_2_CO_3_ (6.2 mmol, 10 equiv.) at 0 ℃. After refluxing the mixture for 12 h, it was cooled to room temperature. The acetone solvent was evaporated under reduced pressure, followed by the addition of 10 mL of water. The mixture was extracted with ethyl acetate (50 mL × 3) and the combined organic phase was washed with brine (50 mL). The organic phase was dried with Na_2_SO_4_ and concentrated under reduced pressure to provide the crude product. The residue was purified by silica gel column chromatography to obtain the pure target compound **2**. Yellow solid; yield: 85%. ^1^H NMR (400 MHz, CDCl_3_) δ 7.83 (d, *J* = 8.8 Hz, 2H, phenyl-2H), 7.37 (t, *J* = 8.0 Hz, 2H, phenyl-2H), 6.85 (d, *J* = 7.6 Hz, 2H, phenyl-2H), 3.99 (s, 6H, OCH_3_).

***1.2. 4,8-dimethoxy-1-naphthaldehyde (3)***

A solution of POCl_3_ (2.73 mmol, 5.16 equiv.) in CHCl_3_ (1 mL) was mixed with DMF (2.756 mmol, 5.2 equiv.) at 0 ℃ and stirred at room temperature for 1 h. Following this, compound **2** (0.53 mmol, 1 equiv.) in CHCl_3_ (2.5 mL) was added to the mixture at the same temperature. The reaction mixture was heated to reflux for 10 h. During stirring, 2% NaOH solution was incrementally added to adjust the pH to neutral at 0 ºC. The resulting mixture was extracted with DCM (10 mL × 2), and the combined organic extracts were sequentially washed with 1 M HCl (10 mL), saturated aqueous NaHCO_3_ (10 mL), and brine (10 mL). After drying over Na_2_SO_4_ and concentrated under reduced pressure, the residue underwent purification via silica-gel flash column chromatography to afford compound **3**. Yellow solid; yield: 97%. ^1^H NMR (400 MHz, CDCl_3_) δ 11.05 (s, 1H, CHO), 8.09 (s, 1H, phenyl-1H), 7.95 (d, *J* = 8.4 Hz, 1H, phenyl-1H), 7.45 (t, *J* = 8.1 Hz, 1H, phenyl-1H), 7.04 (d, *J* = 7.7 Hz, 1H, phenyl-1H), 6.91 (s, 1H, phenyl-1H), 4.03 (d, *J* = 16.8 Hz, 6H, OCH_3_).

***1.3.* *4,8-dimethoxynaphthalen-1-ol (4)***

To a solution of compound **3** (0.46 mmol, 1 equiv.) in DCM (2.5 mL) was added *m*-CPBA (0.92 mmol, 2 equiv.) at room temperature. After stirring for 2 h, the reaction was quenched by adding 10% aqueous Na_2_S_2_O_3_. Extraction with DCM (7 mL × 3) followed, and the organic phase was then washed with saturated NaHCO_3_ (10 mL × 2) and brine (10 mL). After drying over Na_2_SO_4_, the filtrate was concentrated under vacuum. The resulting crude product was dissolved in THF (2.5 mL) and MeOH (1.25 mL), with K_2_CO_3_ (0.92 mmol, 2 equiv.) added to the mixture at 0 ℃. After stirring for 3 h, filtration was performed using a Brinell funnel, followed by washing with EtOAc. The filtrate was then poured into 1 M HCl and extracted with EtOAc (10 mL×3). The combined organic extracts were washed with water (10 mL × 2) and brine (10 mL), dried with Na_2_SO_4_, and concentration under vacuum. The resulting residue underwent purification via silica gel column chromatography, yielding the pure target compound **4**. White solid; yield: 61%. ^1^H NMR (400 MHz, CDCl_3_) δ 8.95 (s, 1H, OH), 7.85 (d, *J* = 8.6 Hz, 1H, phenyl-1H), 7.37 – 7.30 (m, 1H, phenyl-1H), 6.84 (dd, *J* = 7.8, 1.0 Hz, 1H, phenyl-1H), 6.78 (d, *J* = 2.2 Hz, 2H, phenyl-2H), 4.05 (s, 3H, OCH_3_), 3.93 (s, 3H, OCH_3_).

***1.4. 1'-hydroxy-4',8,8'-trimethoxy-[2,2'-binaphthalene]-1,4-dione (5)***

To a solution of compound **4** (0.25 mmol, 1 equiv.) in DCM (6 mL) was added *p*-chloranil (0.54 mmol, 2.16 equiv.) at room temperature. The mixture was vigorously stirred at room temperature for 3 days. Subsequently, the reaction solution was concentrated under vacuum, and the resulting residue underwent purification via silica gel column chromatography to yield the pure target compound **5**. Black solid; yield: 84%. ^1^H NMR (400 MHz, CDCl_3_) δ 9.42 (s, 1H, OH), 7.86 (d, *J* = 8.5 Hz, 1H, naphthol-1H), 7.76 (d, *J* = 7.6 Hz, 1H, naphthoquinyl-1H), 7.67 (t, *J* = 8.0 Hz, 1H, naphthoquinyl-1H), 7.38 (t, *J* = 8.1 Hz, 1H, naphthol-1H), 7.31 (d, *J* = 8.5 Hz, 1H, naphthoquinyl-1H), 7.04 (s, 1H, naphthoquinyl-1H), 6.86 (d, *J* = 7.8 Hz, 1H, naphthol-1H), 6.72 (s, 1H, naphthol-1H), 4.01 (d, *J* = 6.8 Hz, 6H, OCH_3_), 3.95 (s, 3H, OCH_3_). ^13^C NMR (100 MHz, CDCl_3_) δ 185.54, 183.45, 159.75, 156.56, 151.10, 147.92, 146.44, 134.59, 134.56, 134.49, 129.03, 126.50, 121.23, 118.76, 117.89, 116.08, 115.45, 114.99, 107.17, 105.71, 56.63, 56.26, 56.06. MS (ESI) m/z: 391.1 [M+H]^+^.

**2. General procedure for the synthesis of 9**

***2.1.* *(E)-4,4'-dimethoxy-1H,1'H-[2,2'-binaphthalenylidene]-1,1'-dione (8)***

To a solution of compound **7** (0.29 mmol, 1 equiv.) in DCM (7 mL) was added *p*-chloranil (0.34 mmol, 1.16 equiv.). The mixture was vigorously stirred at room temperature for 1 day. Following this, the reaction solution was concentrated under vacuum, and the resulting residue underwent purification via silica gel column chromatography to obtain the pure target compound **8**. Purple solid; yield: 90%. ^1^H NMR (400 MHz, CDCl_3_) δ 8.41 (s, 2H, phenyl-2H), 8.16 (d, *J* = 8.0 Hz, 2H, phenyl-2H), 7.79 (d, *J* = 8.0 Hz, 2H, phenyl-2H), 7.62 (t, *J* = 7.6 Hz, 2H, phenyl-2H), 7.48 (t, *J* = 7.6 Hz, 2H, CH), 4.08 (s, 6H, OCH_3_).

***2.2.* *1'-hydroxy-4'-methoxy-[2,2'-binaphthalene]-1,4-dione (9)***

To a solution of compound **8** (50 mg, 0.15 mmol, 1 equiv.) in DCM (15 mL) was added SnO_2_ (5 g). The mixture was vigorously stirred at room temperature for 1 day. The reaction solution was then filtered and the residue was rinsed with DCM. The filtrate was concentrated under vacuum, and the resulting residue underwent purification via silica gel column chromatography to obtain the pure target compound **9**. Black solid; yield: 69%. ^1^H NMR (400 MHz, CDCl_3_) δ 8.49 (s, 1H, OH), 8.45 – 8.36 (m, 1H, naphthol-1H), 8.29 – 8.10 (m, 3H, naphthol-1H, naphthoquinyl-2H), 7.89 – 7.76 (m, 2H, naphthoquinyl-2H), 7.57 – 7.59 (m, 2H, naphthol-2H), 7.14 (s, 1H, naphthoquinyl-1H), 6.56 (s, 1H, naphthol-1H), 3.99 (s, 3H, OCH_3_). MS (ESI): m/z 329.1[M-H]^–^.

Compound **5**：

^1^H NMR of **5** (400 MHz, CDCl_3_)


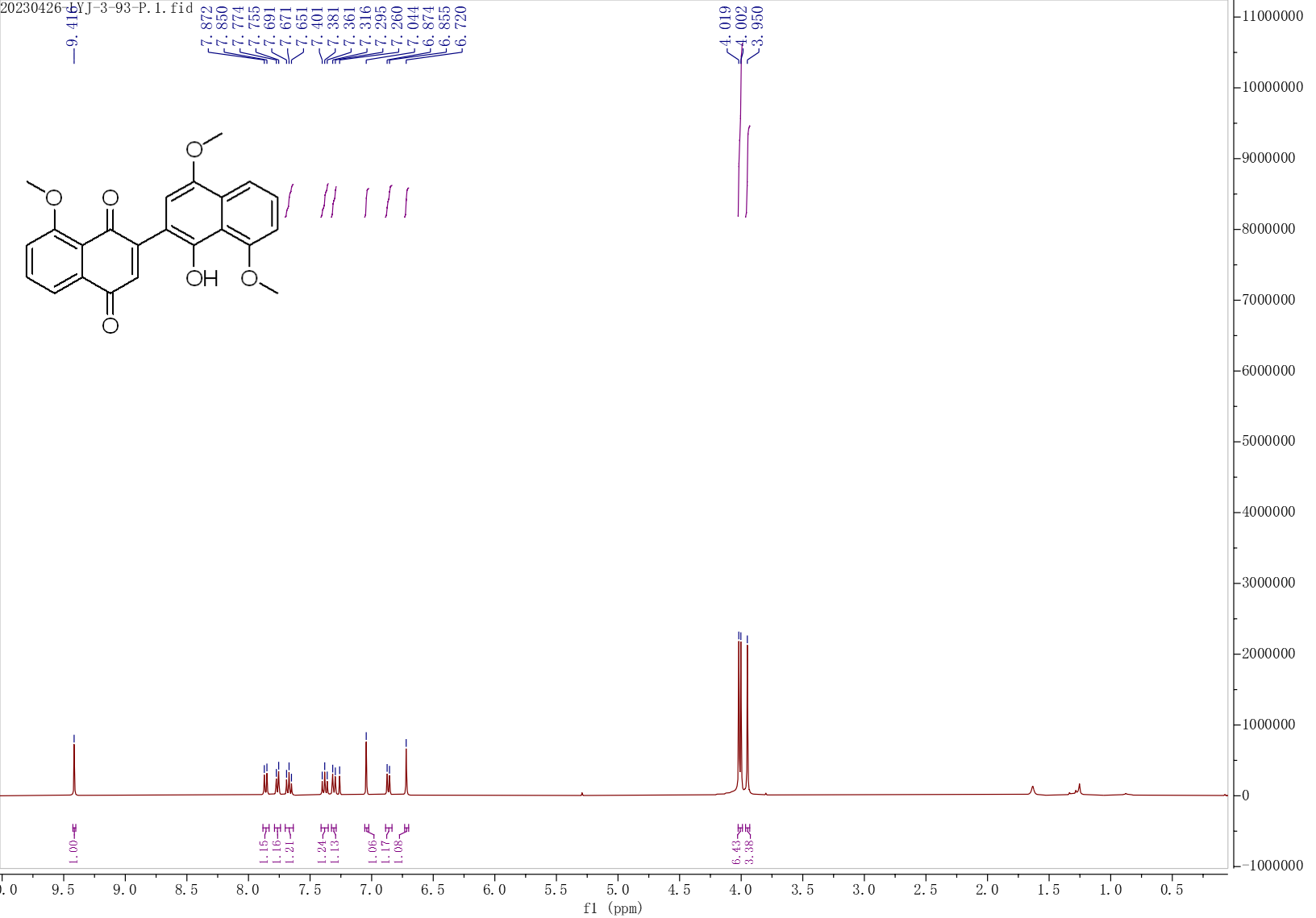


^13^C NMR of **5** (100 MHz, CDCl_3_)


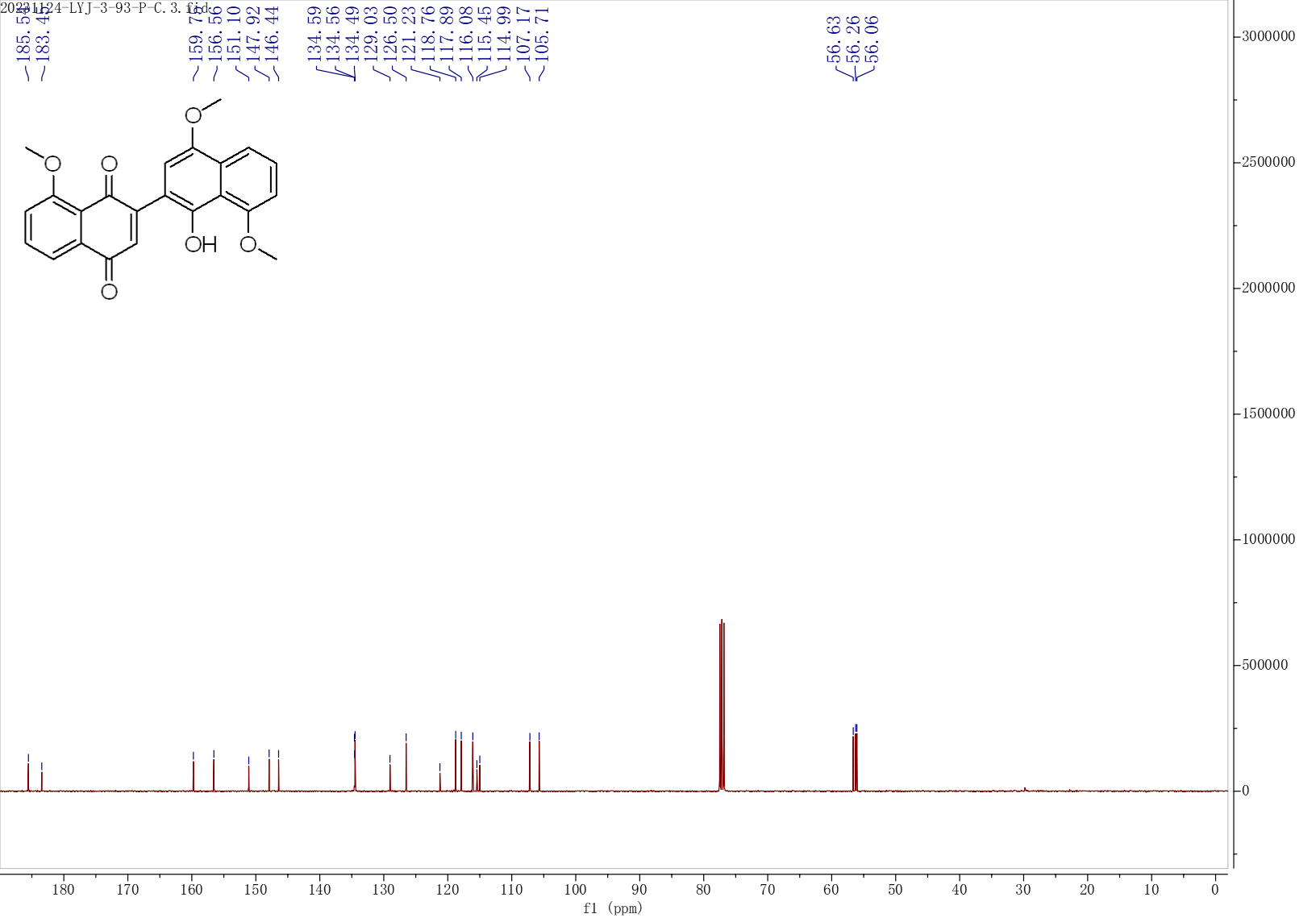


Compound **10a**:

^1^H NMR of **10a** (400 MHz, CDCl_3_)


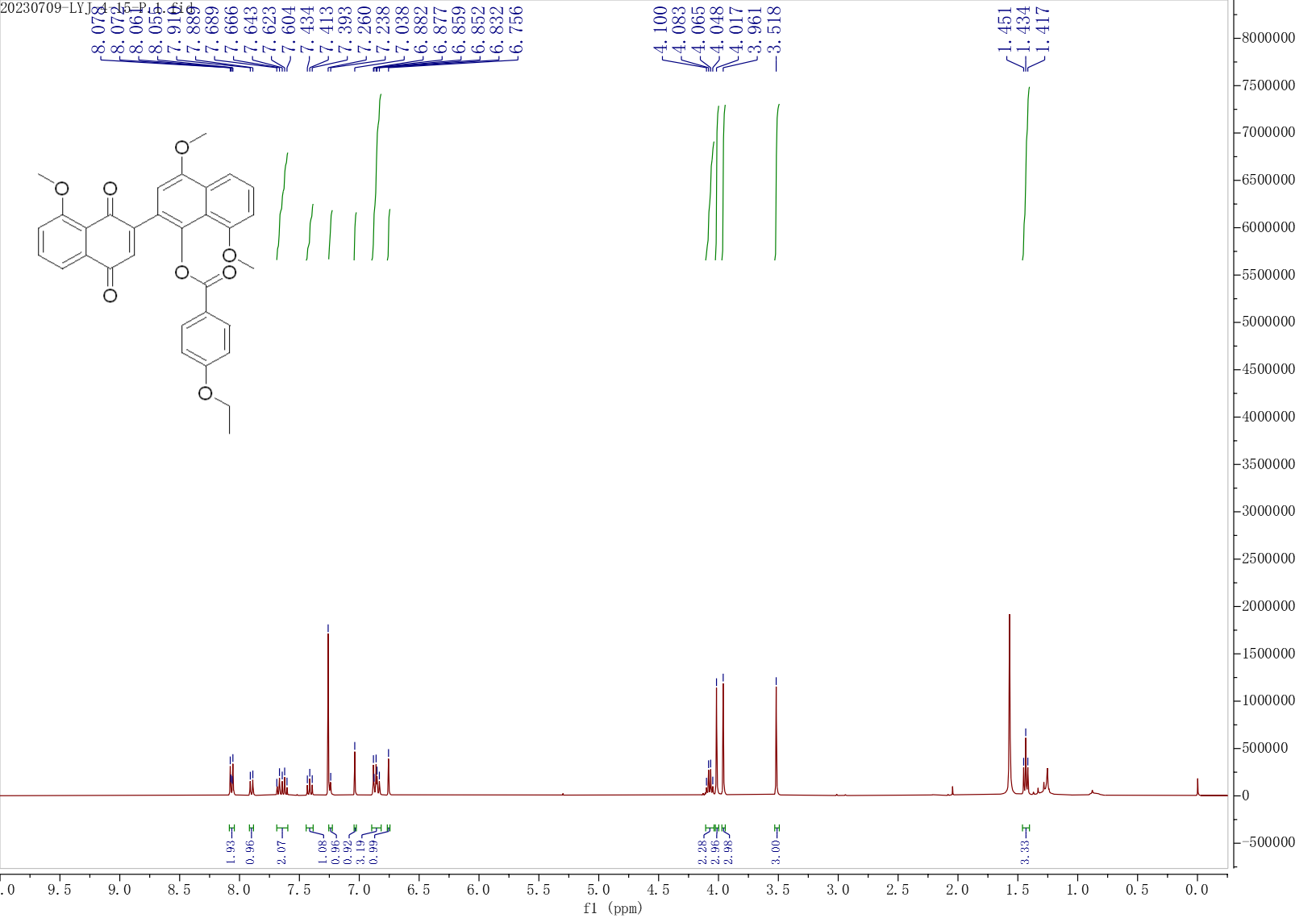


^13^C NMR of **10a** (100 MHz, CDCl_3_)


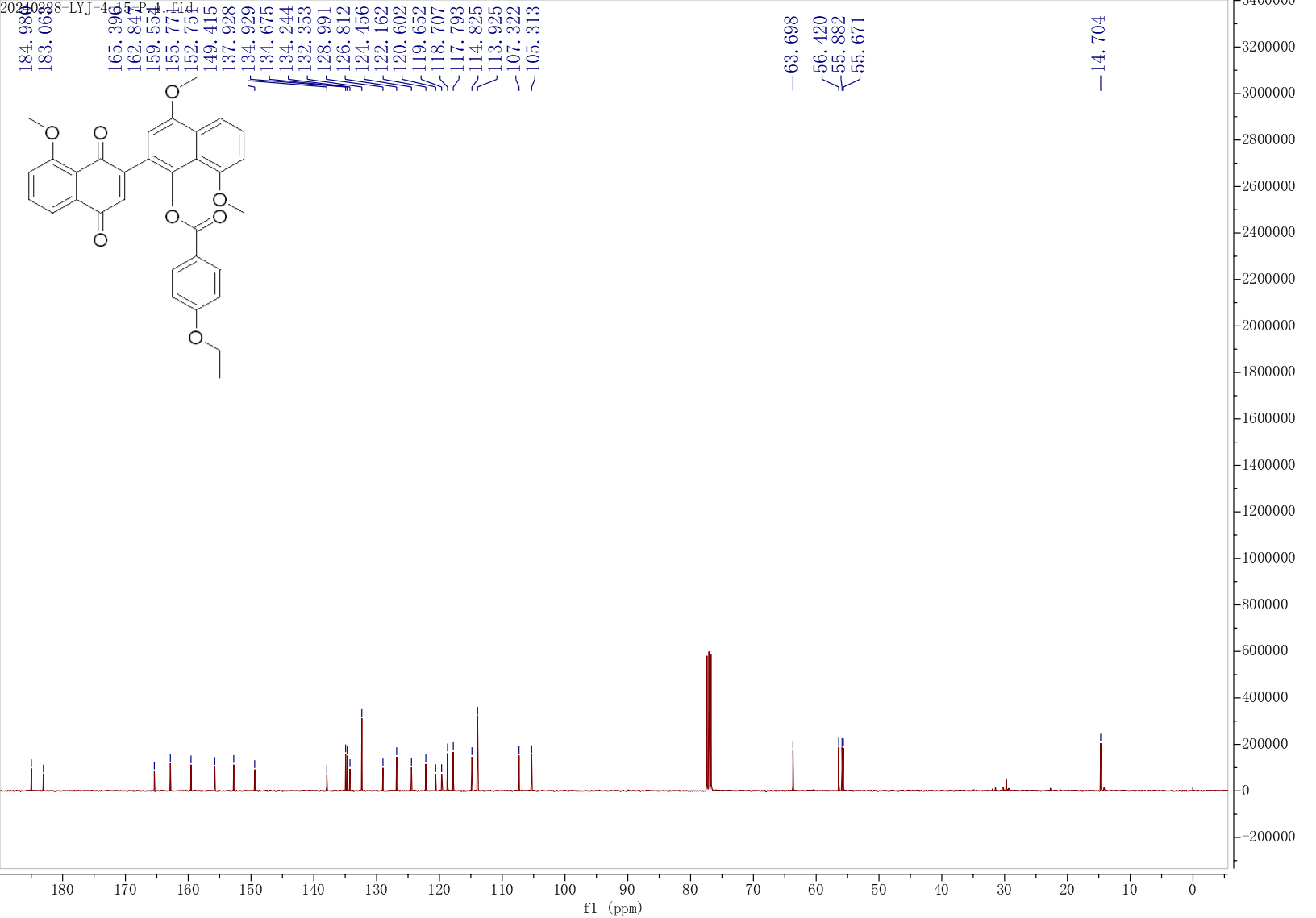


HRMS (ESI) of **10a**


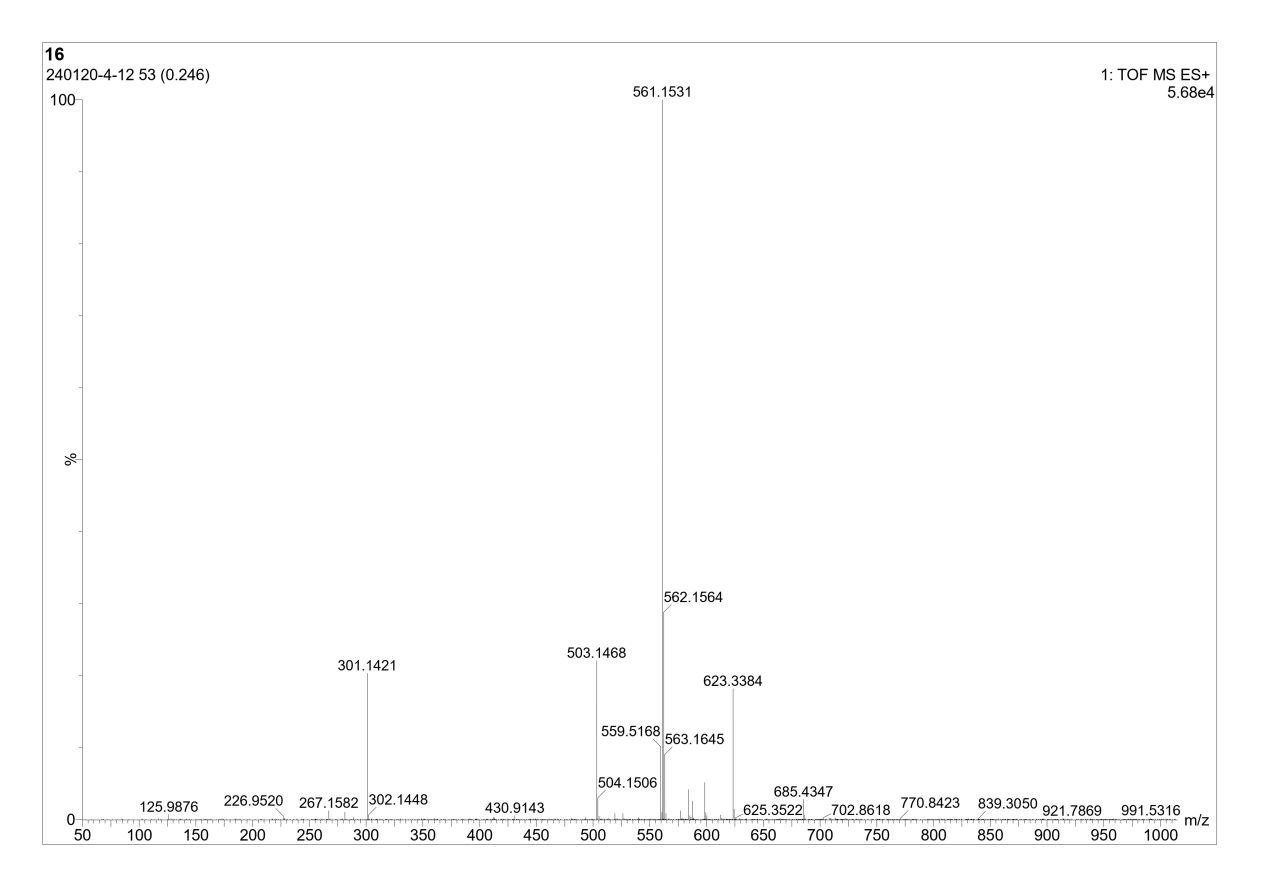


Compound **10b**:

^1^H NMR of **10b** (400 MHz, CDCl_3_)


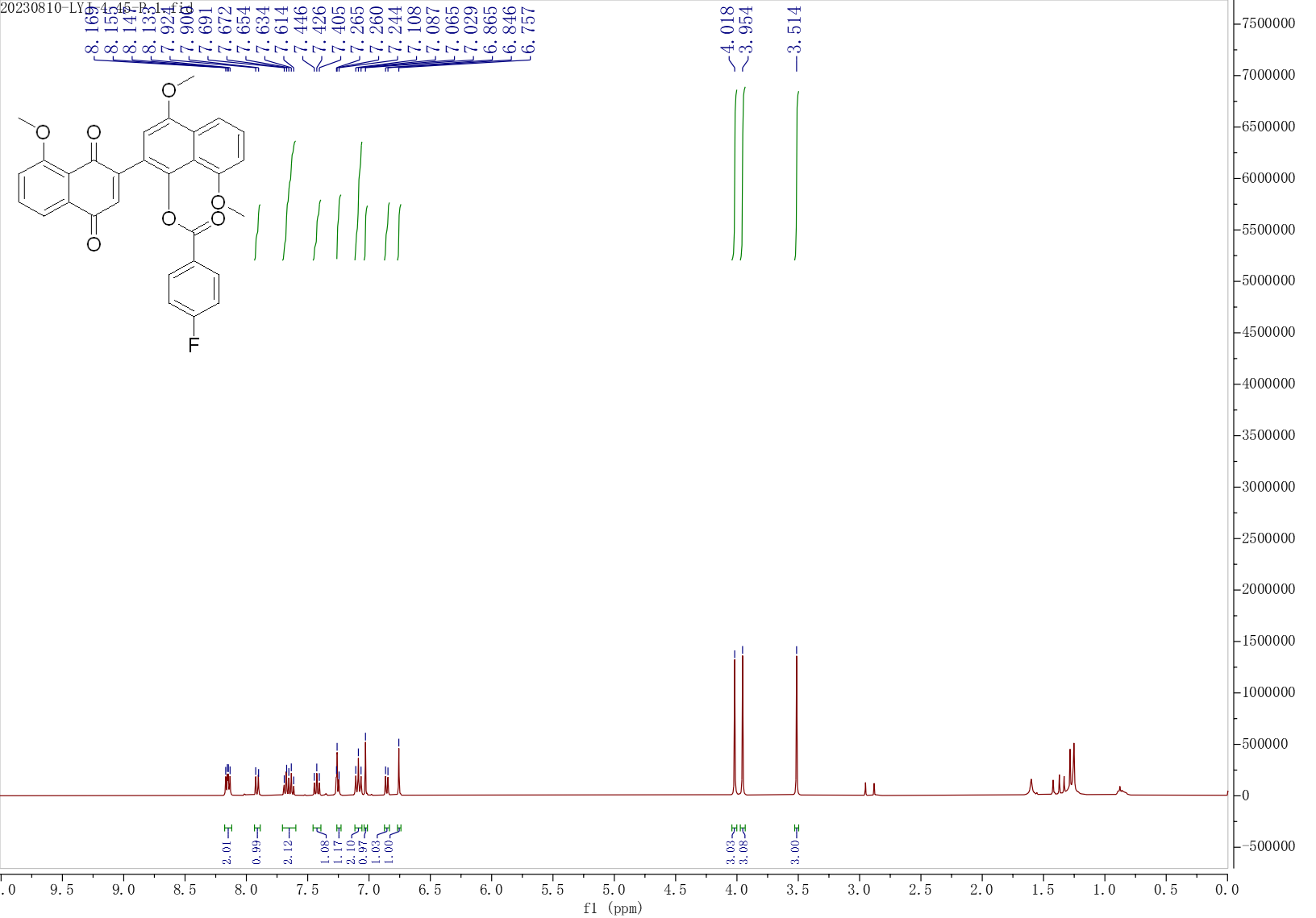


^13^C NMR of **10b** (100 MHz, CDCl_3_)


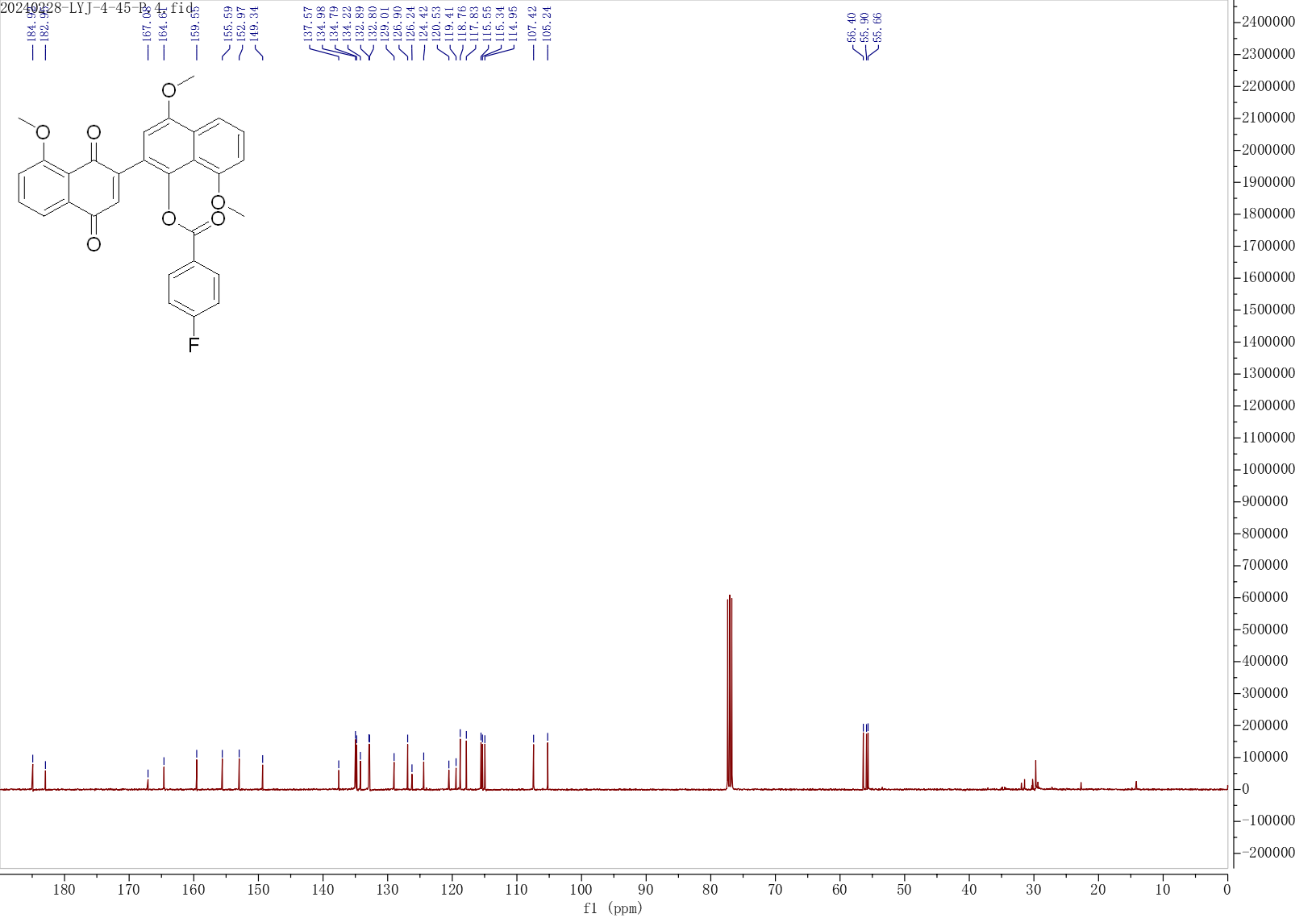


HRMS (ESI) of **10b**


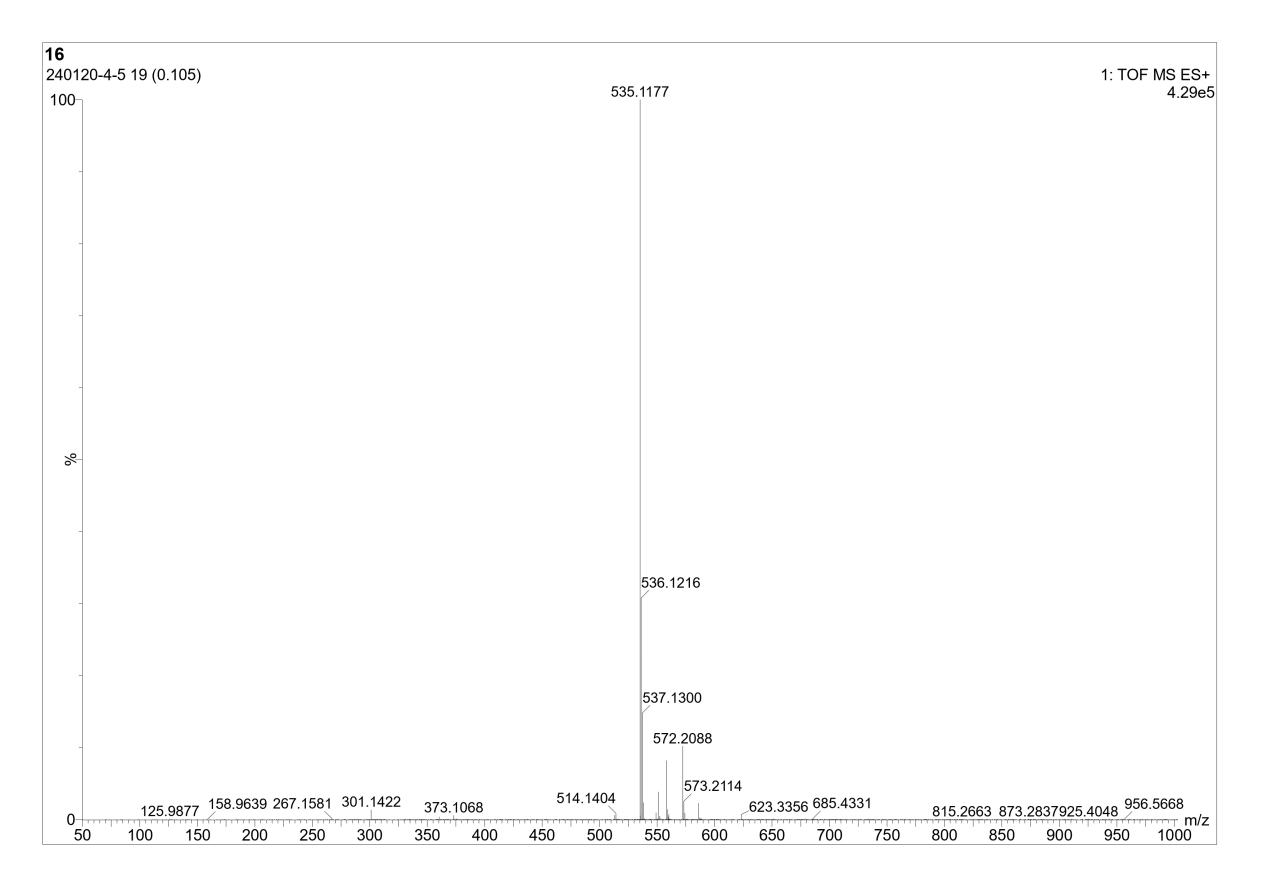


Compound **10c**:

^1^H NMR of **10c** (400 MHz, CDCl_3_)


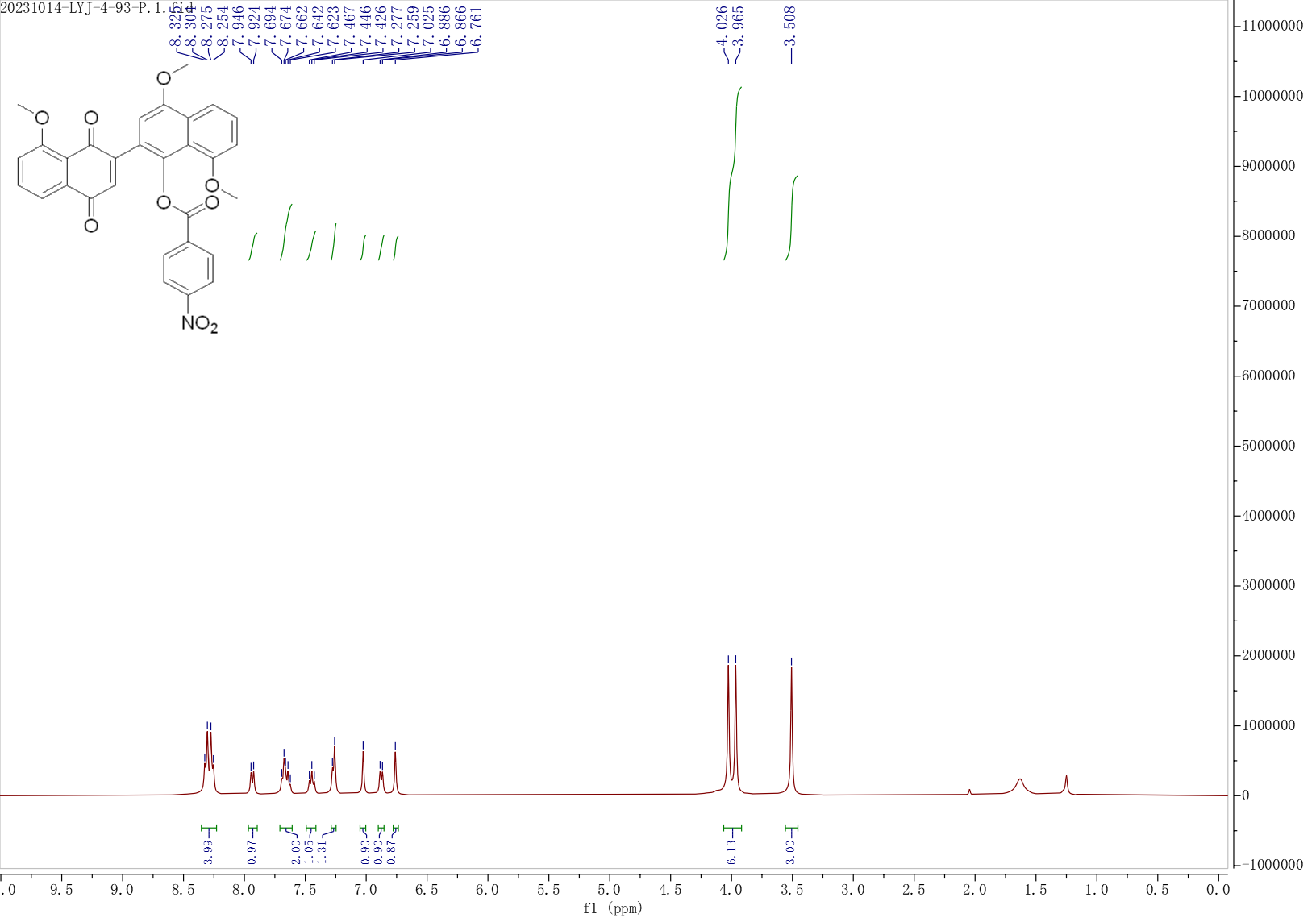


^13^C NMR of **10c** (100 MHz, CDCl_3_)


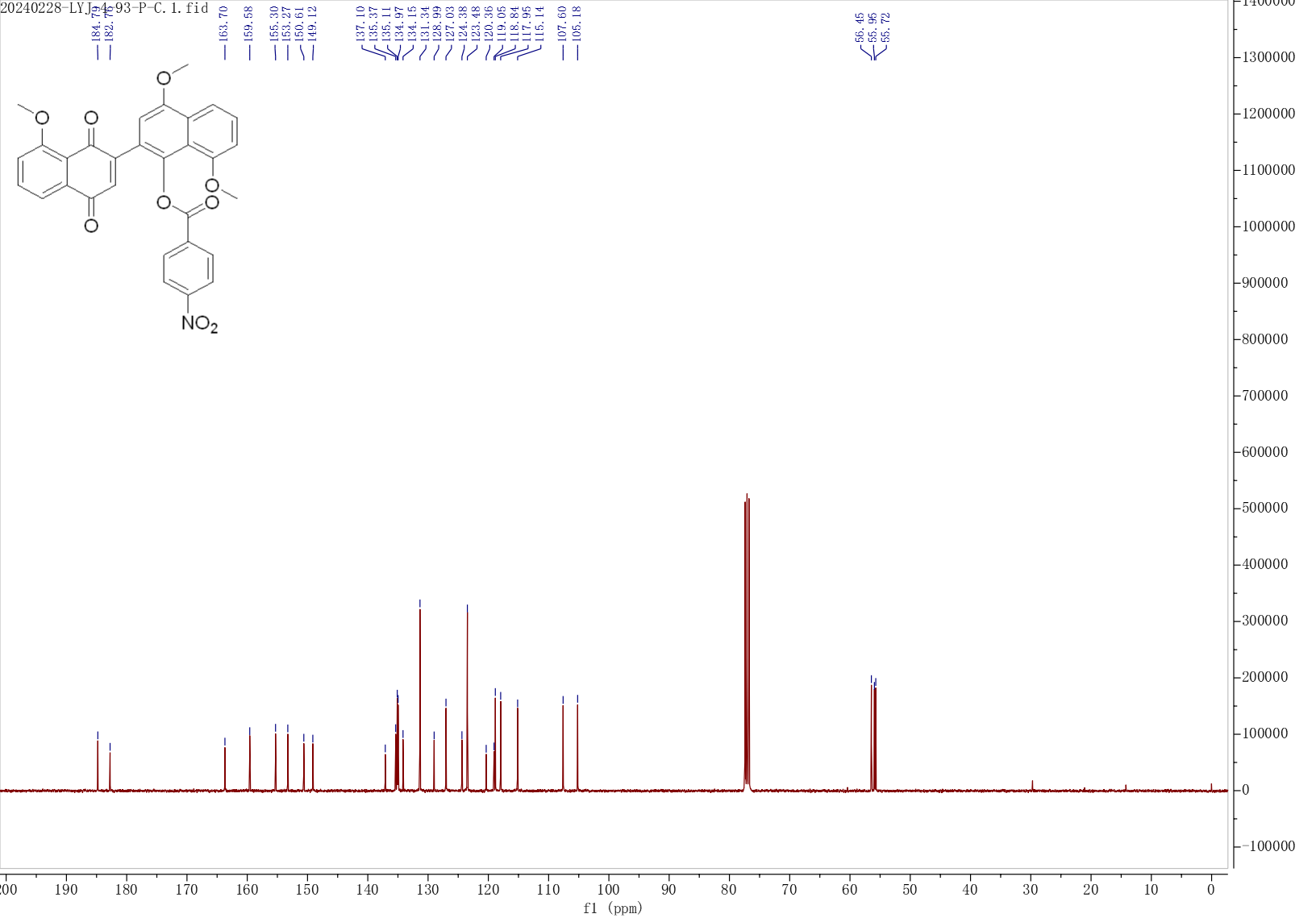


HRMS (ESI) of **10c**


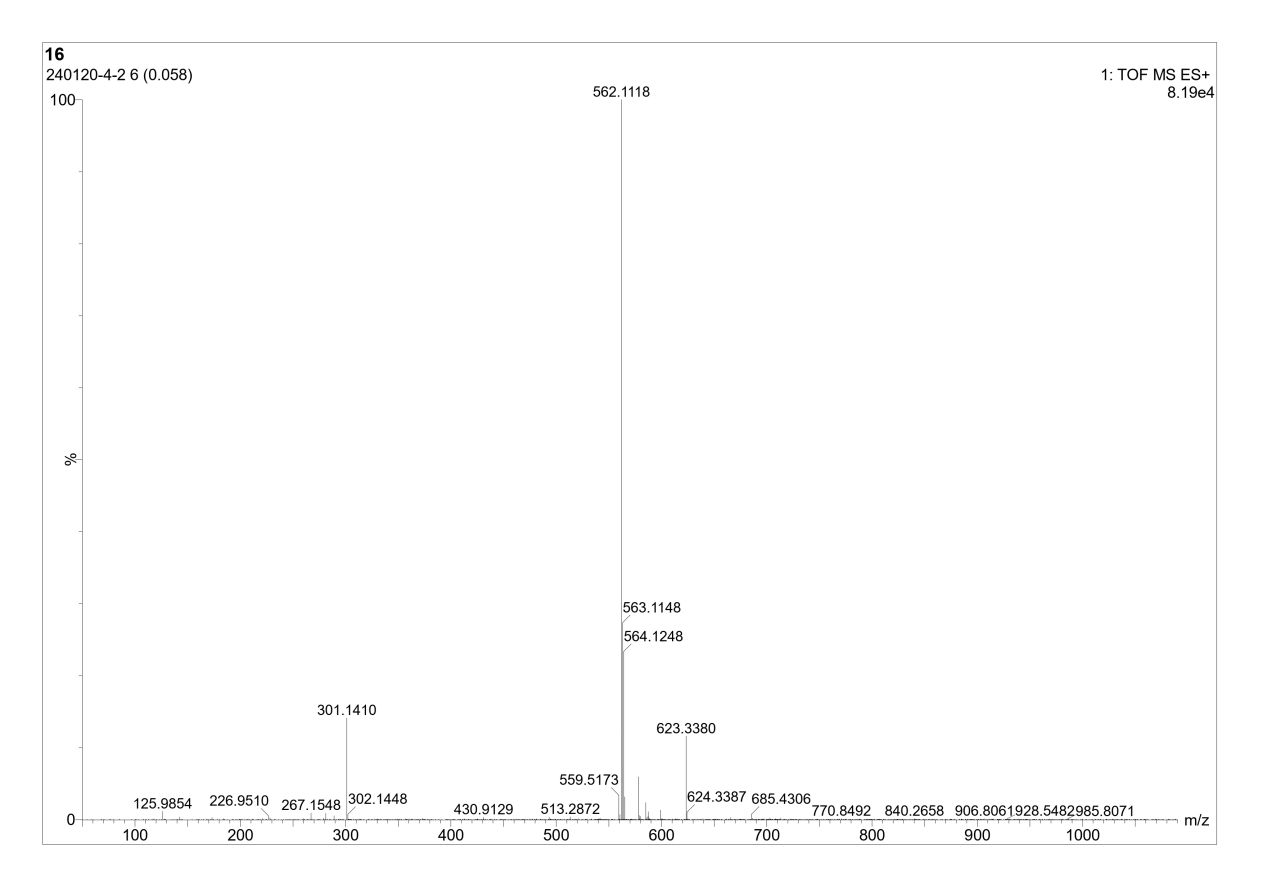


Compound **10d**:

^1^H NMR of **10d** (400 MHz, CDCl_3_)


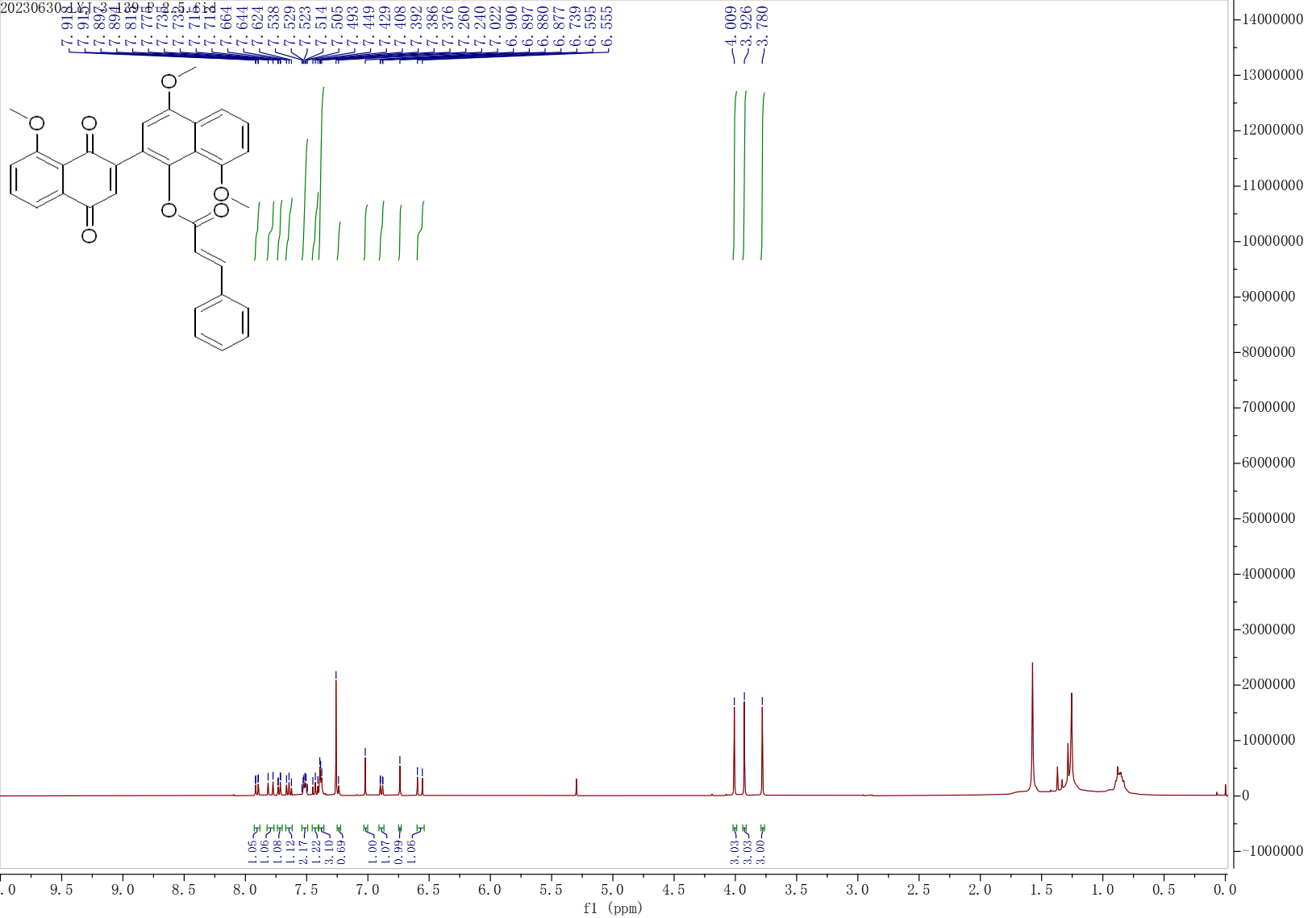


^13^C NMR of **10d** (100 MHz, CDCl_3_)


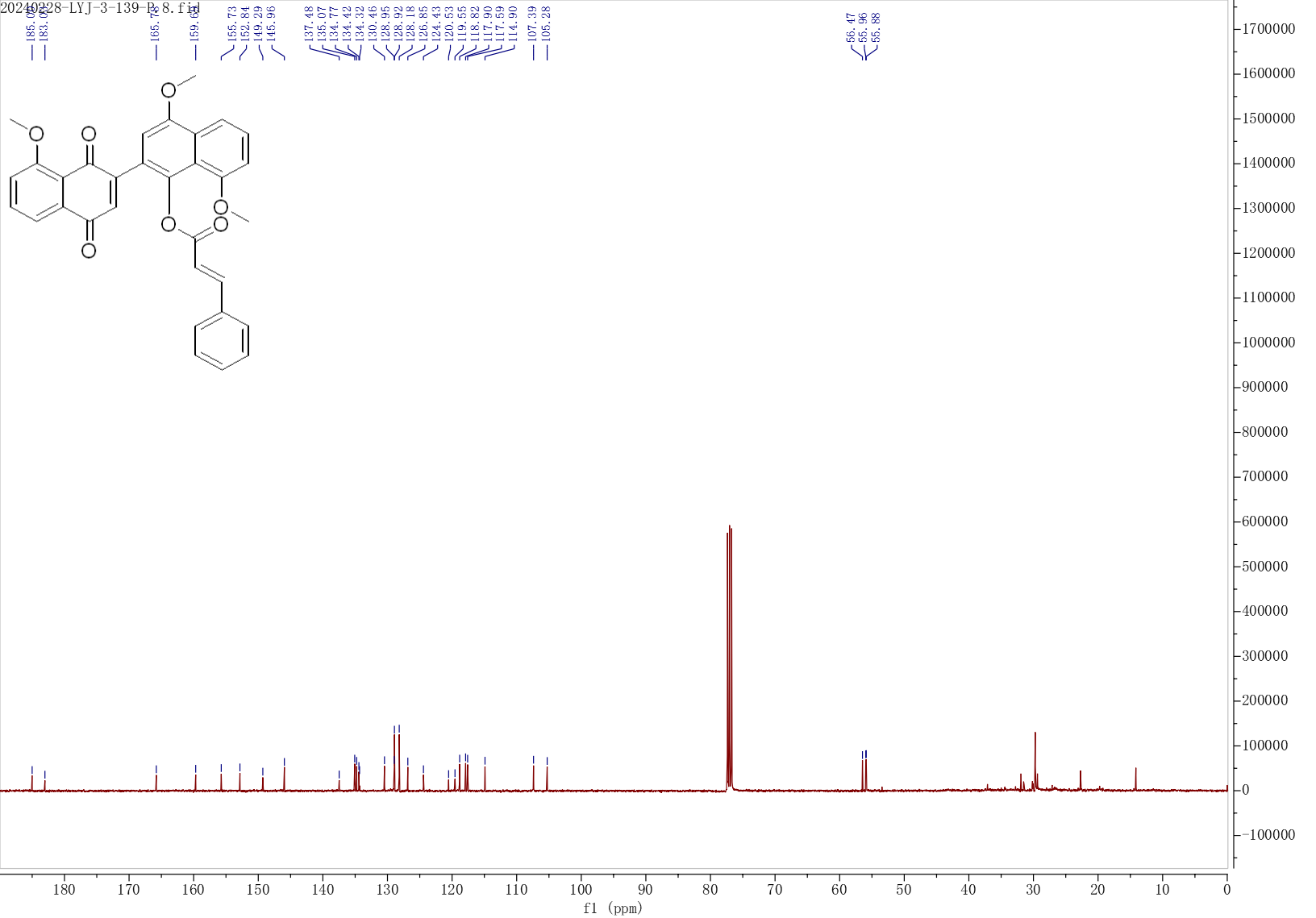


HRMS (ESI) of **10d**


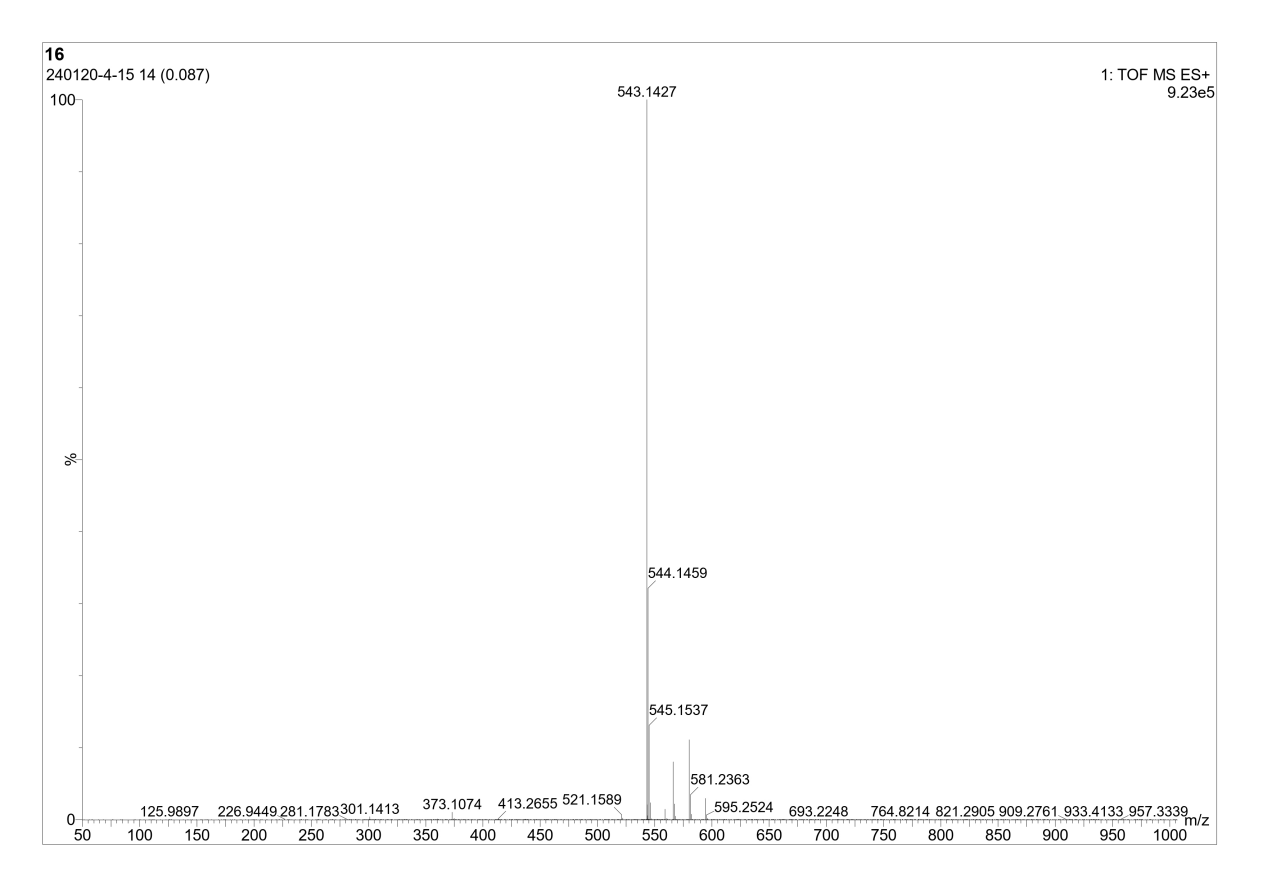


Compound **10e**:

^1^H NMR of **10e** (400 MHz, CDCl_3_)


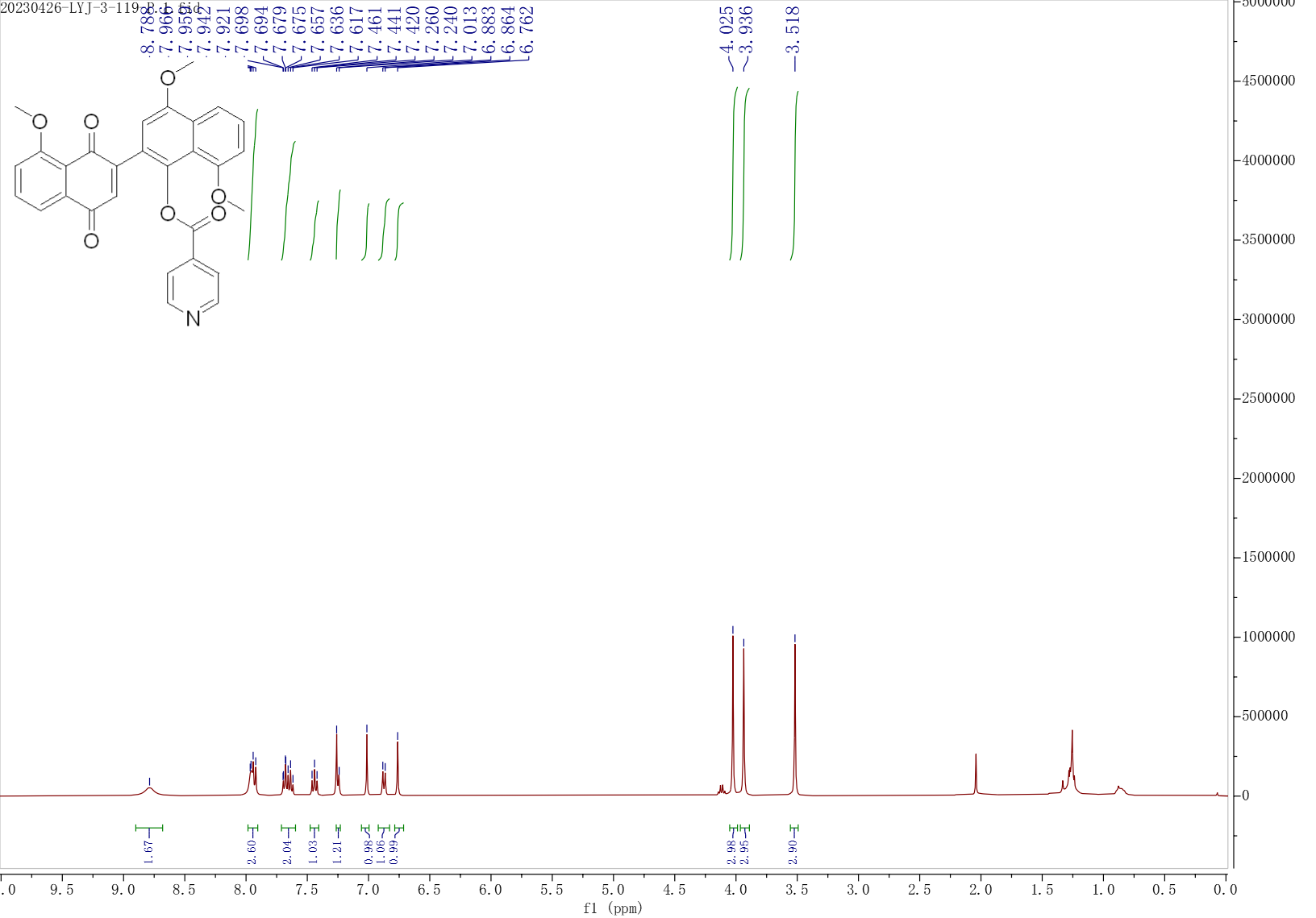


^13^C NMR of **10e** (100 MHz, CDCl_3_)


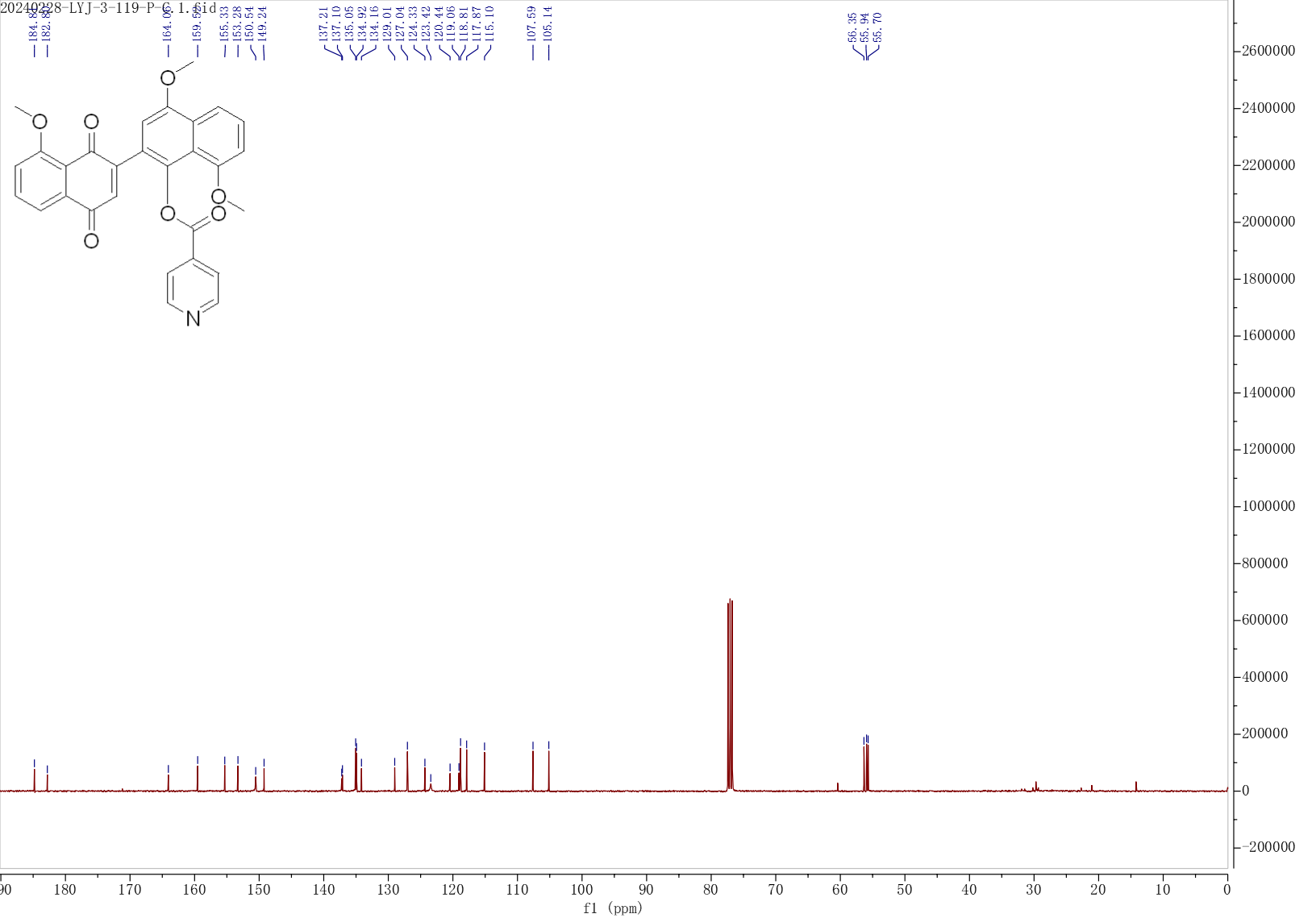


HRMS (ESI) of **10e**


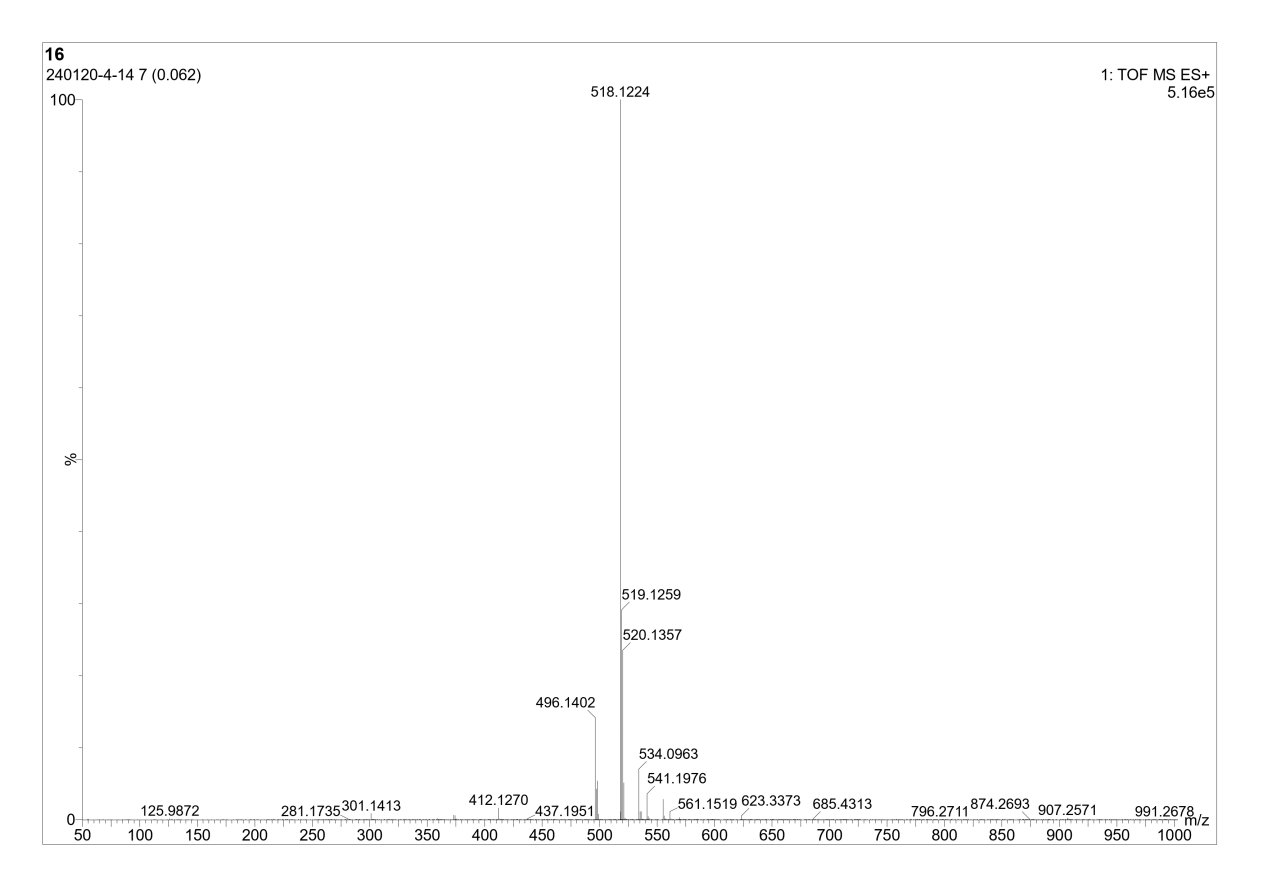


Compound **10f**:

^1^H NMR of **10f** (400 MHz, CDCl_3_)


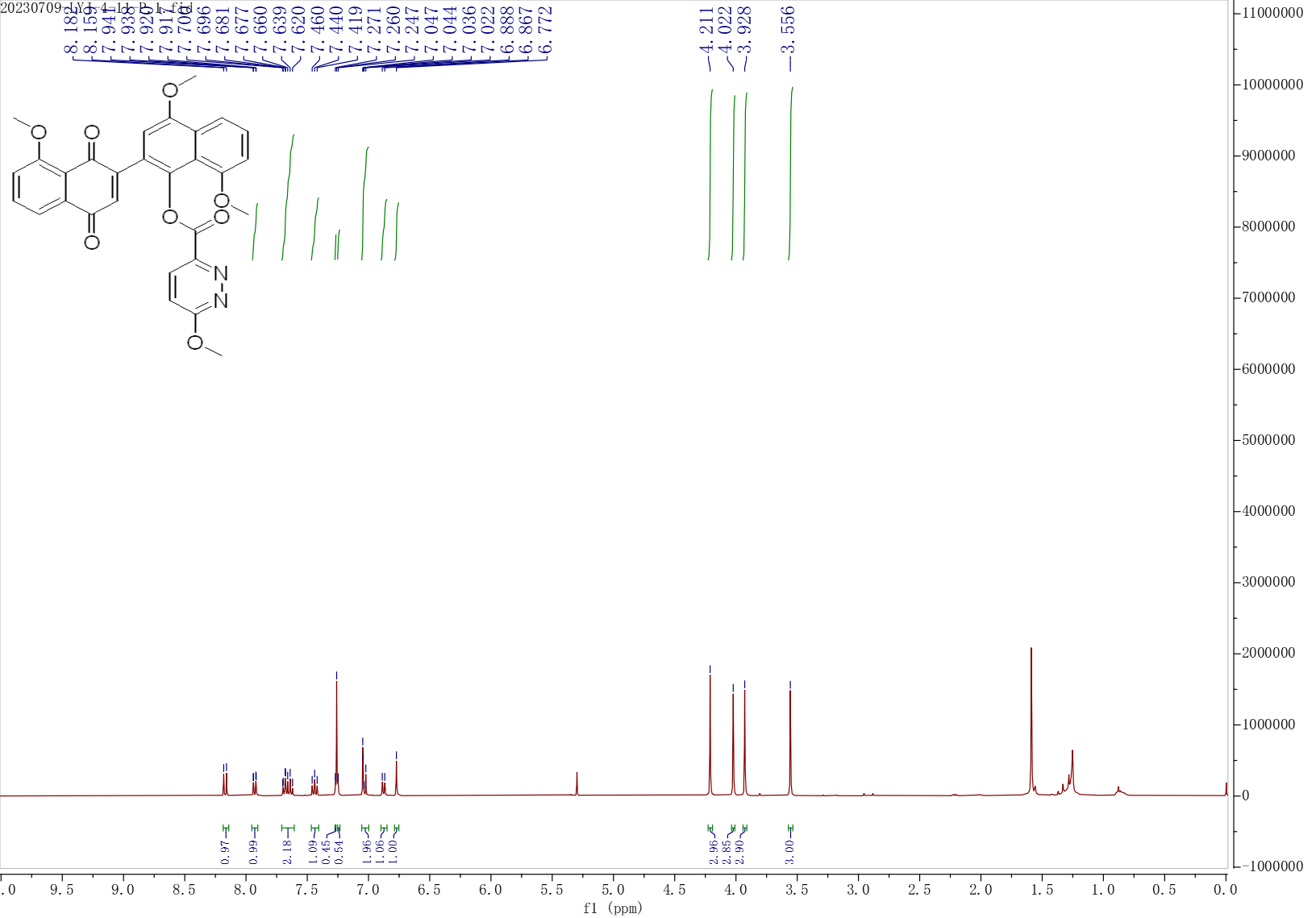


^13^C NMR of **10f** (100 MHz, CDCl_3_)


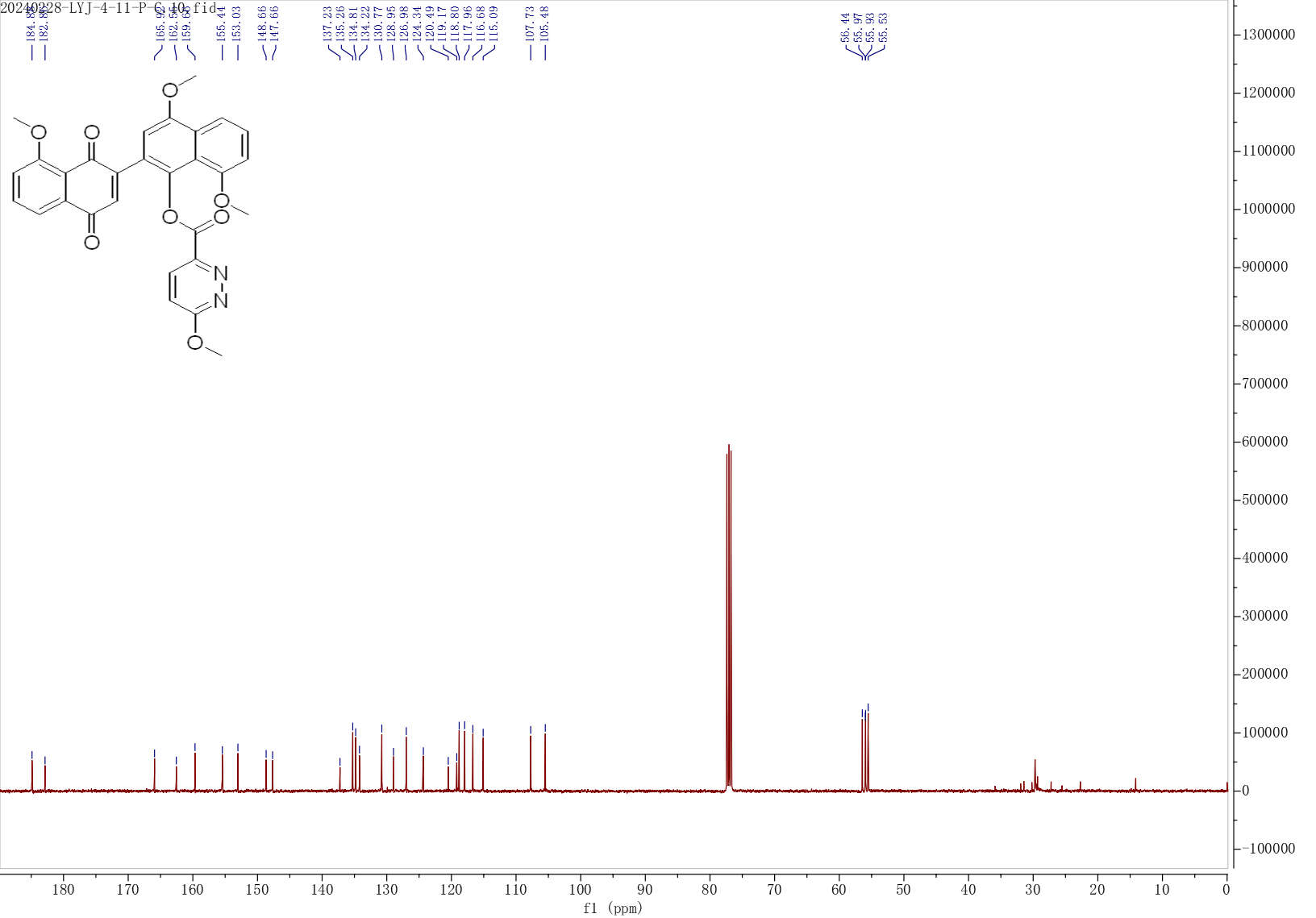


HRMS (ESI) of **10f**


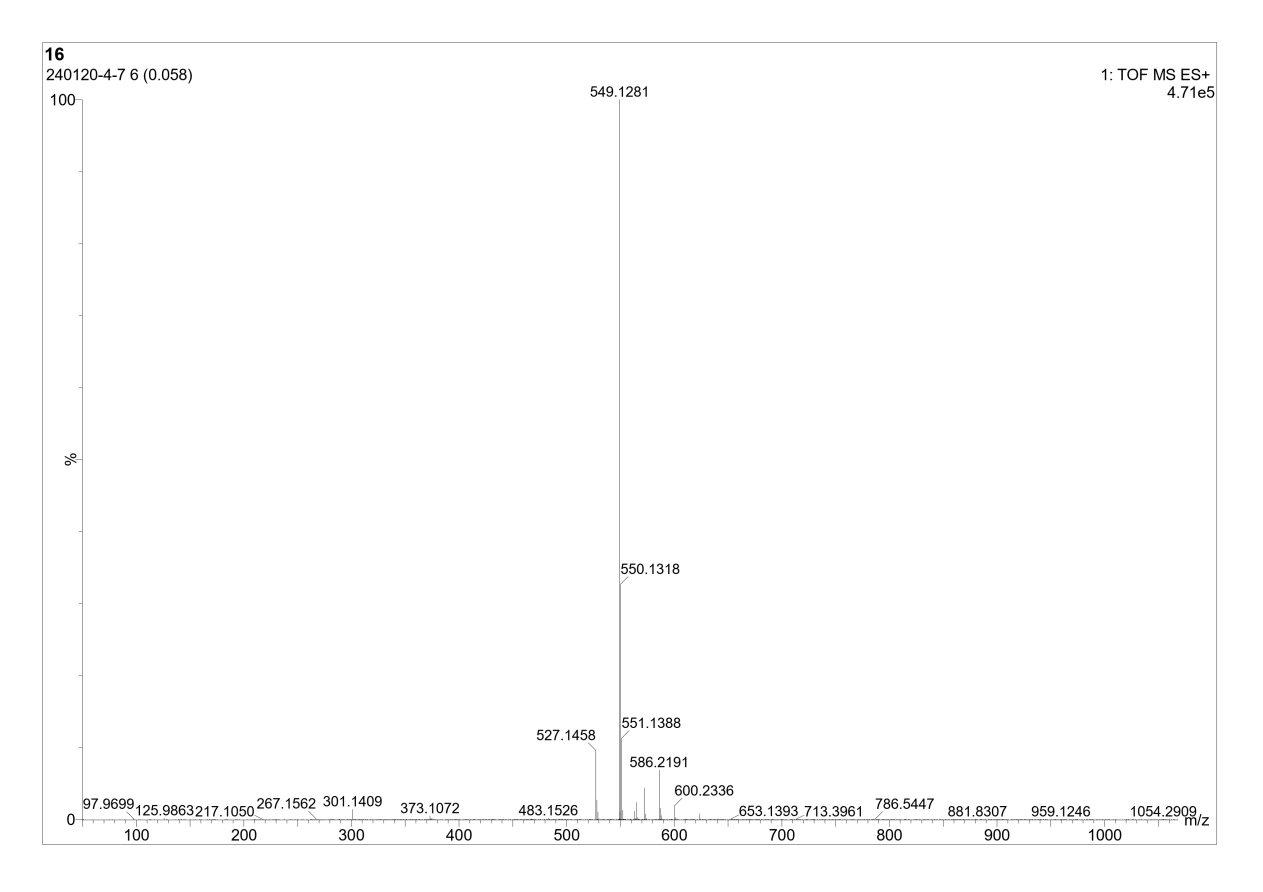


Compound **10g**:

^1^H NMR of **10g** (400 MHz, CDCl_3_)


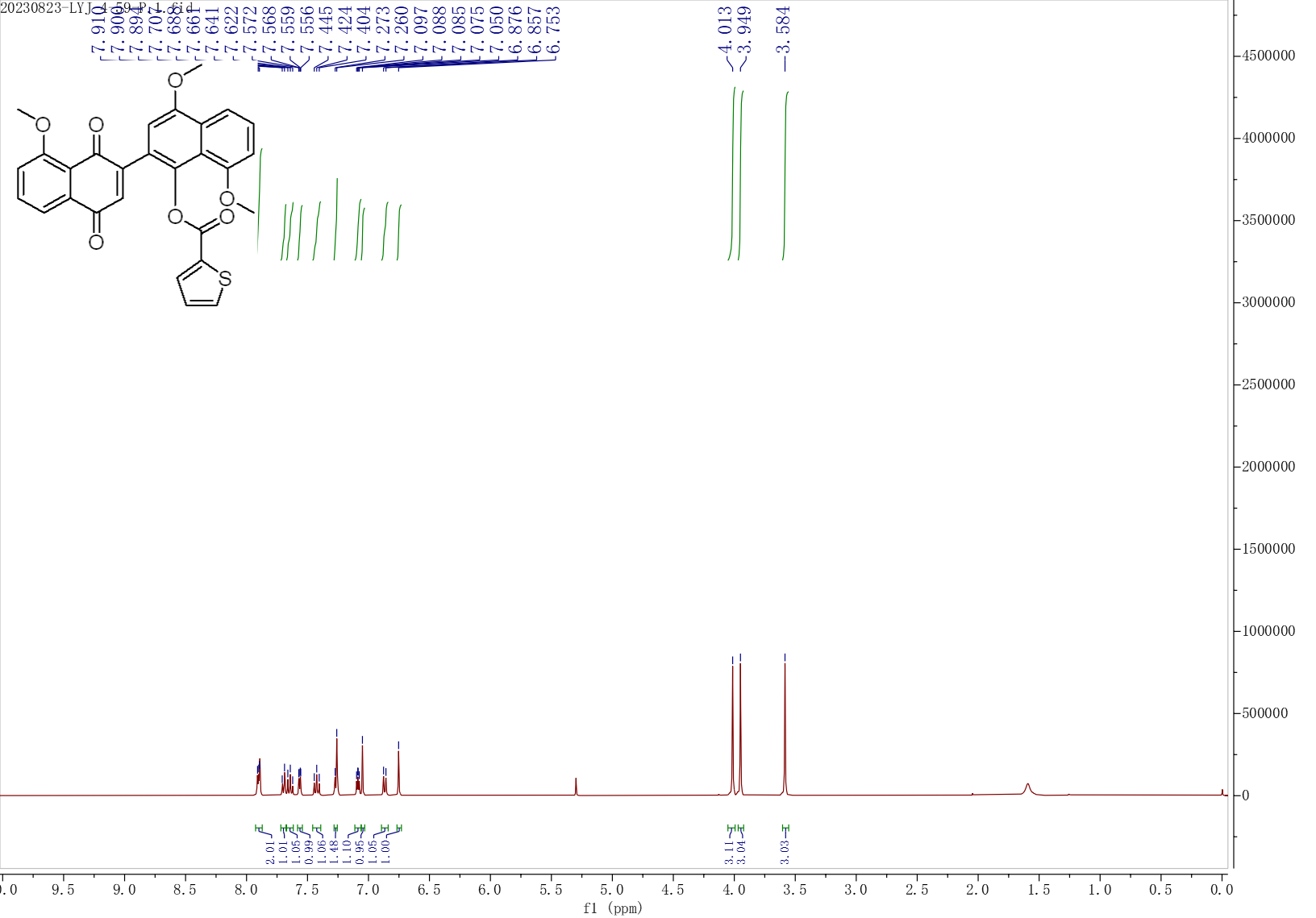


^13^C NMR of **10g** (100 MHz, CDCl_3_)


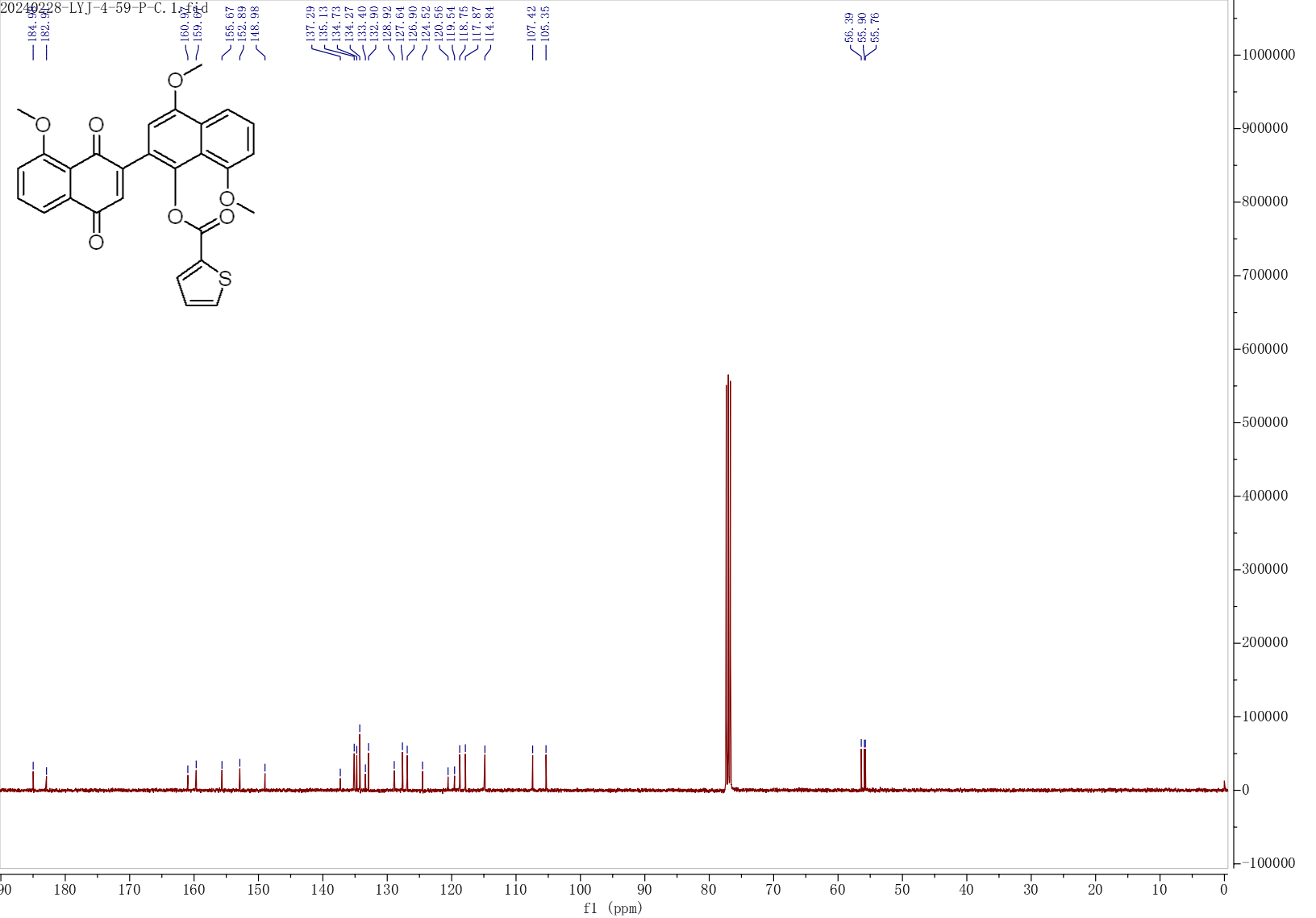


HRMS (ESI) of **10g**


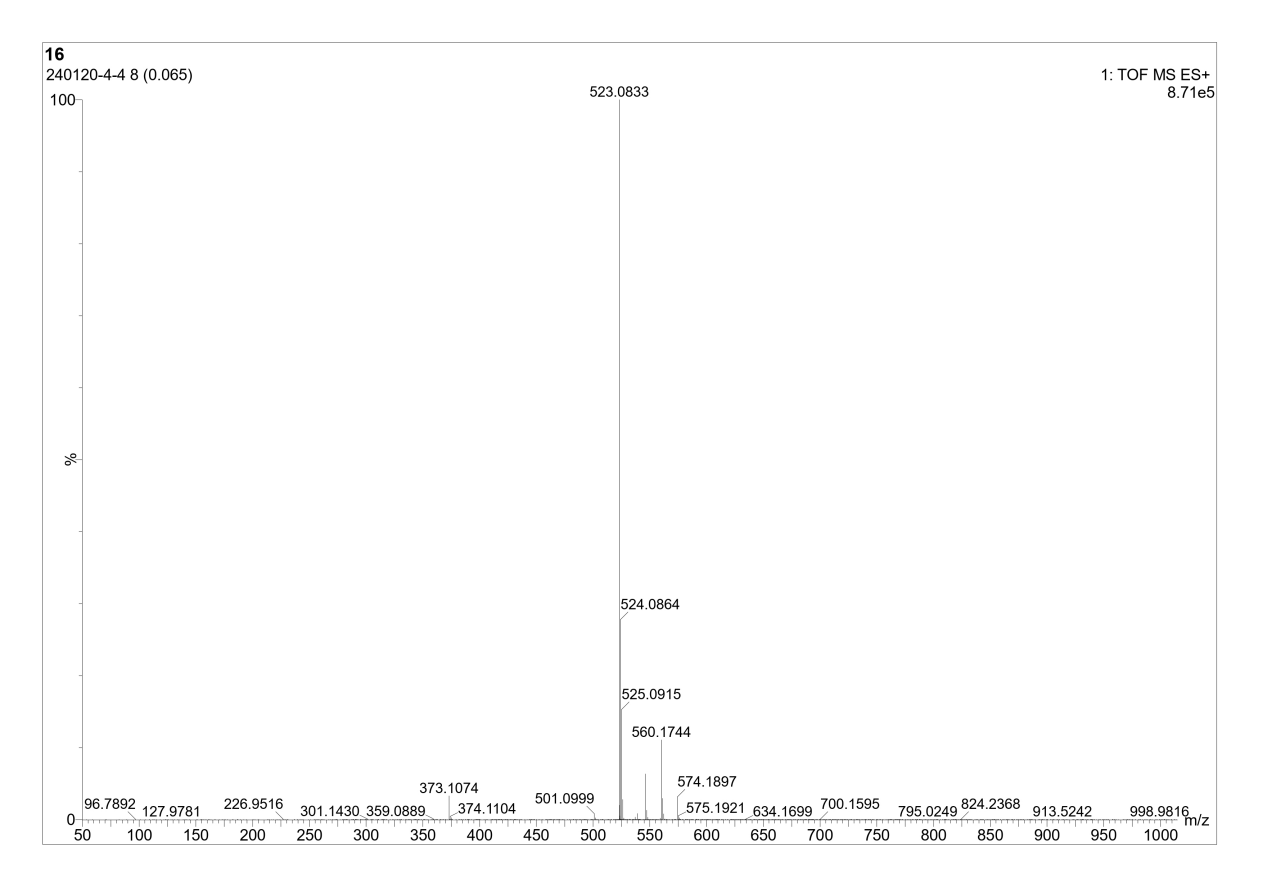


Compound **10h**:

^1^H NMR of **10h** (400 MHz, CDCl_3_)


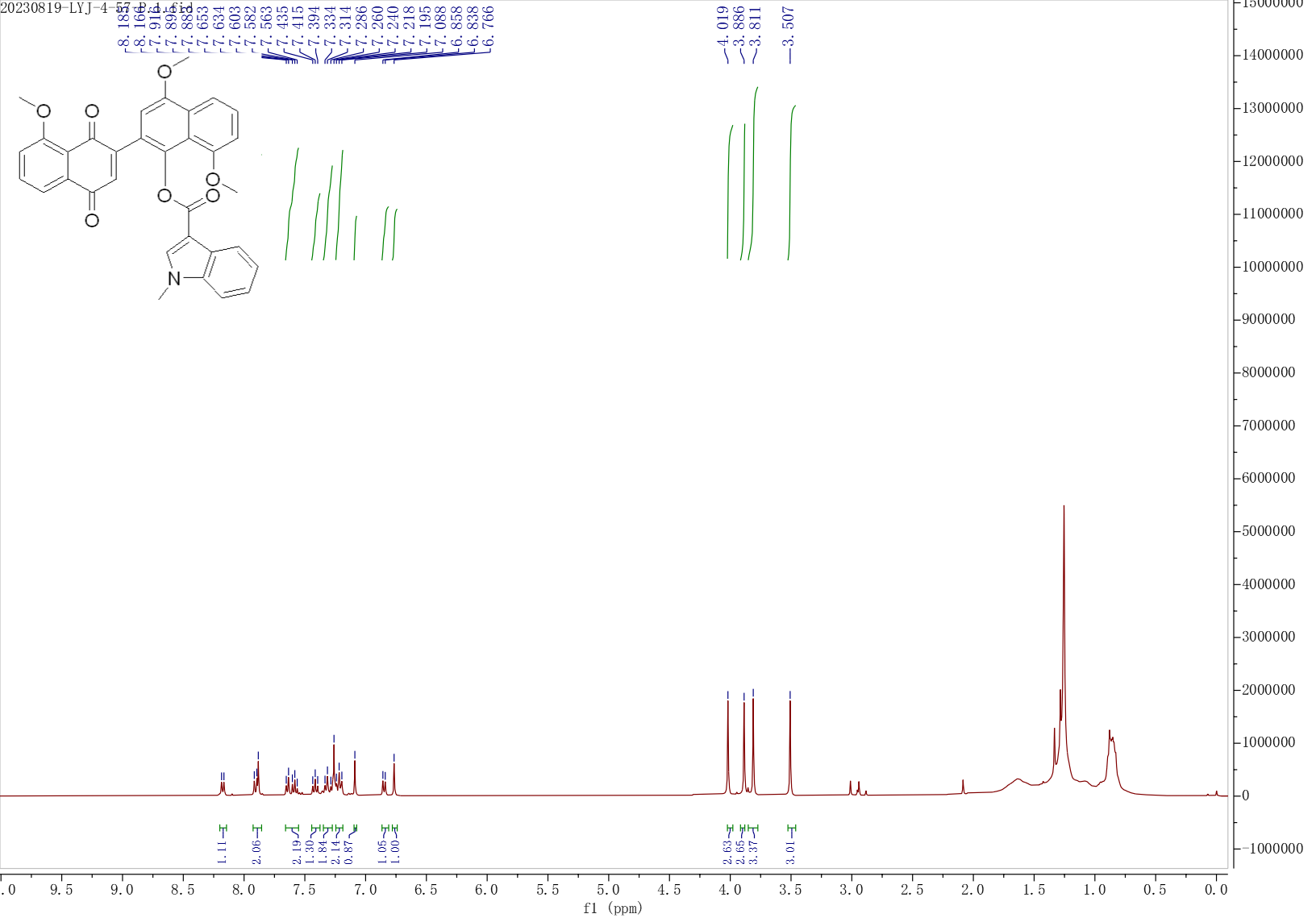


^13^C NMR of **10h** (100 MHz, CDCl_3_)


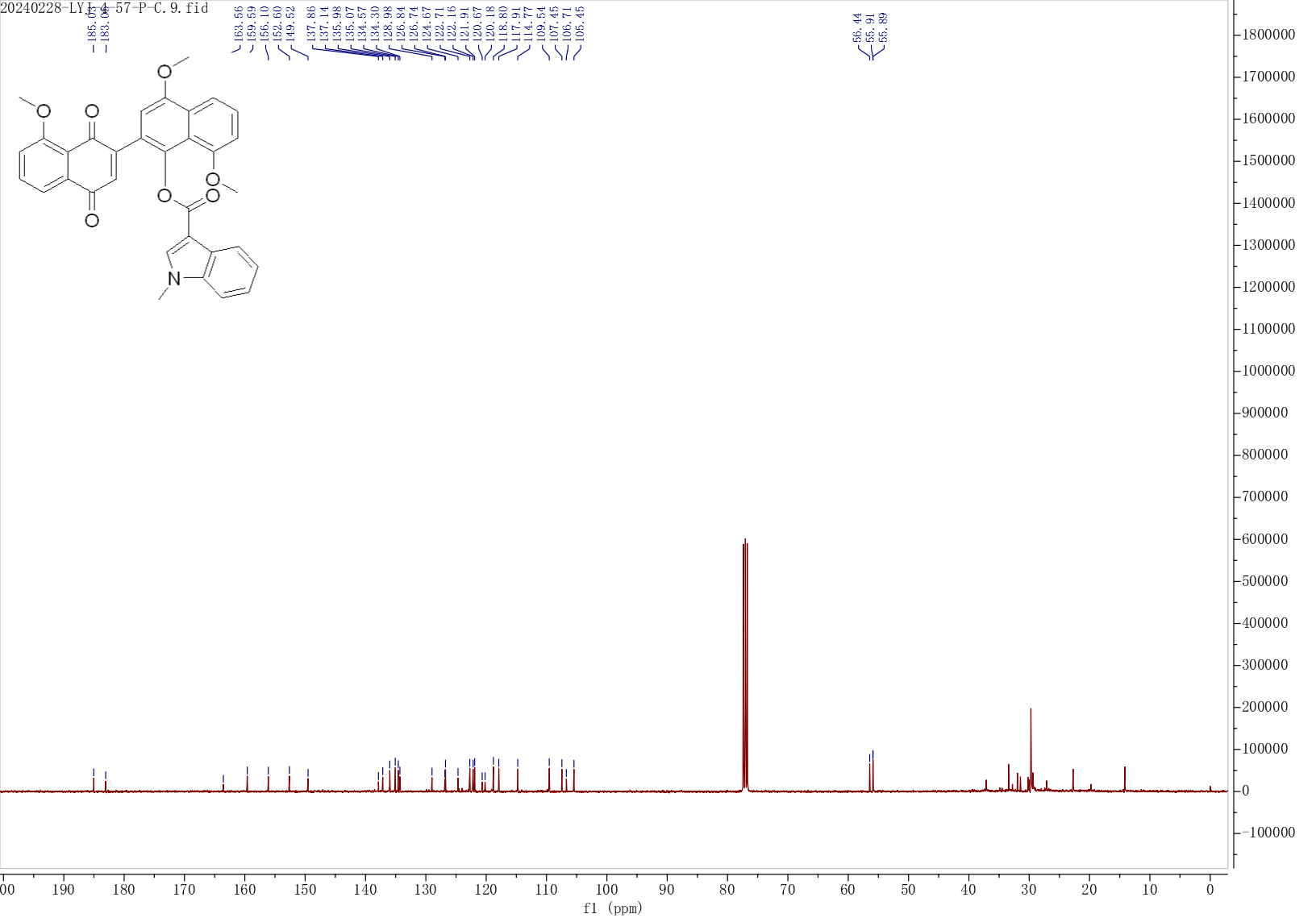


HRMS (ESI) of **10h**


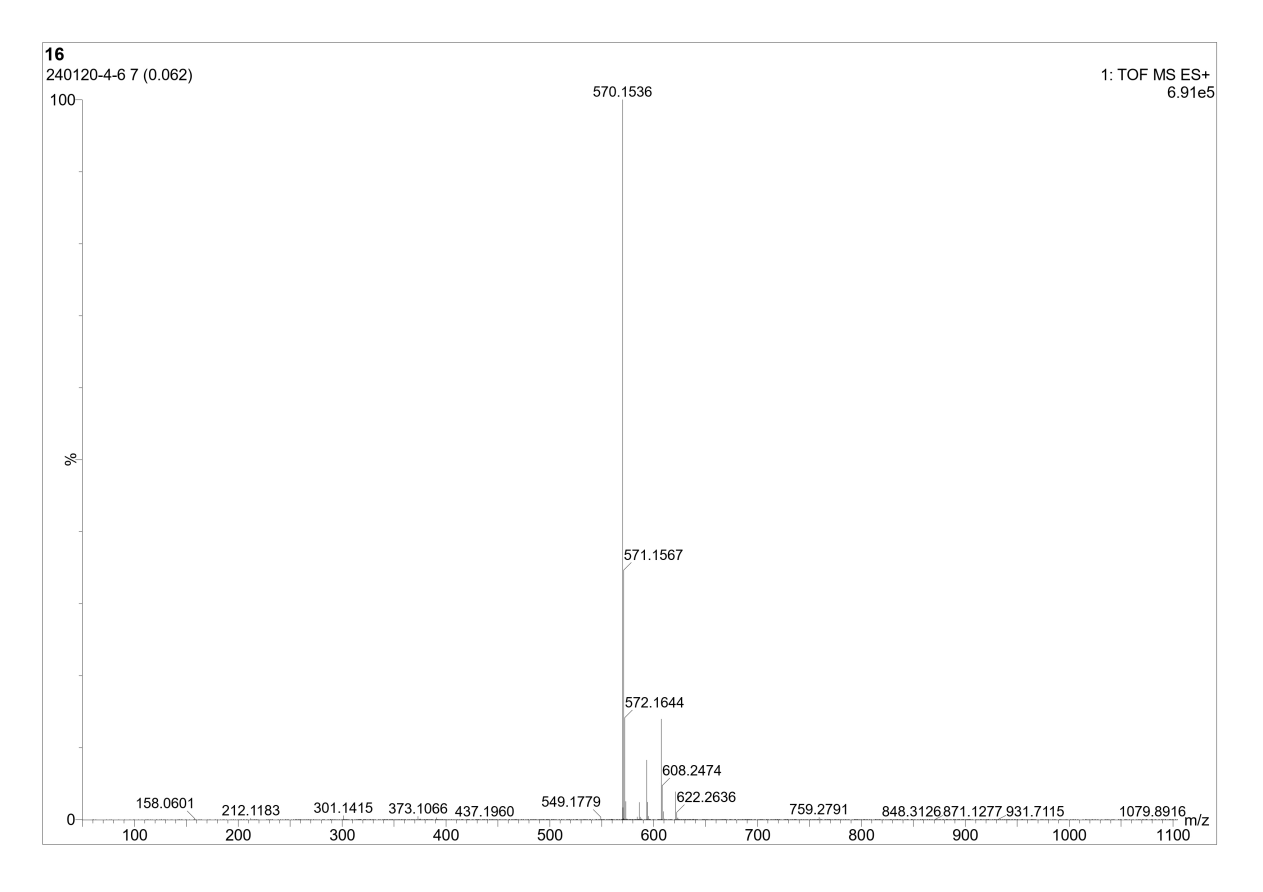


Compound **10i**:

^1^H NMR of **10i** (400 MHz, CDCl_3_)


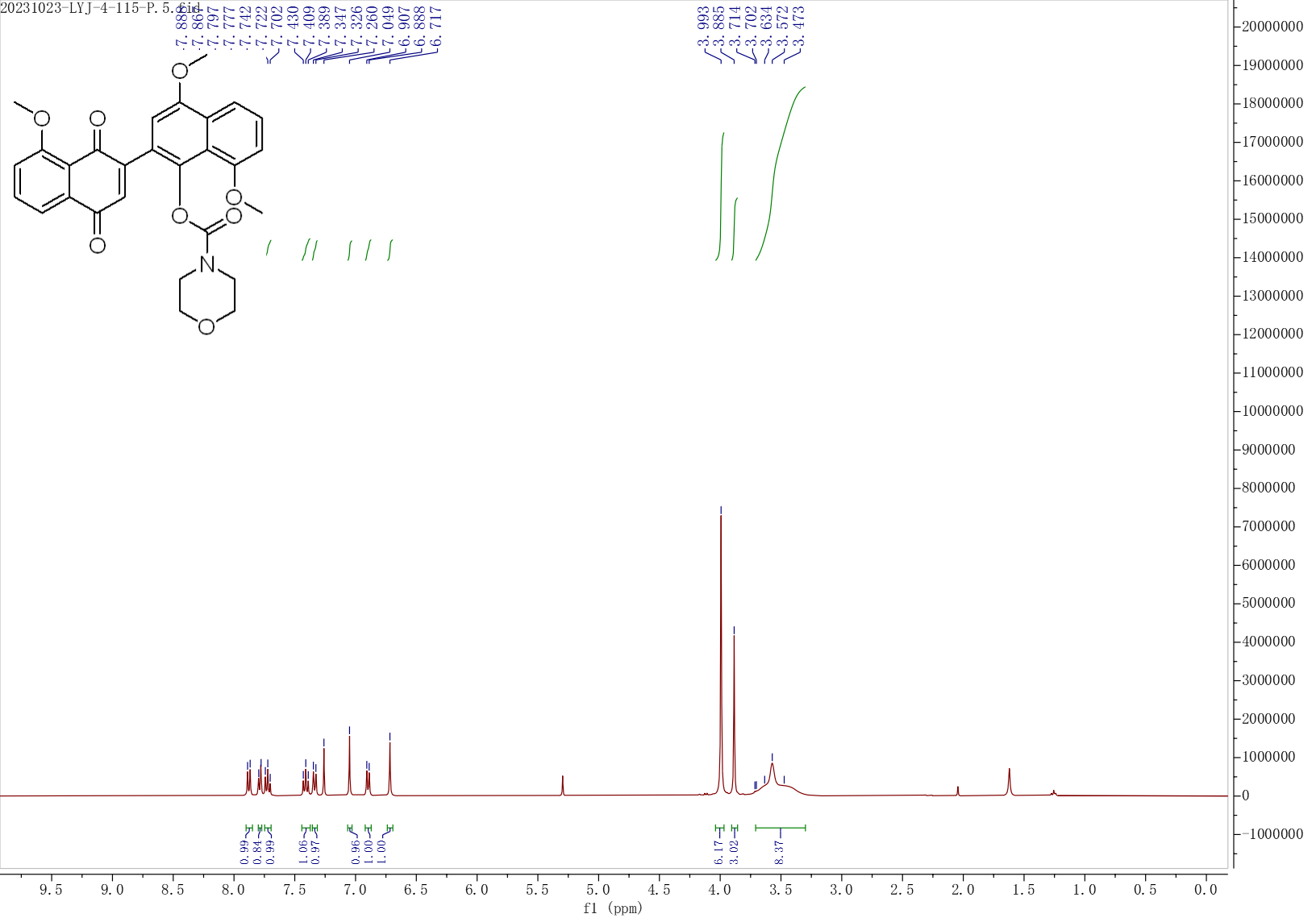


^13^C NMR of **10i** (100 MHz, CDCl_3_)


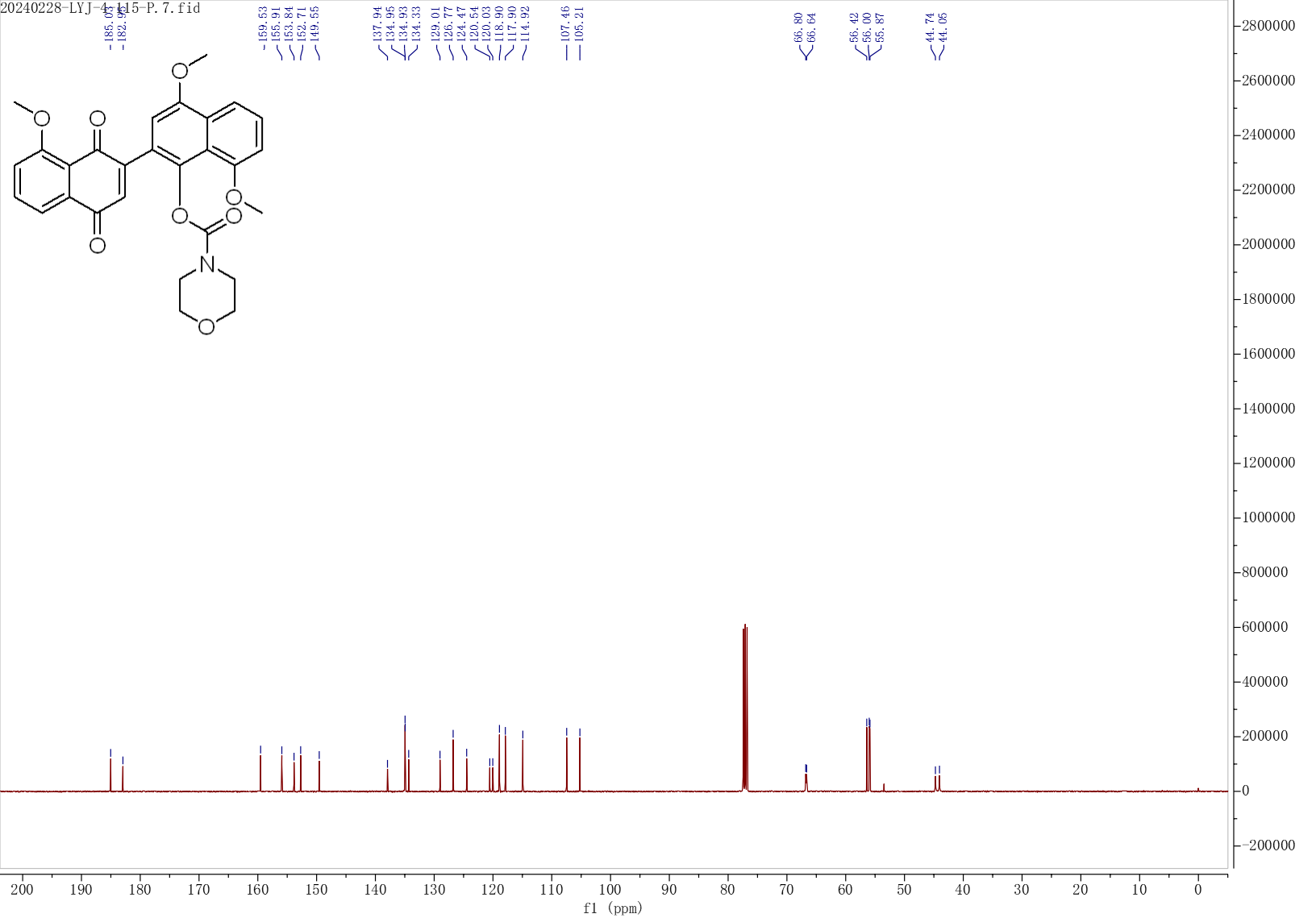


HRMS (ESI) of **10i**


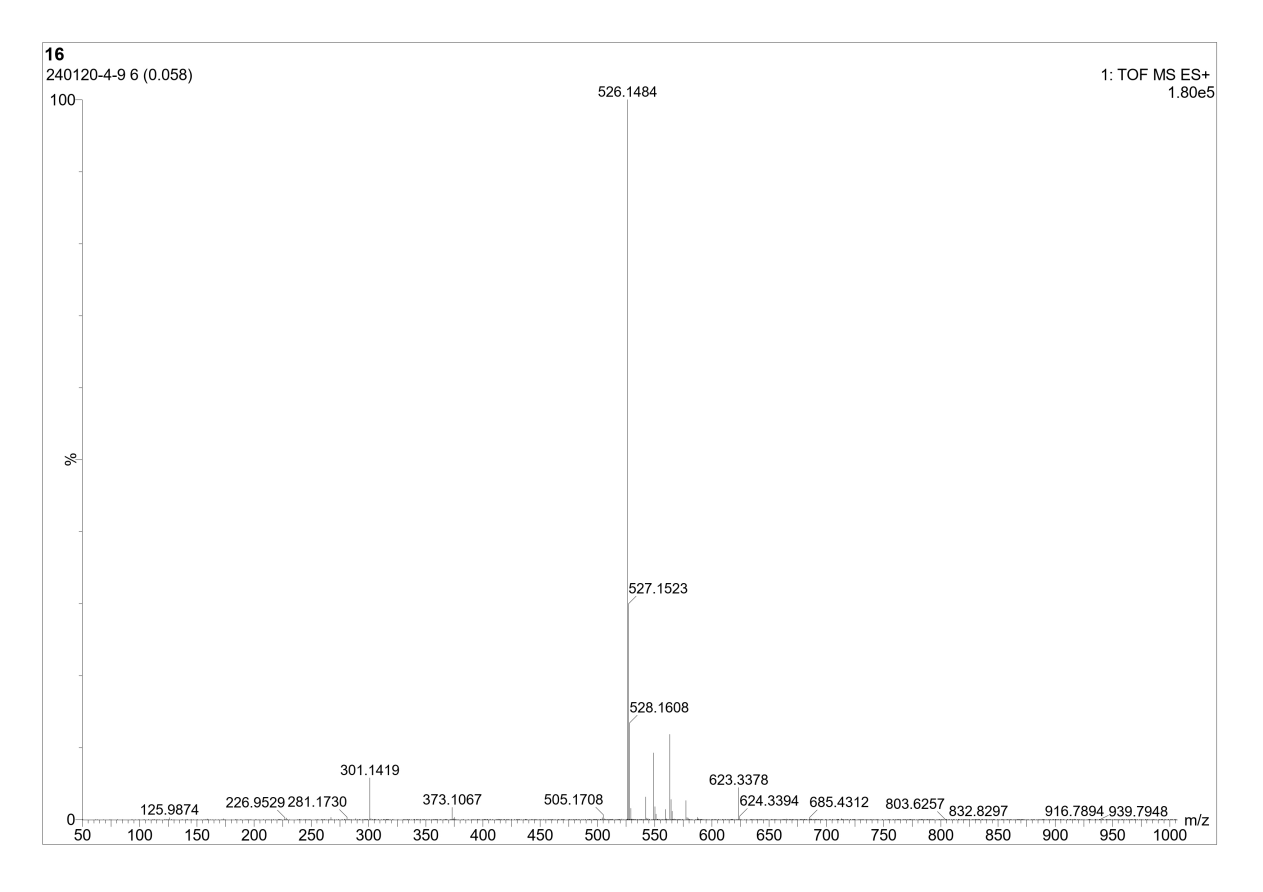


Compound **10j**:

^1^H NMR of **10j** (400 MHz, CDCl_3_)


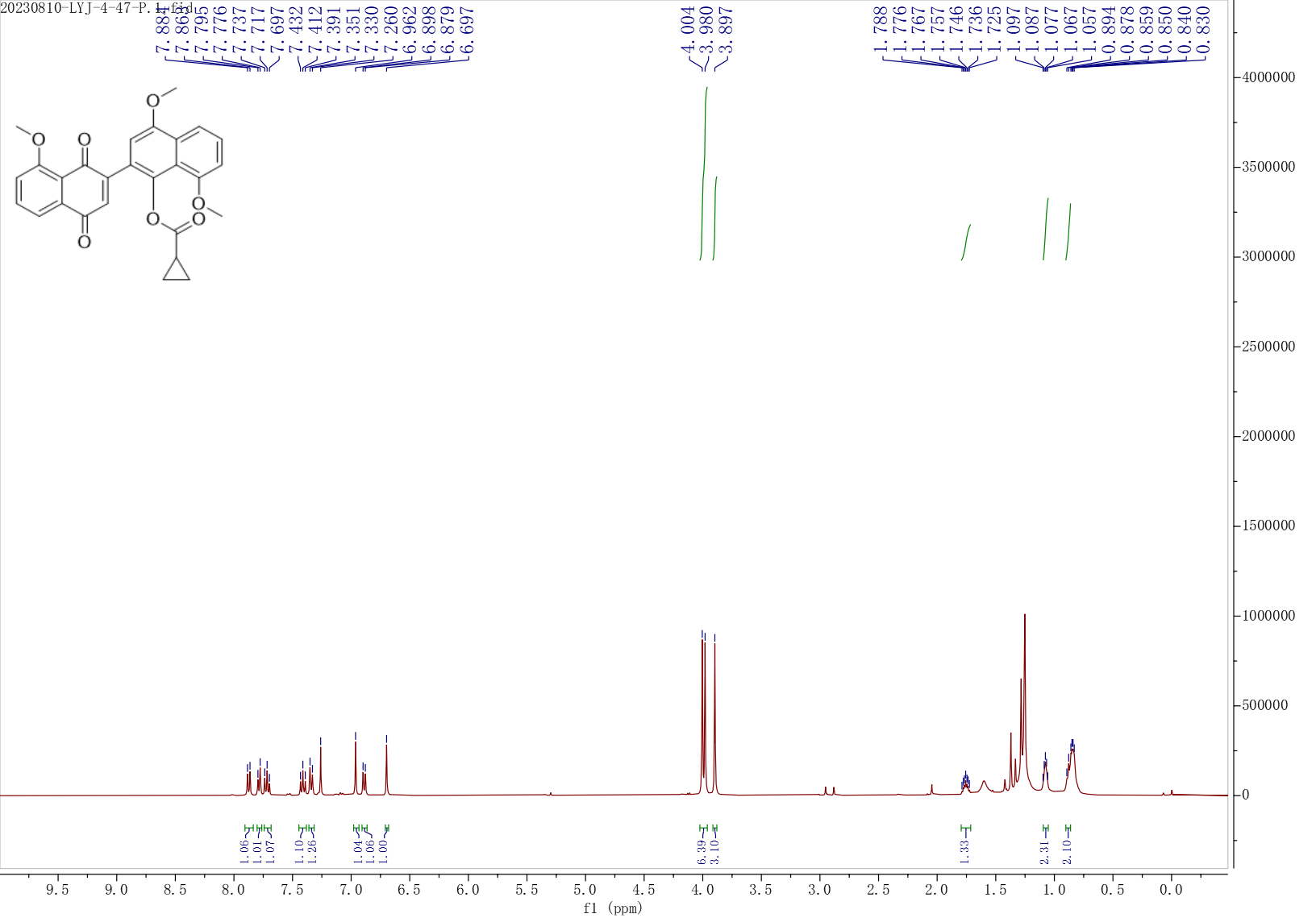


^13^C NMR of **10j** (100 MHz, CDCl_3_)


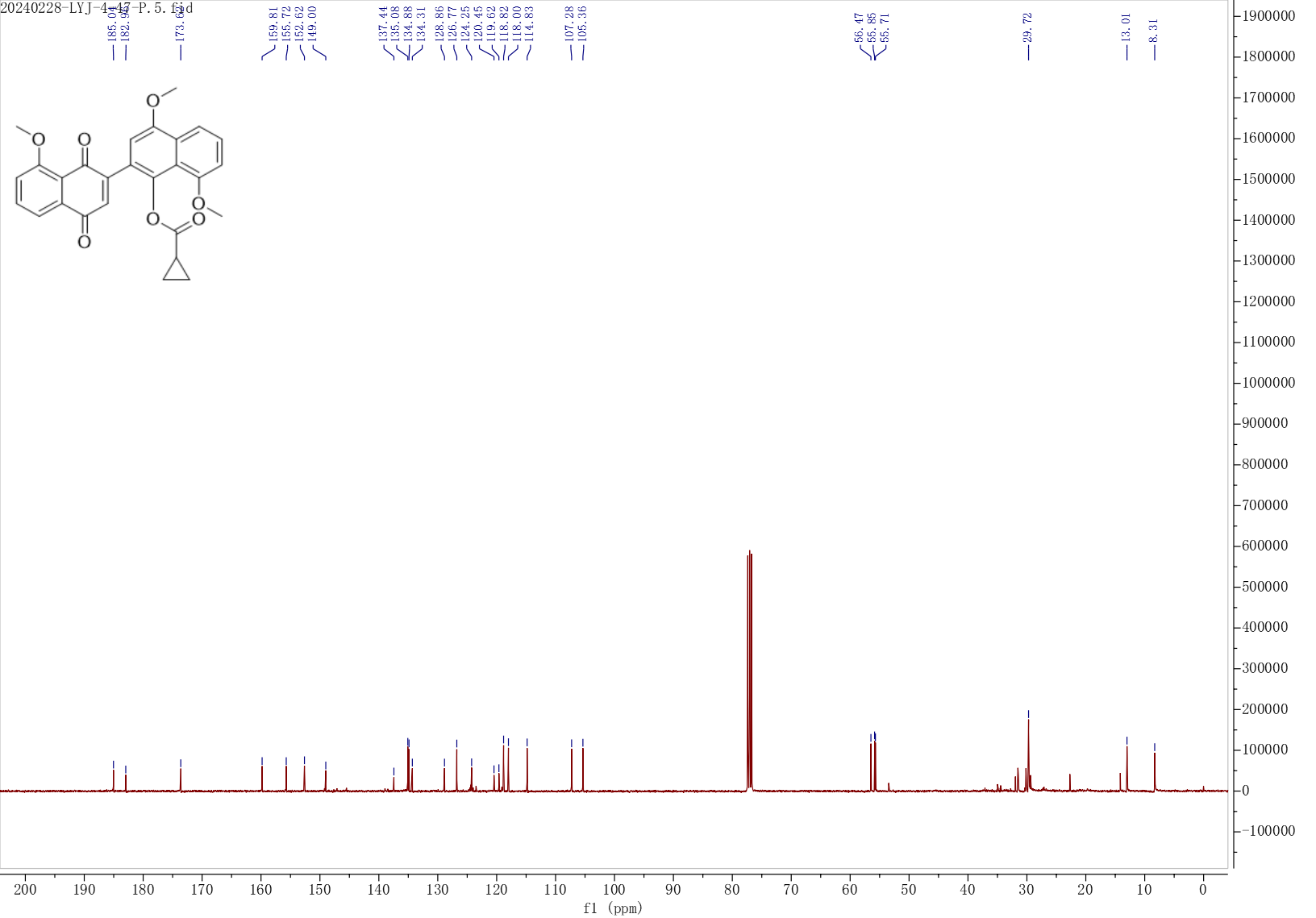


HRMS (ESI) of **10j**


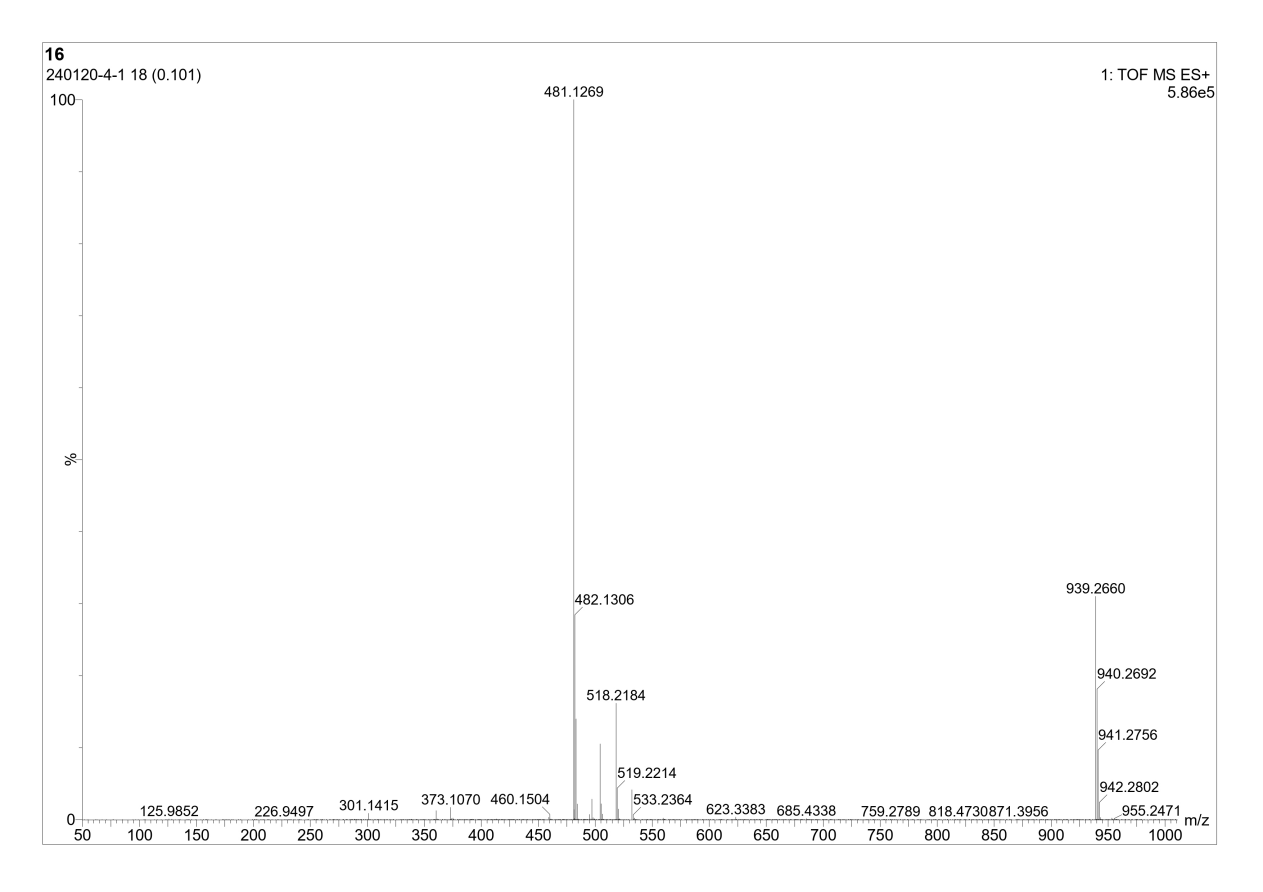


Compound **10k**:

^1^H NMR of **10k** (400 MHz, CDCl_3_)


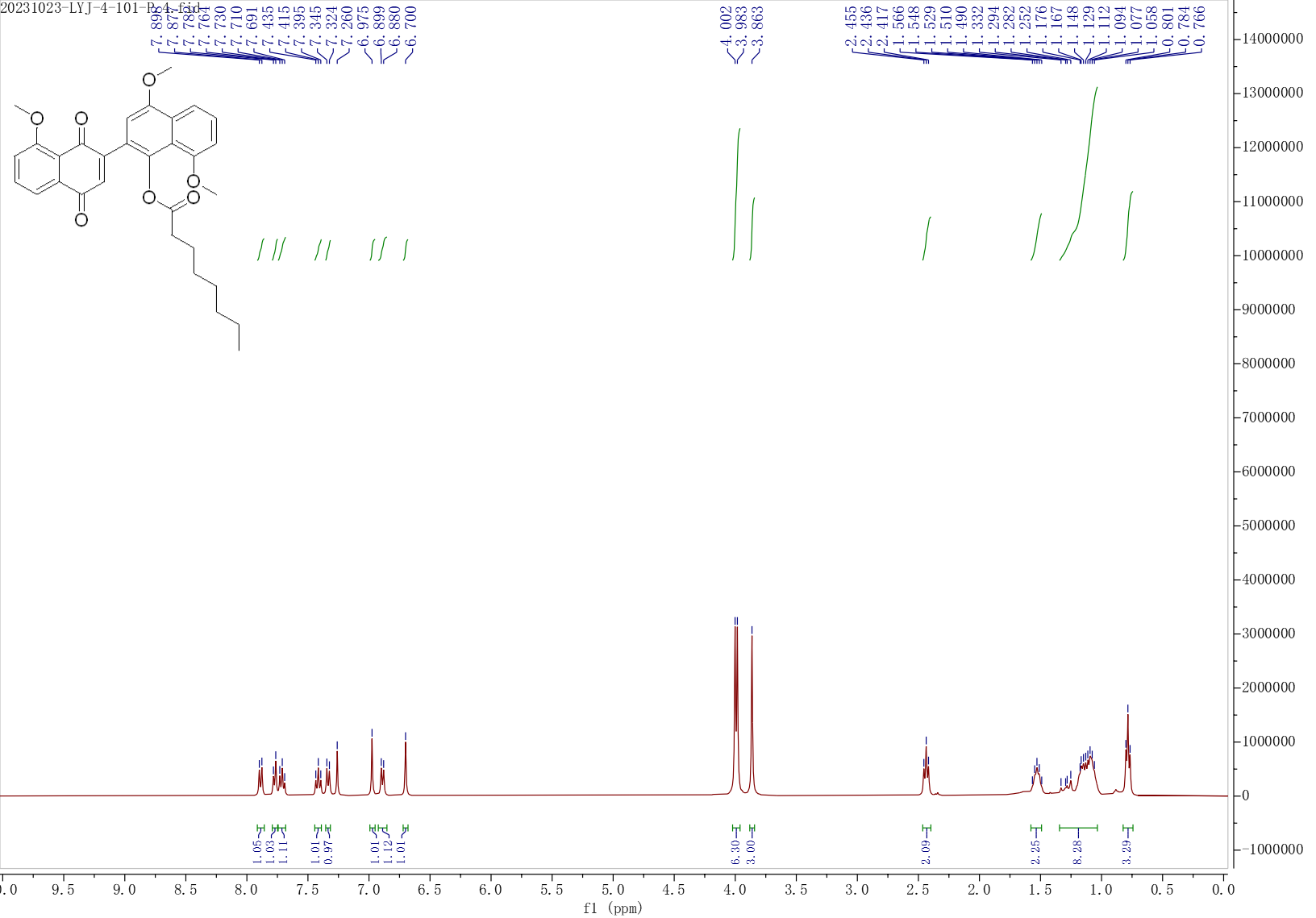


^13^C NMR of **10k** (100 MHz, CDCl_3_)


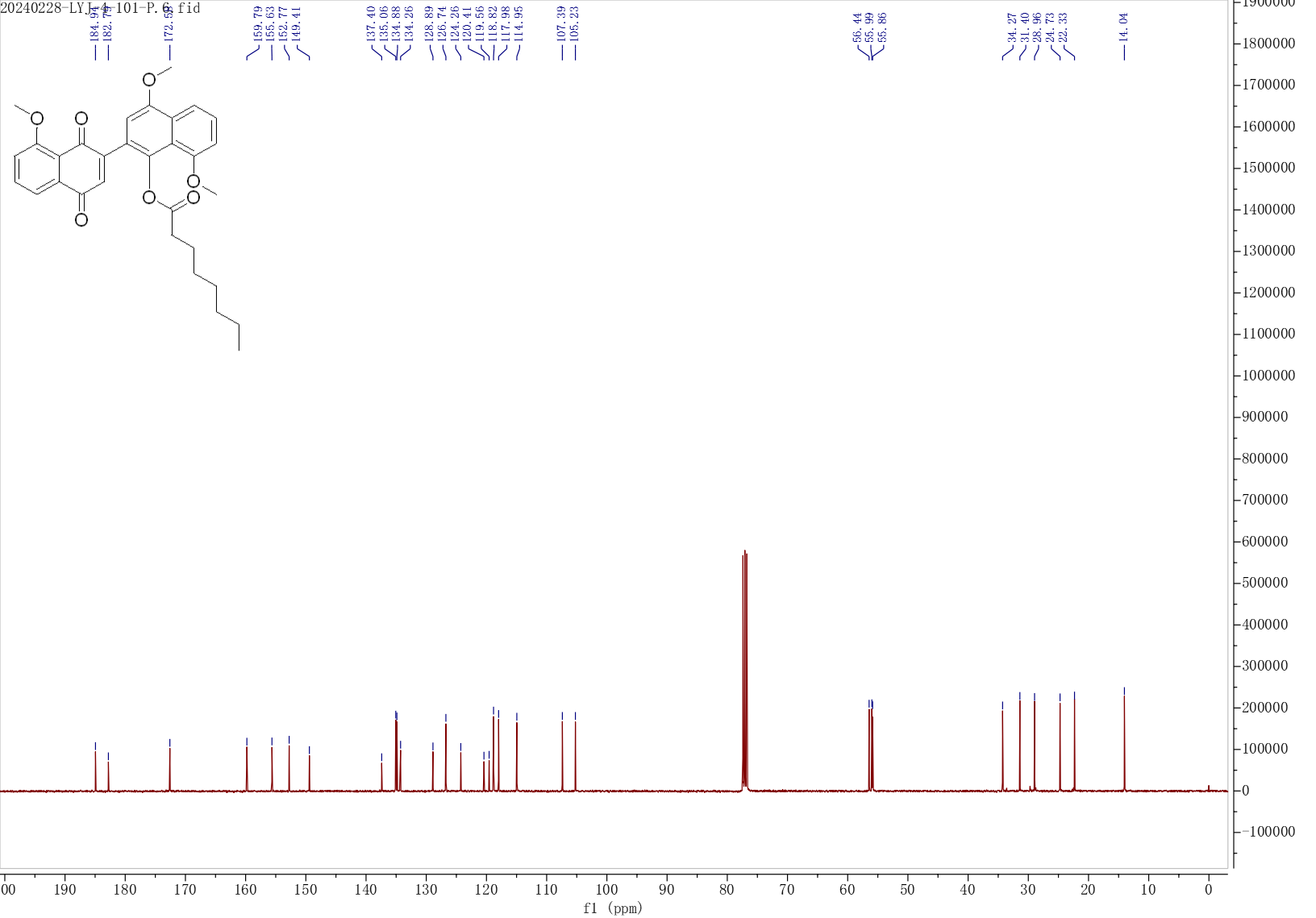


HRMS (ESI) of **10k**


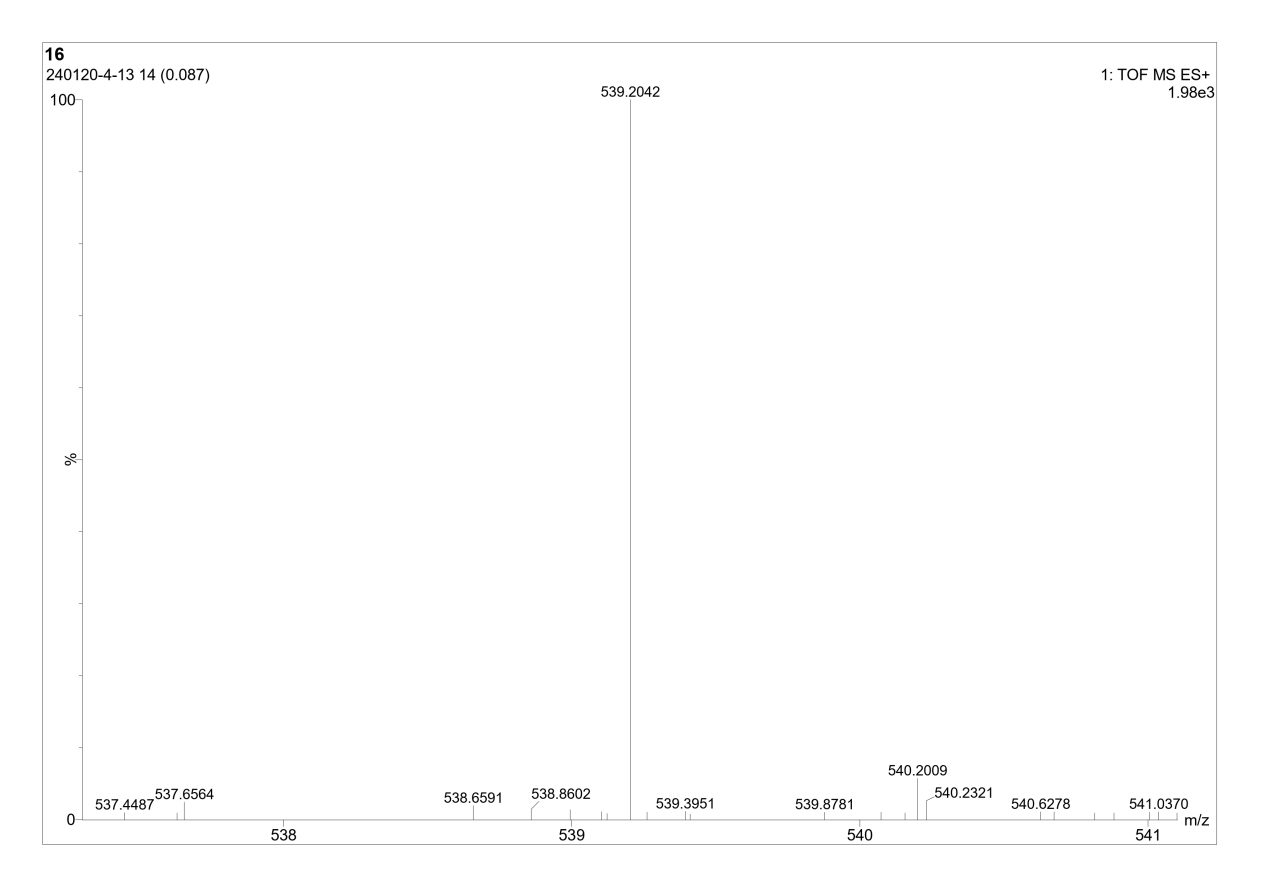


Compound **11a**:

^1^H NMR of **11a** (400 MHz, CDCl_3_)


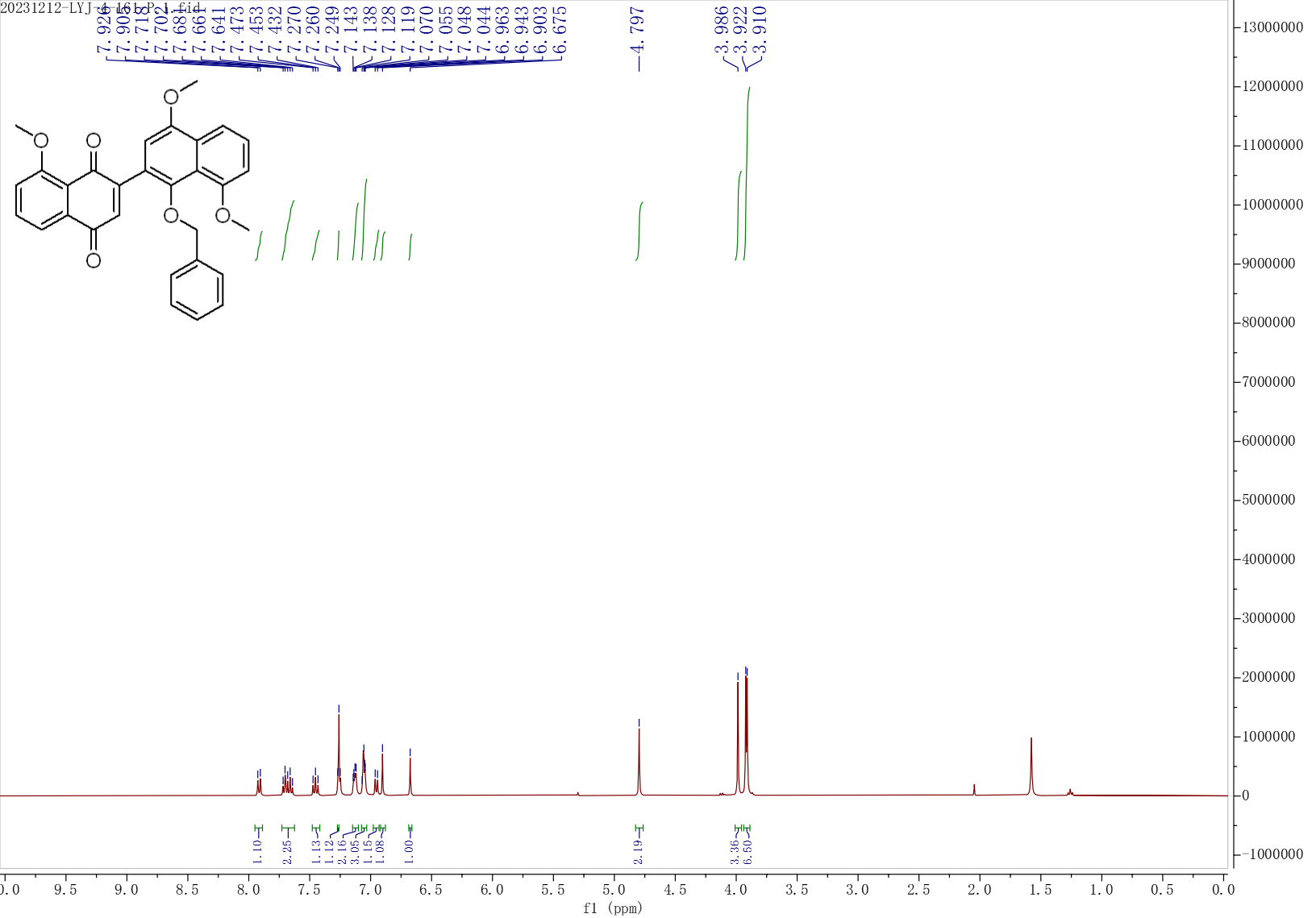


^13^C NMR of **11a** (100 MHz, CDCl_3_)


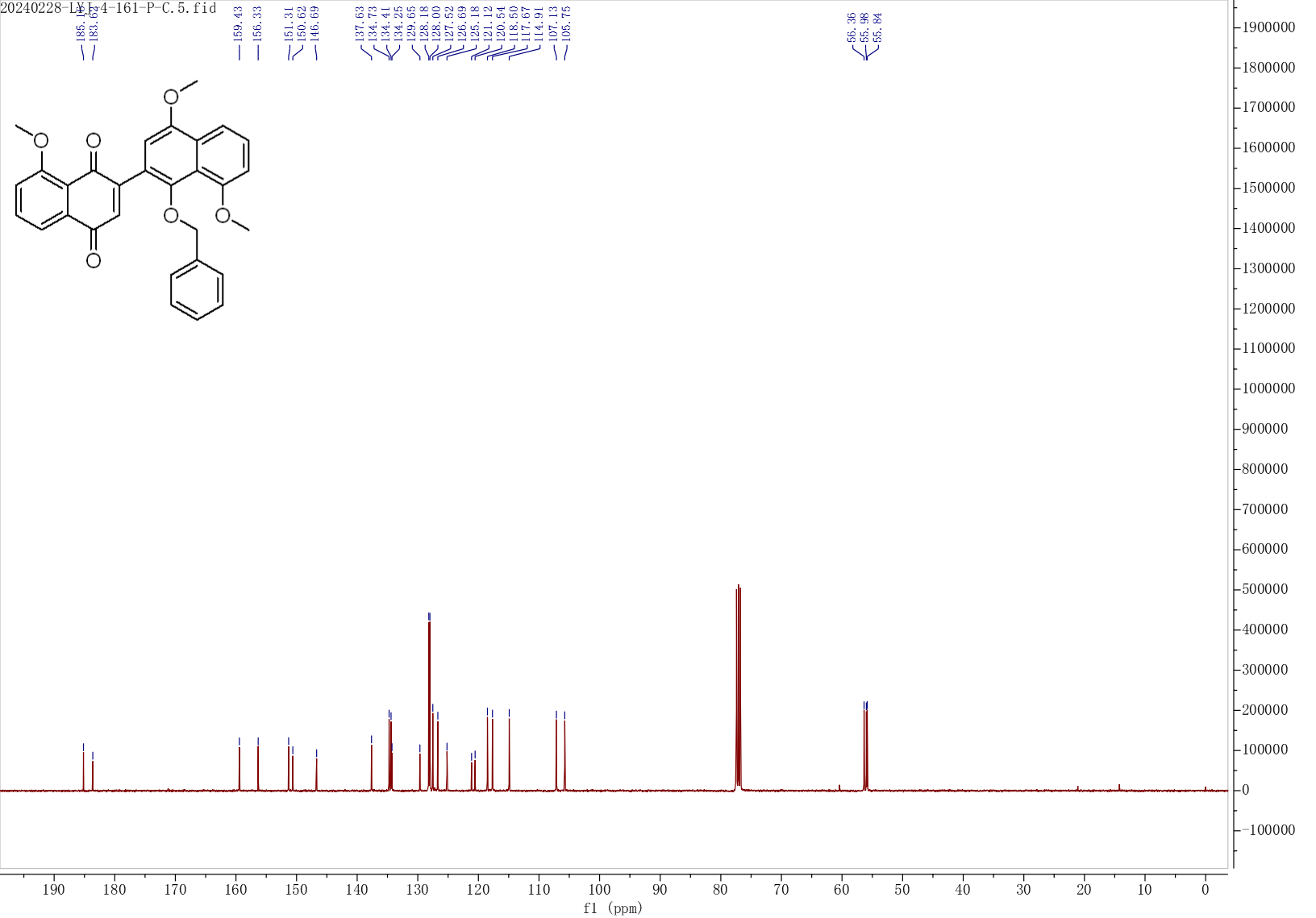


HRMS (ESI) of **11a**


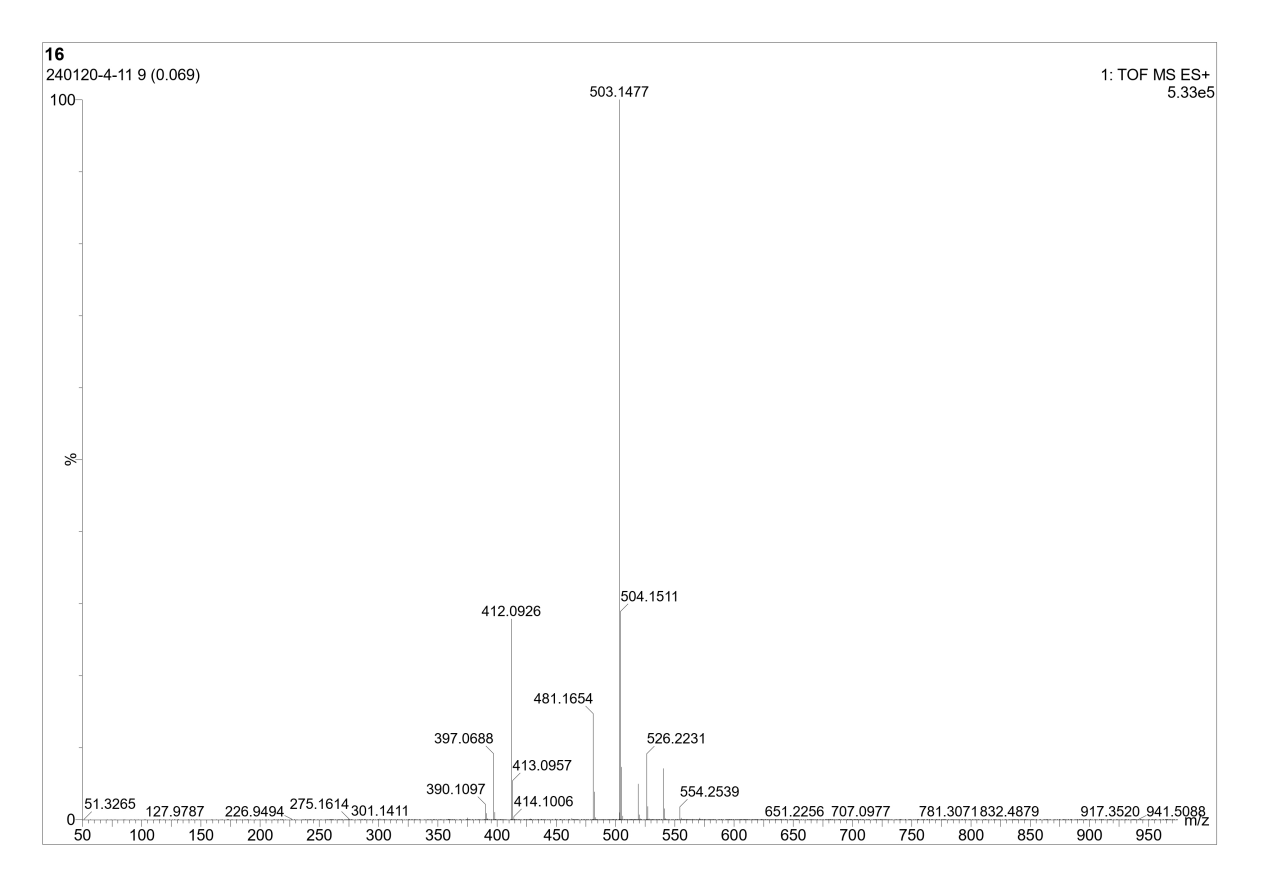


Compound **11b**:

^1^H NMR of **11b** (400 MHz, CDCl_3_)


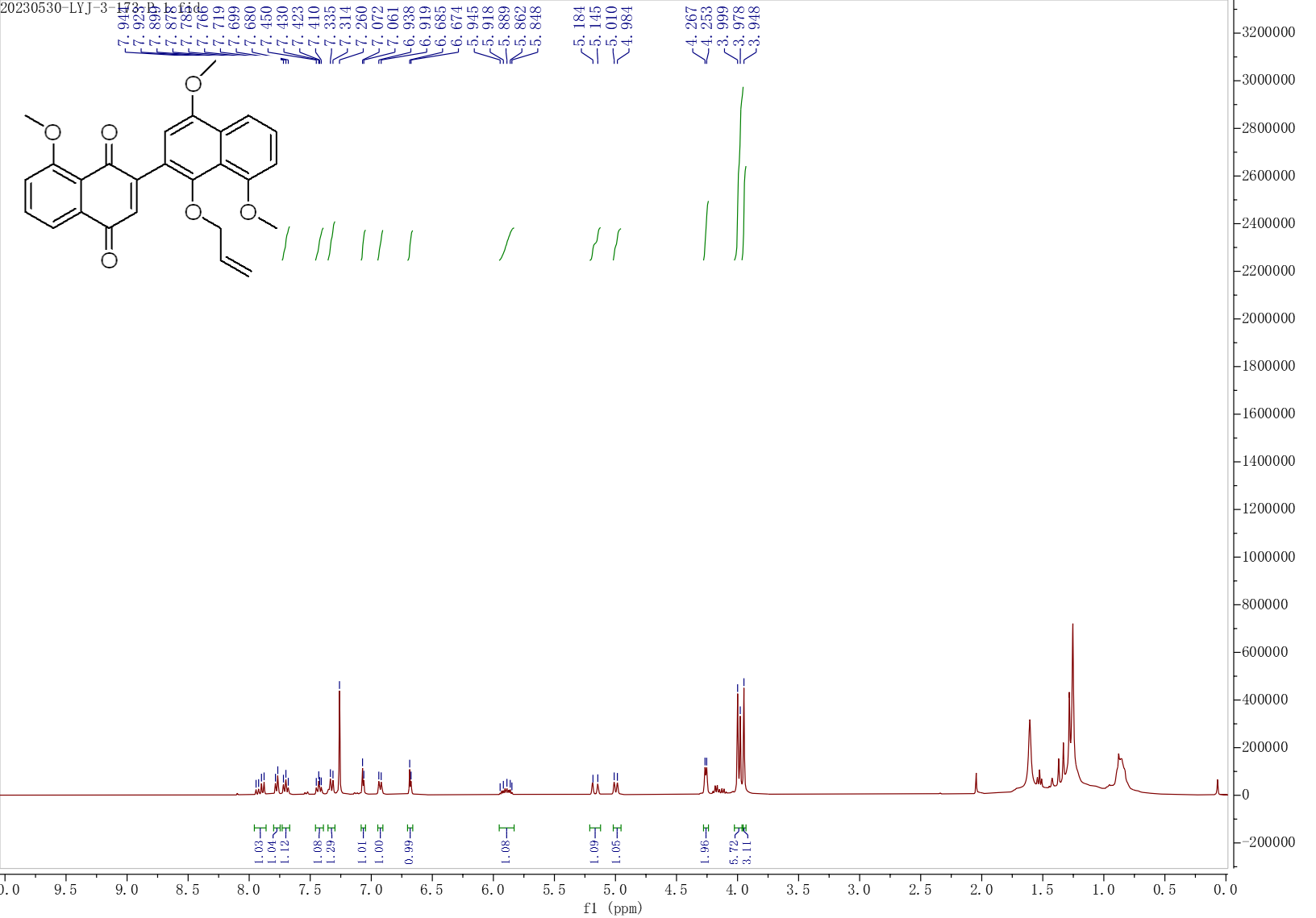


^13^C NMR of **11b** (100 MHz, CDCl_3_)


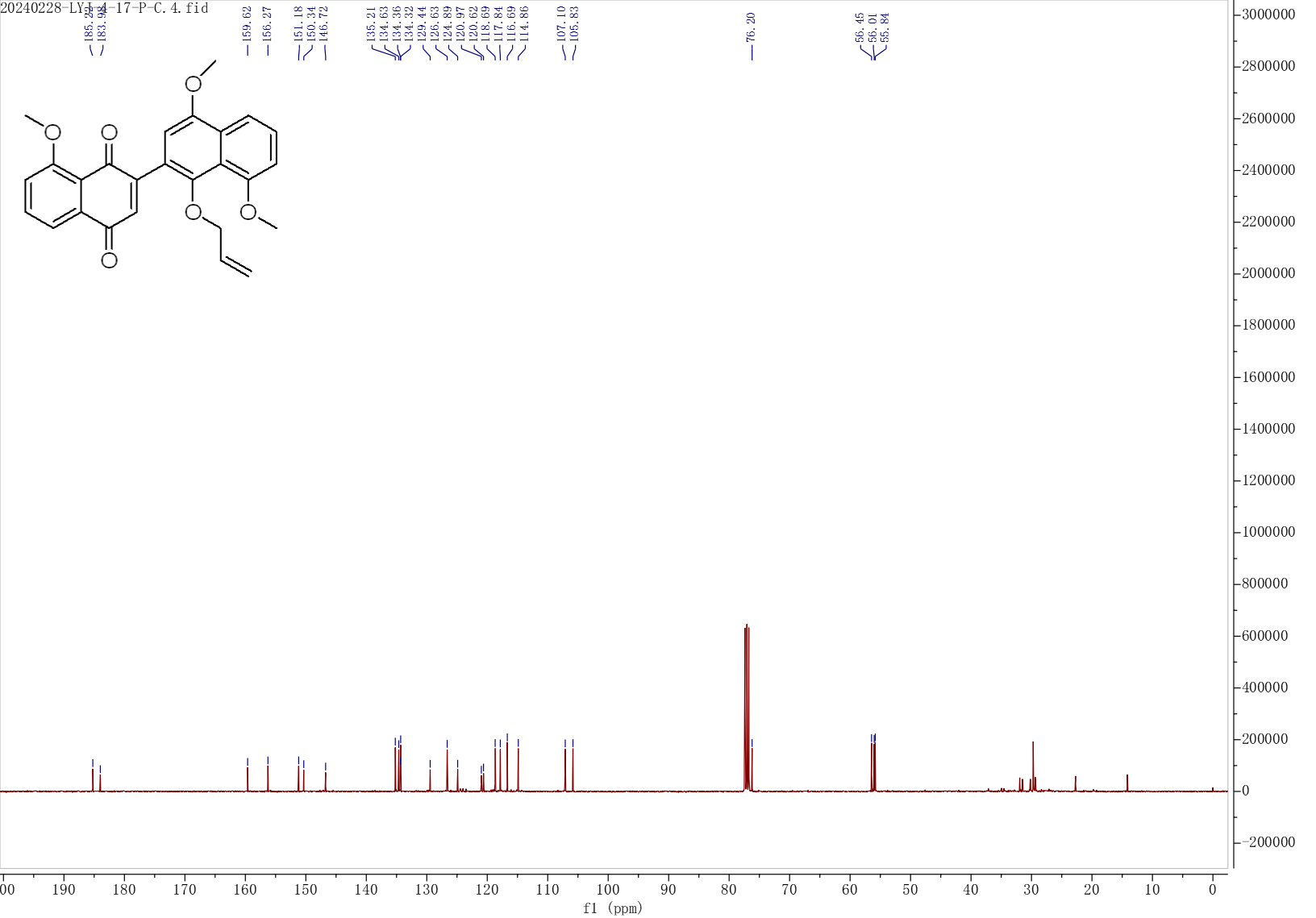


HRMS (ESI) of **11b**


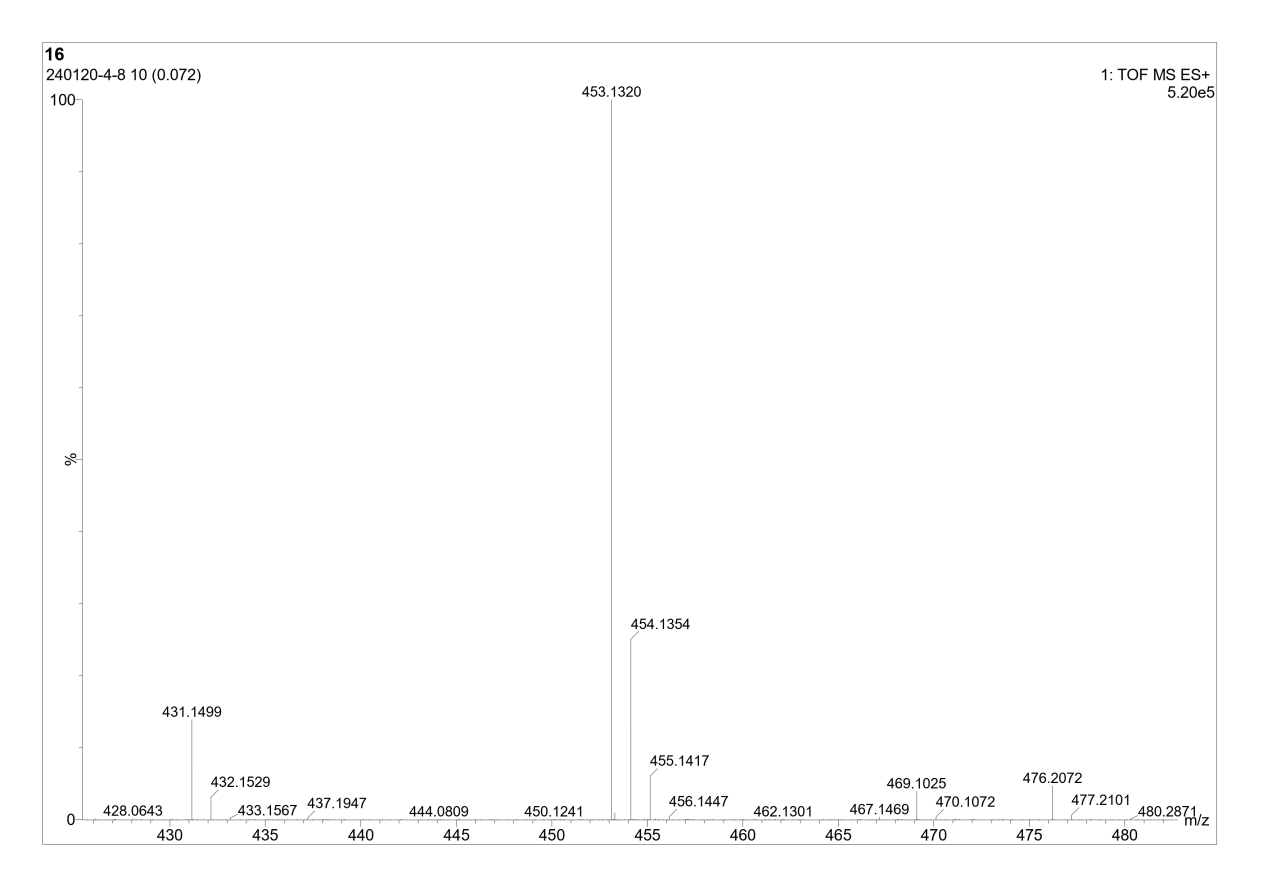


Compound **13**:

^1^H NMR of **13** (400 MHz, DMSO-d6)


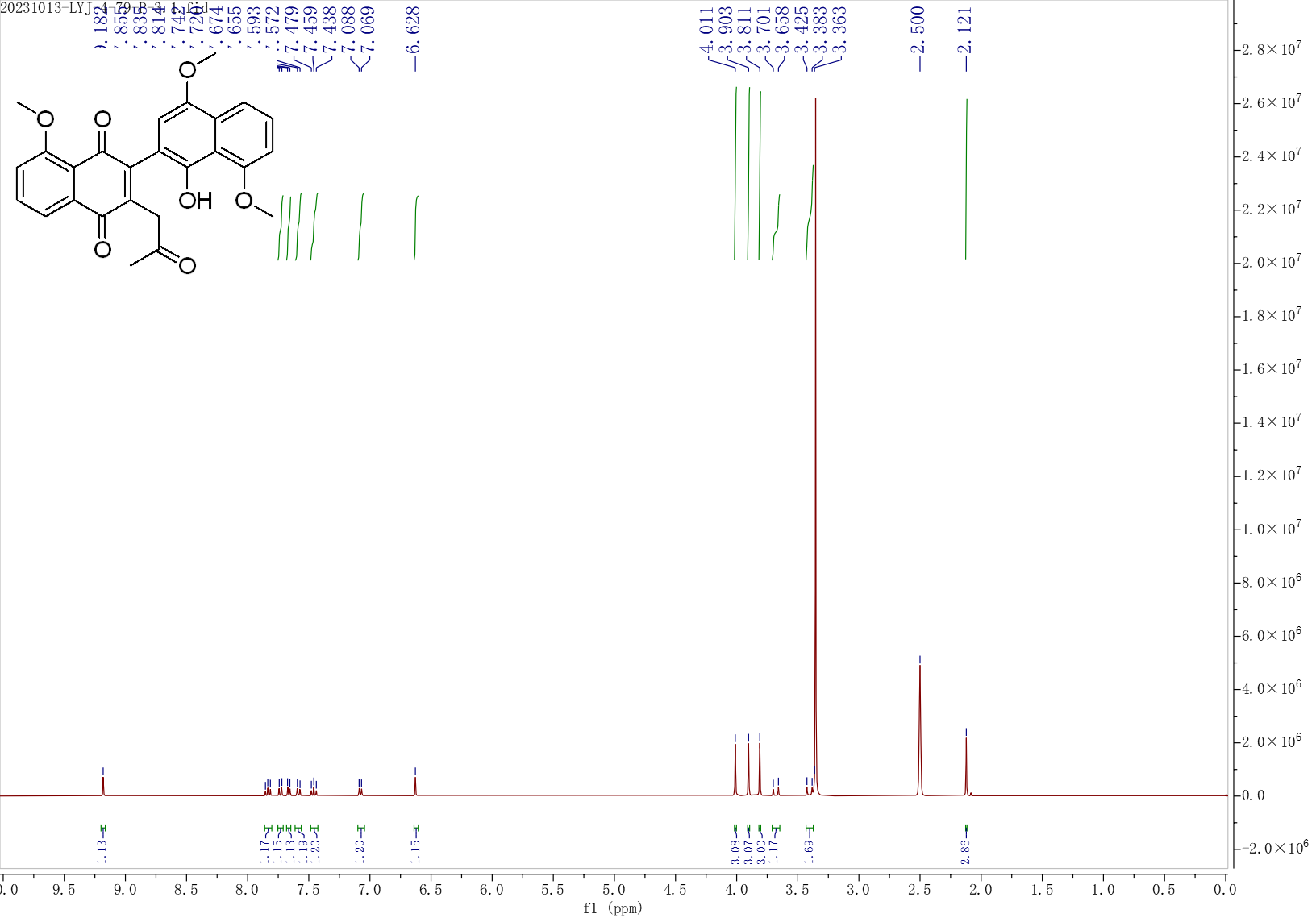


^13^C NMR of **13** (100 MHz, CDCl_3_)


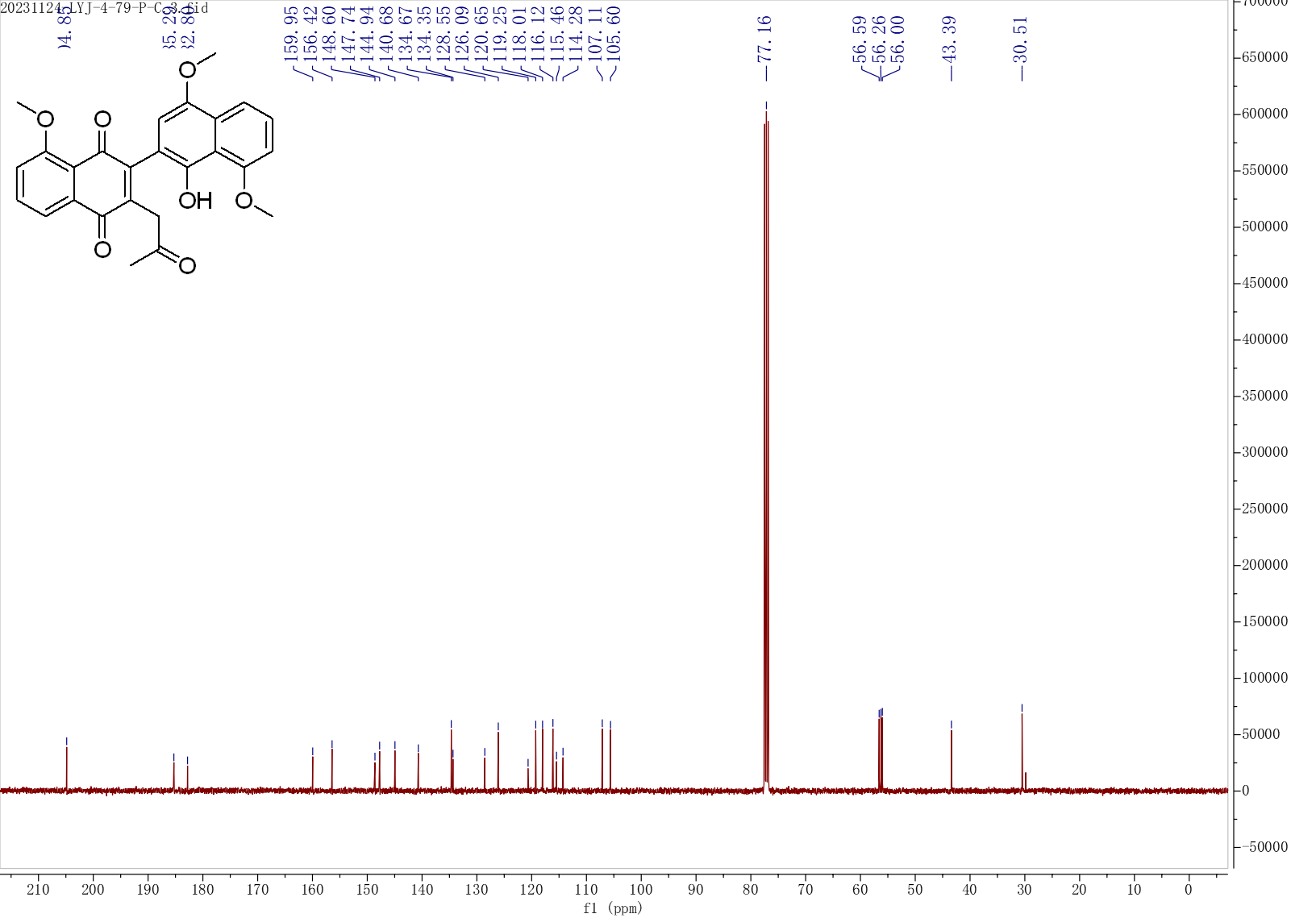


HRMS (ESI) of **13**


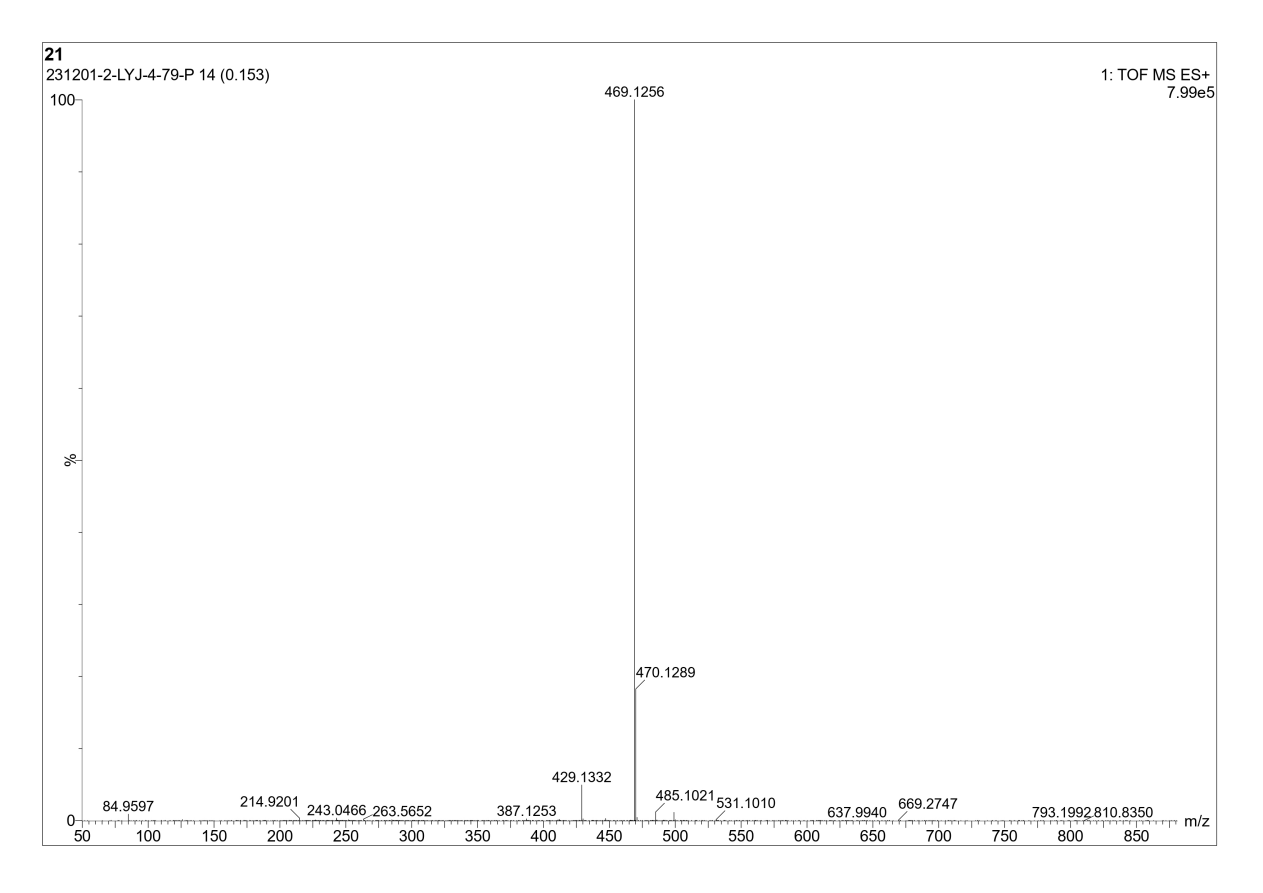


Compound **14**:

^1^H NMR of **14** (400 MHz, CDCl_3_)


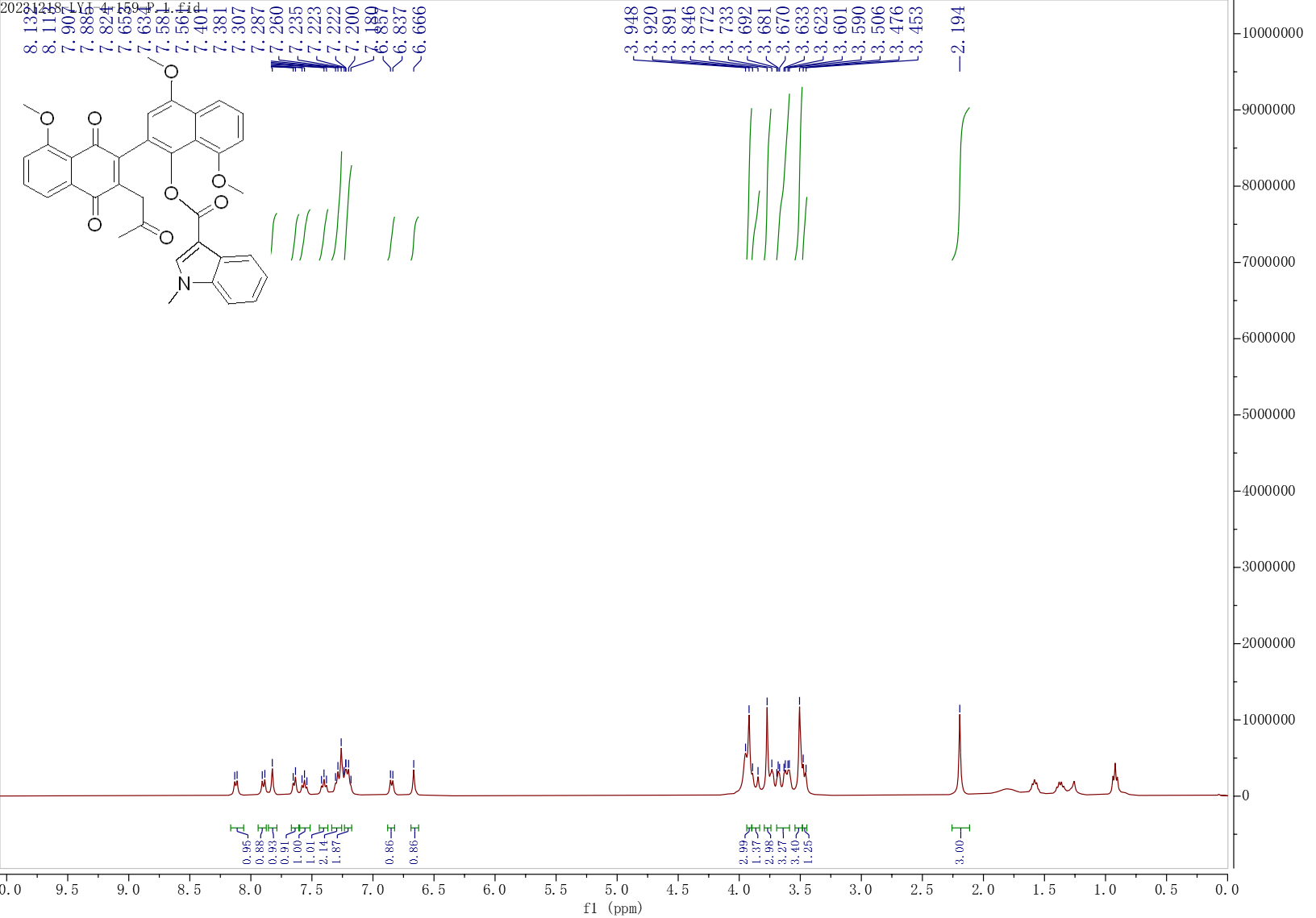


^13^C NMR of **14** (100 MHz, CDCl_3_)


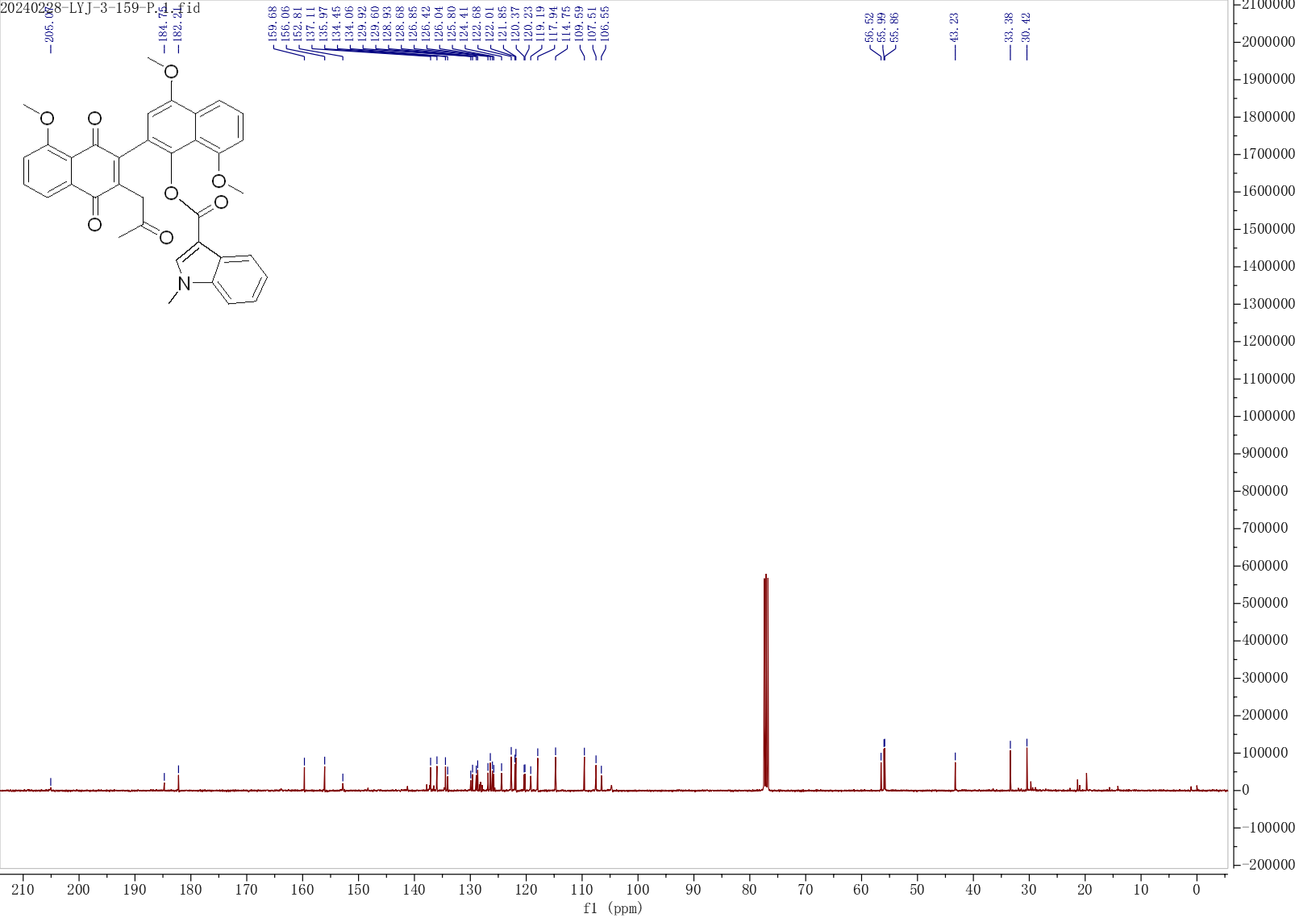


HRMS (ESI) of **14**


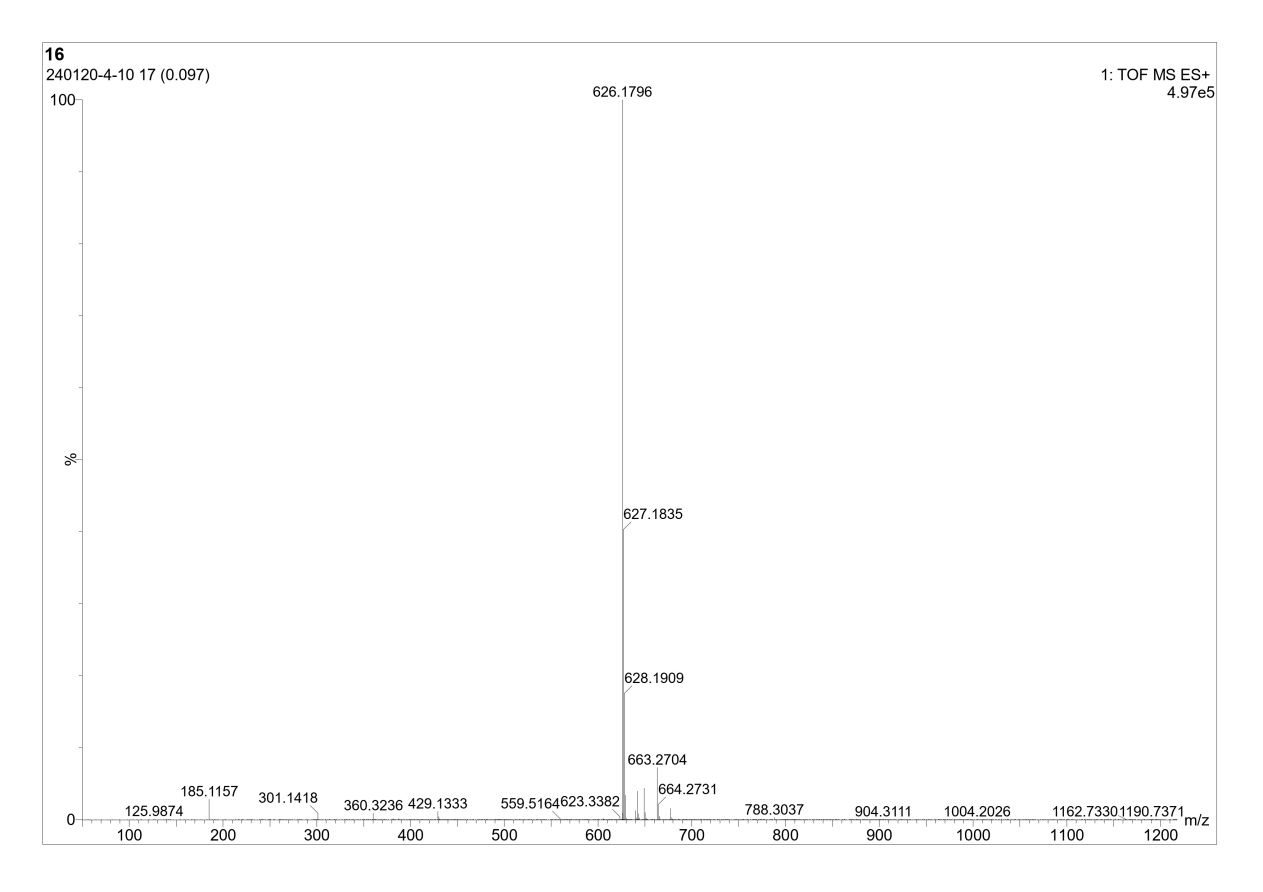


Compound **15**:

^1^H NMR of **15** (400 MHz, CDCl_3_)


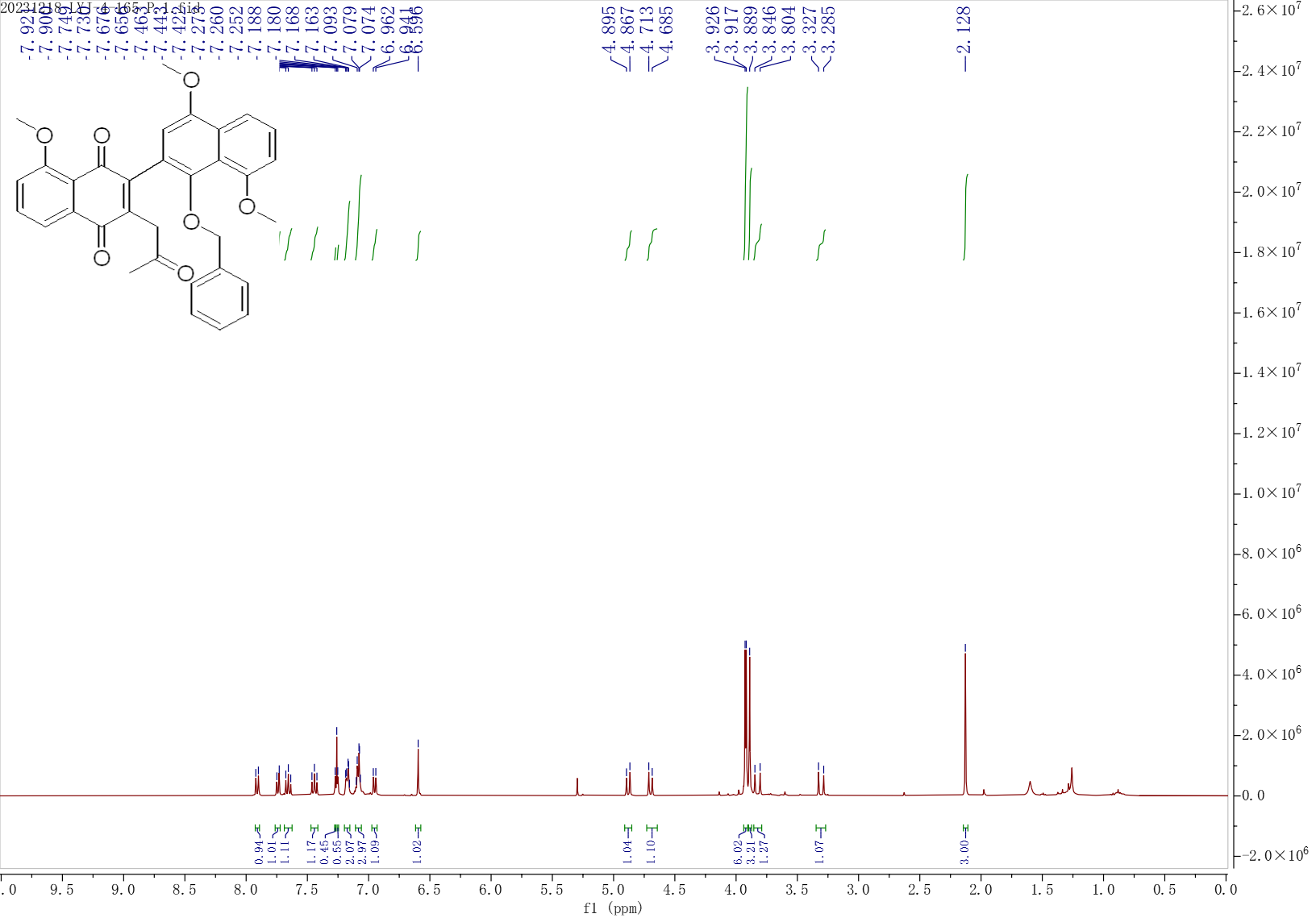


^13^C NMR of **15** (100 MHz, CDCl_3_)


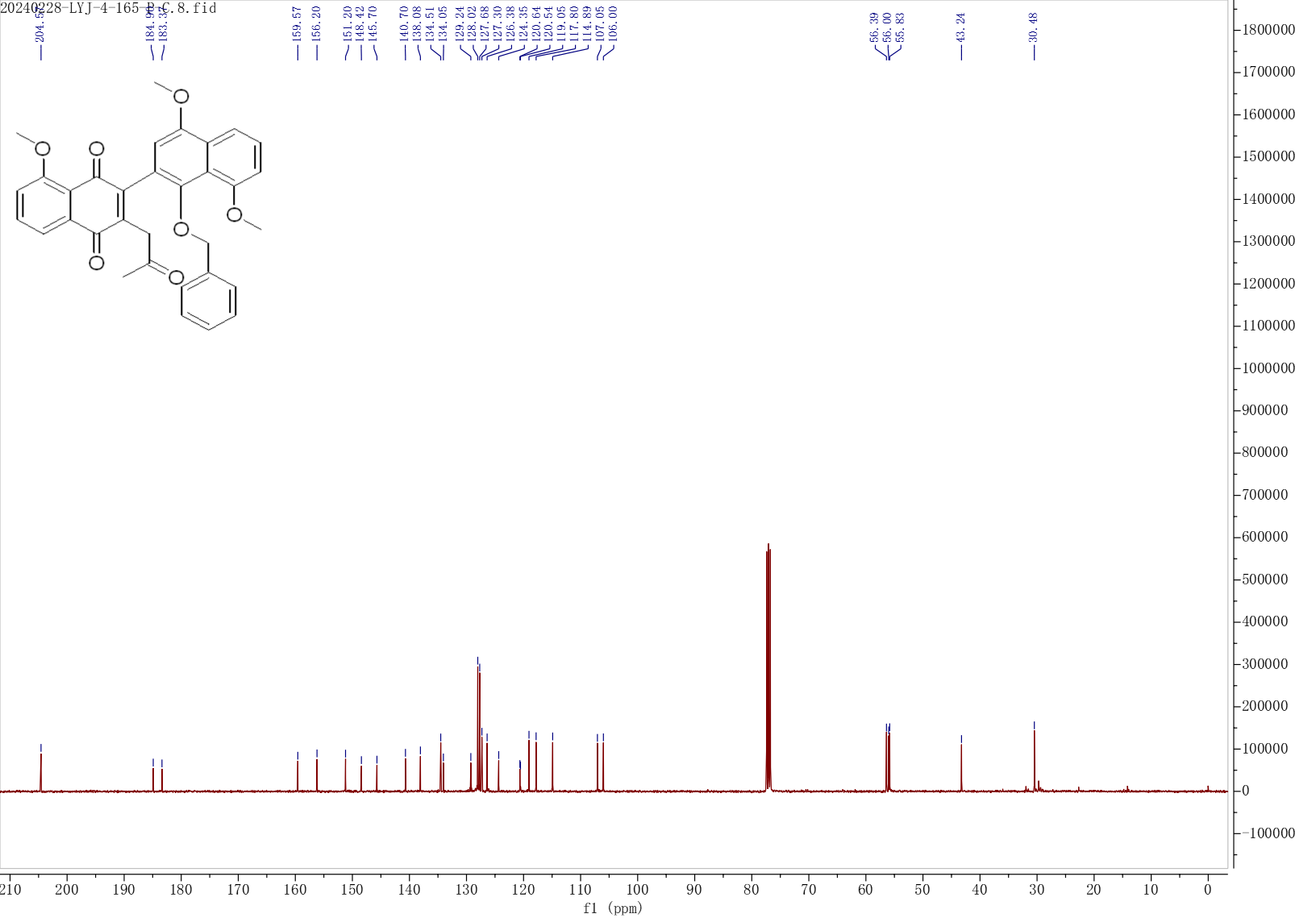


HRMS (ESI) of **15**


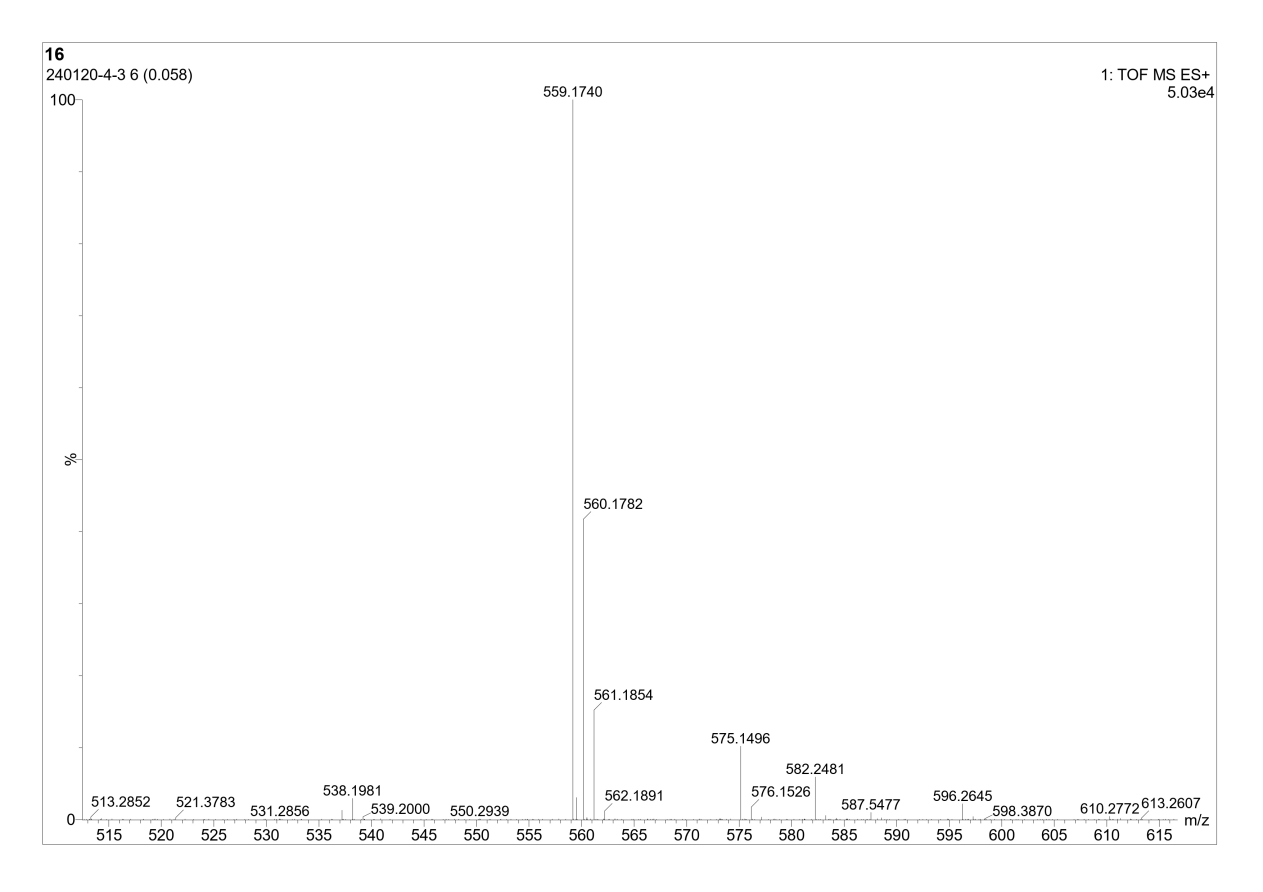

Supplement: Supplementary_data CLEAN.docx [file IENZ_A_2412865_SM5865.docx]
