# Supplementary figures and images for: Design, synthesis and biological evaluation of marine naphthoquinone-naphthol derivatives as potential anticancer agents
Source: J Enzyme Inhib Med Chem. 2024 Oct 15;39(1):2412865. doi: 10.1080/14756366.2024.2412865 (PMC11486183; doi:10.1080/14756366.2024.2412865)

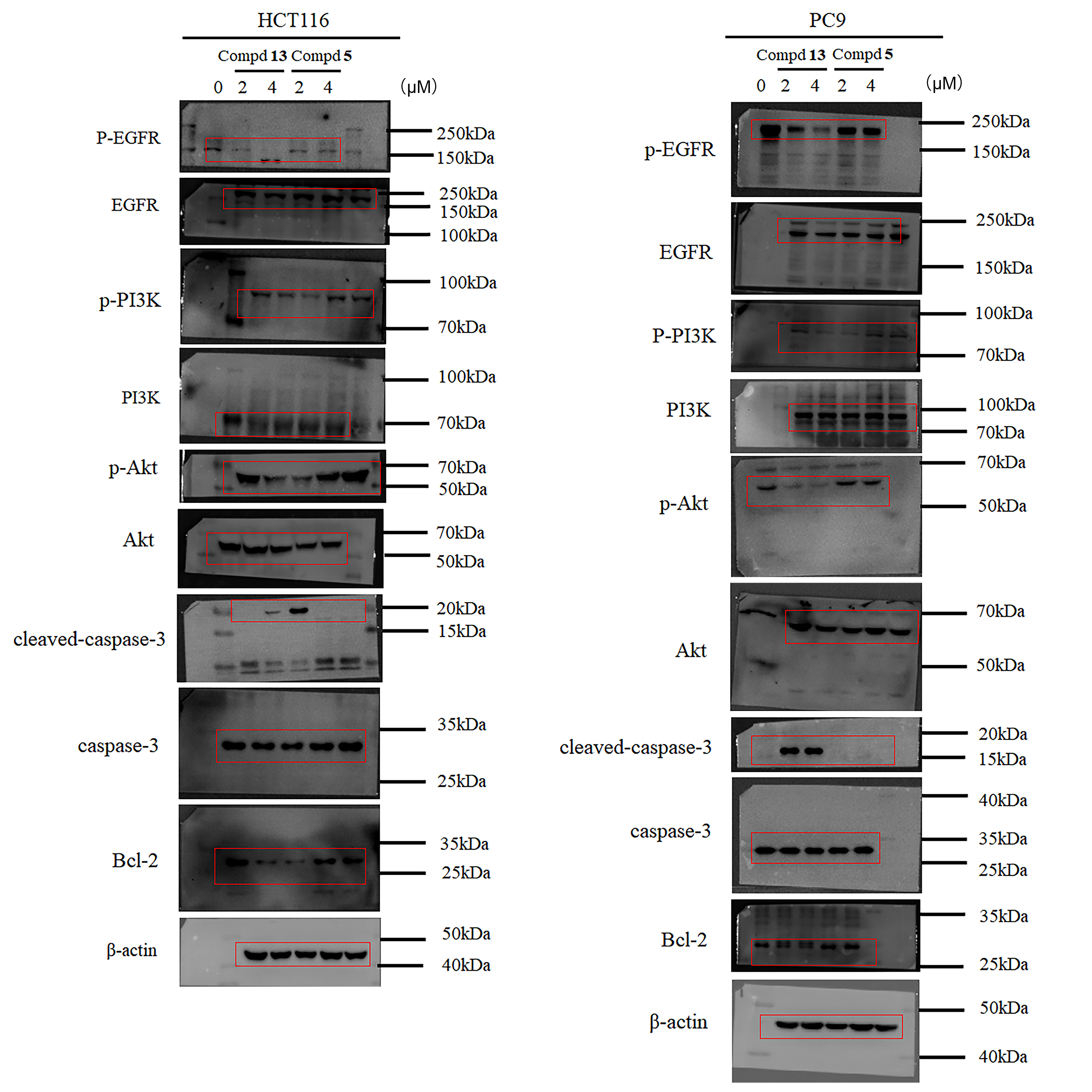

Supplement: Original Image for Figure 3A and 3C.tif [file IENZ_A_2412865_SM5864.tif]

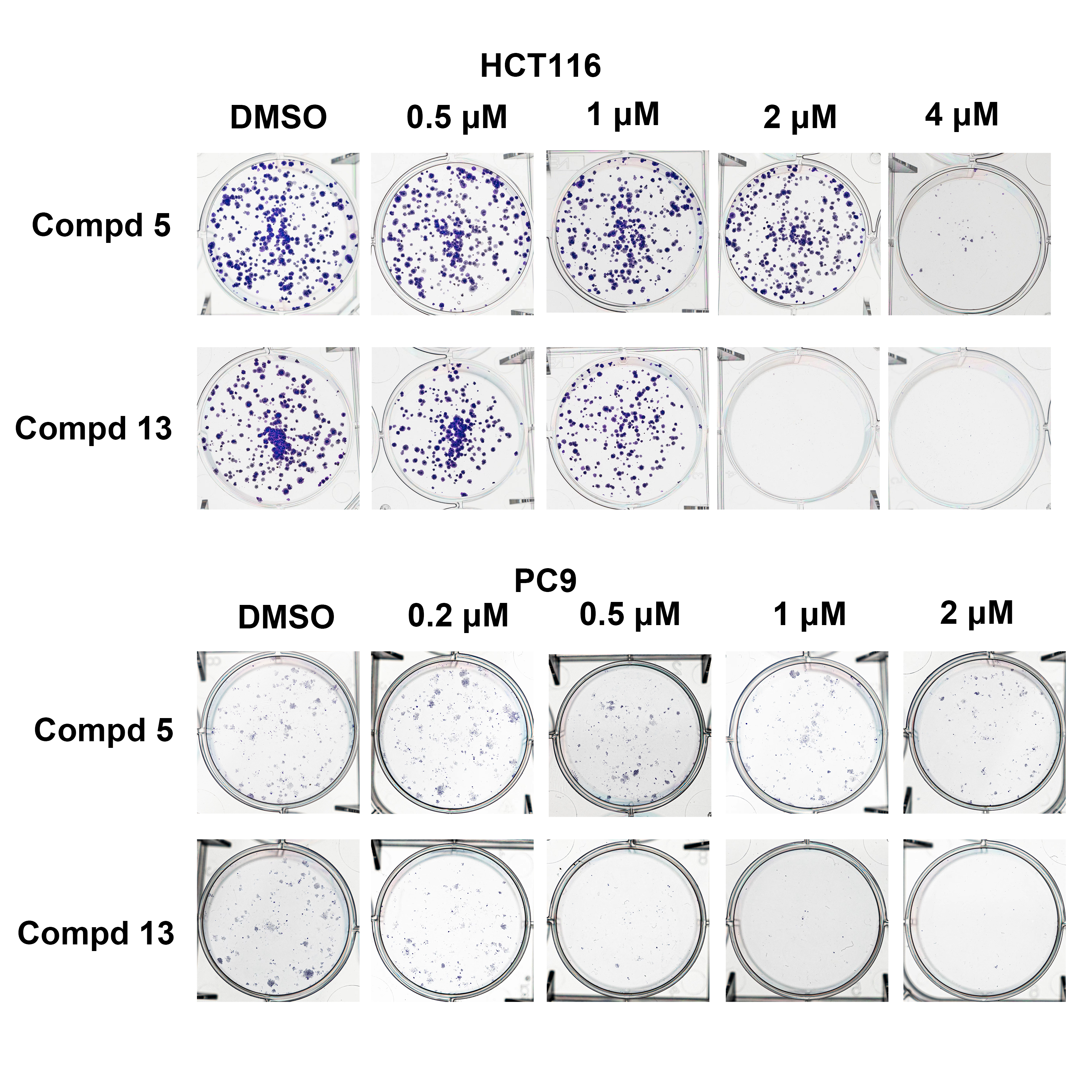

Supplement: Original Image for Figure 2C and 2E.tif [file IENZ_A_2412865_SM5863.tif]
